# Increased antibiotic resistance in preterm neonates under early antibiotic use

**DOI:** 10.1128/msphere.00286-24

**Published:** 2024-10-07

**Authors:** Amanda Ojeda, Oluwamayowa Akinsuyi, Kelley Lobean McKinley, Jessica Xhumari, Eric W. Triplett, Josef Neu, Luiz F. W. Roesch

**Affiliations:** 1 Department of Microbiology and Cell Science, Institute of Food and Agricultural Sciences, University of Florida, Gainesville, Florida, USA; 2 Department of Pediatrics, Division of Neonatology, University of Florida College of Medicine, Gainesville, Florida, USA; Duke Human Vaccine Institute, Durham, North Carolina, USA

**Keywords:** preterm infants, resistome, microbiome, meconium, stool

## Abstract

The standard use of antibiotics in newborns to empirically treat early-onset sepsis can adversely affect the neonatal gut microbiome, with potential long-term health impacts. Research into the escalating issue of antimicrobial resistance in preterm infants and antibiotic practices in neonatal intensive care units is limited. A deeper understanding of the effects of early antibiotic intervention on antibiotic resistance in preterm infants is crucial. This retrospective study employed metagenomic sequencing to evaluate antibiotic resistance genes (ARGs) in the meconium and subsequent stool samples of preterm infants enrolled in the Routine Early Antibiotic Use in Symptomatic Preterm Neonates study. Microbial metagenomics was conducted using a subset of fecal samples from 30 preterm infants for taxonomic profiling and ARG identification. All preterm infants exhibited ARGs, with 175 unique ARGs identified, predominantly associated with beta-lactam, tetracycline, and aminoglycoside resistance. Notably, 23% of ARGs was found in preterm infants without direct or intrapartum antibiotic exposure. Post-natal antibiotic exposure increases beta-lactam/tetracycline resistance while altering mechanisms that aid bacteria in withstanding antibiotic pressure. Microbial profiling revealed 774 bacterial species, with antibiotic-naive infants showing higher alpha diversity (*P* = 0.005) in their microbiota and resistome compared with treated infants, suggesting a more complex ecosystem. High ARG prevalence in preterm infants was observed irrespective of direct antibiotic exposure and intensifies with age. Prolonged membrane ruptures and maternal antibiotic use during gestation and delivery are linked to alterations in the preterm infant resistome and microbiome, which are pivotal in shaping the ARG profiles in the neonatal gut.

This study is registered with ClinicalTrials.gov as NCT02784821.

**IMPORTANCE:**

A high burden of antibiotic resistance in preterm infants poses significant challenges to neonatal health. The presence of antibiotic resistance genes, along with alterations in signaling, energy production, and metabolic mechanisms, complicates treatment strategies for preterm infants, heightening the risk of ineffective therapy and exacerbating outcomes for these vulnerable neonates. Despite not receiving direct antibiotic treatment, preterm infants exhibit a concerning prevalence of antibiotic-resistant bacteria. This underscores the complex interplay of broader influences, including maternal antibiotic exposure during and beyond pregnancy and gestational complications like prolonged membrane ruptures. Urgent action, including cautious antibiotic practices and enhanced antenatal care, is imperative to protect neonatal health and counter the escalating threat of antimicrobial resistance in this vulnerable population.

## INTRODUCTION

Antibiotic resistance is a formidable global health challenge impacting hospital and community settings, contributing to an estimated 214,000 neonatal mortalities annually (1–3). Preterm infants stand out among these vulnerable populations due to their inherent vulnerability, marked by intestinal and immune immaturity and reduced intestinal microbial diversity (4, 5). This underdevelopment renders preterm infants highly prone to sepsis, a predominant contributor to mortality and morbidity in neonatal populations globally. Early-onset sepsis (EOS), an infection that manifests within the first 72 hours after birth, is particularly concerning. EOS typically results from vertical transmission, occurring either during intrapartum or antenatally in cases where the mother has a bloodstream infection or prolonged exposure to infection and inflammation in the womb (6, 7). Key risk factors for EOS include extended periods of ruptured membranes, preterm delivery, clinical signs of maternal infection in the womb (chorioamnionitis), and maternal colonization by Group B *Streptococcus* (GBS) (6). Nonetheless, accurately diagnosing EOS presents difficulties due to the ambiguous nature of initial clinical symptoms, the limited predictive value of diagnostic tests, and the potentially dire outcomes associated with postponing antibiotic therapy (8).

To combat the risk of EOS—a fatal threat to neonates—empiric antibiotics are often administered within 24 hours of birth, even in the absence of culture-confirmed infections (9). As a result, 30-40% of uninfected neonates are exposed to antibiotics unnecessarily for each infant subsequently confirmed to have EOS (10). This proactive approach to antibiotics can exact a long-term toll on the developing gut microbiome by altering the early gut colonizers, leading to unintended consequences for the infant’s health (11, 12). Furthermore, the repercussions of sustained antibiotic use on the prevalence of antimicrobial resistance genes and microbiome composition in this high-risk population must be more adequately elucidated. While antibiotic resistance is a rising global concern, there are notable gaps in research regarding antibiotic utilization in premature infants. Limited studies have focused on characterizing genes, and understanding the factors contributing to the variability in antibiotic utilization practices among neonatal intensive care units (NICUs) remains an ongoing challenge.

Understanding the impact of routinely used antibiotics on antimicrobial resistance in preterm infants is paramount. The Routine Early Antibiotic Use in SymptOmatic Preterm Neonates (REASON) study at the University of Florida investigated the effects of antibiotics on the gut microbiome, metabolome, and inflammatory environment of preterm infants (11). Their initial findings revealed that withholding antibiotics did not lead to a significant increase in neonatal mortality or morbidity. This pivotal observation challenges conventional assumptions in clinical practice. Russell et al. (13) also showed that antibiotic administration did not significantly affect fecal microbiome diversity in a subset of individual premature infants over time, indicating underlying resistance mechanisms. In another subset, 26% of preterm infants administering dual-combination antibiotics (gentamicin and ampicillin) led to the increase of *Enterobacteriaceae*, a family with many opportunistic pathogens that drive antibiotic resistance (13).

Similar observations have been reported in other premature infant-oriented studies conducted in Midwest United States NICUs, where the gut microbiome showed an increase in potential pathogen abundance following antibiotic administration, mainly when ampicillin or gentamicin were administered (14). Prolonged exposure to antibiotics has been associated with reduced overall microbial diversity in which a depletion in beneficial microorganisms like *Bifidobacteriaceae*, an early gut colonizer efficient at fermenting human milk, oligosaccharides to produce short-chain fatty acids, an essential nutritional source in the gut. This can lead to an increase in pathogenic *Enterobacteriaceae*, commonly associated with antimicrobial resistance, increasing the risk of sepsis (15, 16). These preliminary results reinforce the critical need for further investigation into the impact of antibiotics on preterm infants, with particular emphasis on understanding the development of resistance mechanisms in this vulnerable population. Given that interventions during this sensitive timeframe can have long-lasting effects on health, it is imperative to delve deeper into the consequences of antibiotic use in preterm infants. By understanding the impact of antibiotics on resistance development, we can develop more precise and practical strategies to mitigate antibiotic resistance in this population. Antibiotic resistance occurs when bacteria evolve and develop new strategies to counteract antibiotics by carrying antibiotic resistance genes (ARGs), thus allowing bacteria to overcome the antibiotic effects, making them no longer effective in treating bacterial infections (17). The set of ARGs present in a population of bacteria is known as the resistome. This study aims to detect and quantify the prevalence of ARGs from a subset of preterm infants enrolled in the REASON study, specifically in preterm infants’ meconium and stool samples. This investigation assesses whether the routine use of antibiotics in preterm infants as standard care increases the prevalence of ARG genes in the resistome and how it alters the infant microbiome.

## MATERIALS AND METHODS

### Study overview, stool sampling, and survey data collection

In this retrospective study, metagenomic sequencing was conducted to detect and quantify ARGs in preterm infant meconium and stool samples from participants enrolled in the REASON study. The REASON study was conducted from January 2017 to 2019 at the University of Florida and is the first trial to randomize neonates to receive or not receive antibiotics after the first 48 hours after birth (11). The study was approved by the Institutional Review Board of the University of Florida (IRB 201501045). Meconium (the first stool typically passed within 24–48 hours post-birth) and stool samples were collected from preterm infants weekly after birth until discharged from the hospital. Preterm infant’s maternal medical history, treatment, and feeding information were recorded along with aliquots from original stool samples from 91 neonates (<33 weeks gestation). Samples were stored at −80°C since the preliminary research investigating the impact of antibiotic usage on microbial diversity in the neonatal gut microbiota (13).

**Fig 1:**
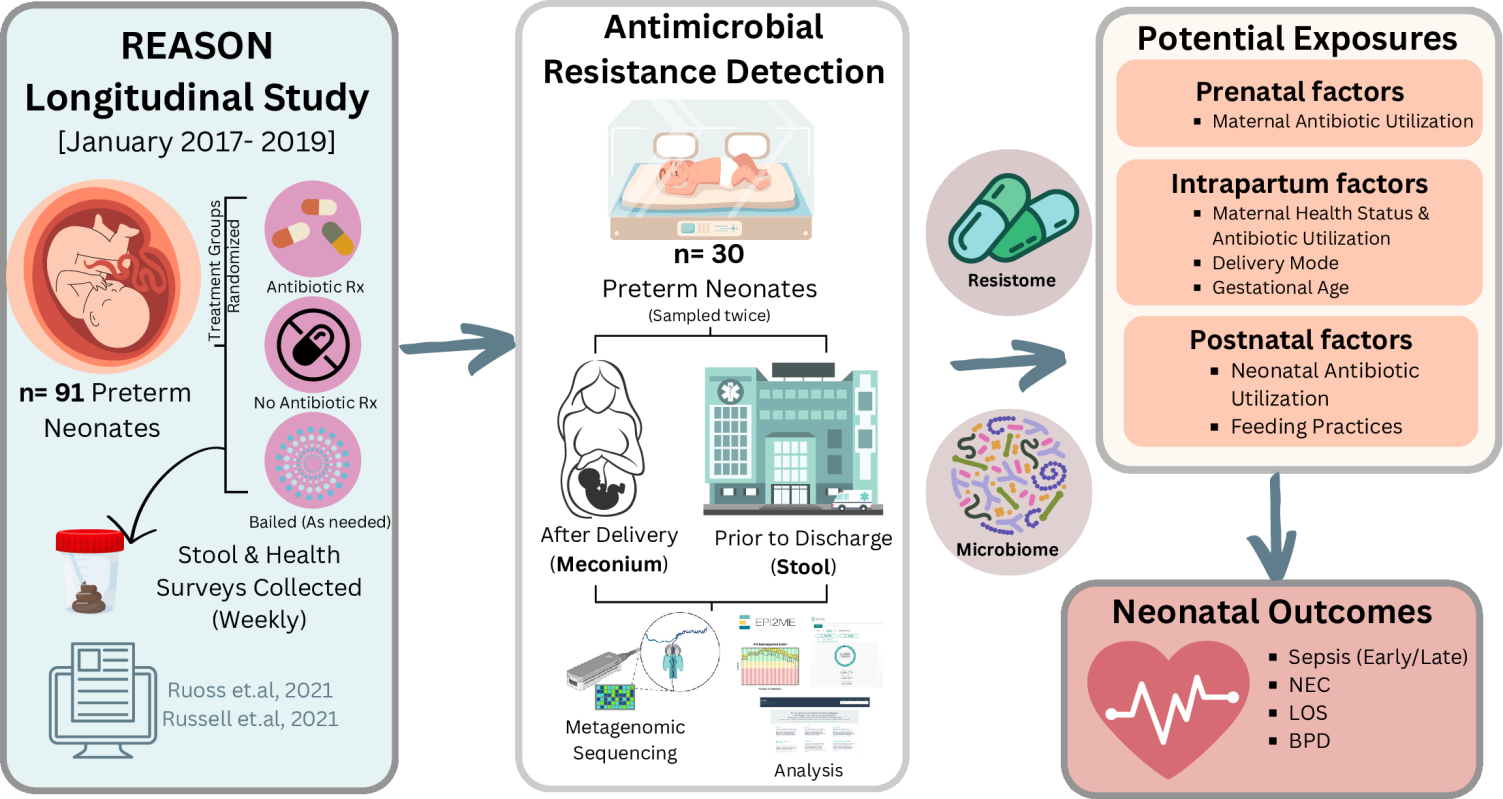
Flowchart illustrating the analysis methods for the REASON longitudinal cohort and antibiotic resistance study, including the investigation of potential exposures linked to elevated detection of ARGs. The left side of the figure highlights the types of samples and metadata collected from the REASON study (*n* = 91 preterm neonates). In this study, we evaluated a subset of samples from 30 preterm neonates across two sampling time points, including meconium and stool samples collected before discharge for the presence of ARGs. Potential determinants driving ARG detection and neonatal health outcomes were analyzed. This figure was made using Canva and BioRender.

**Fig 2:**
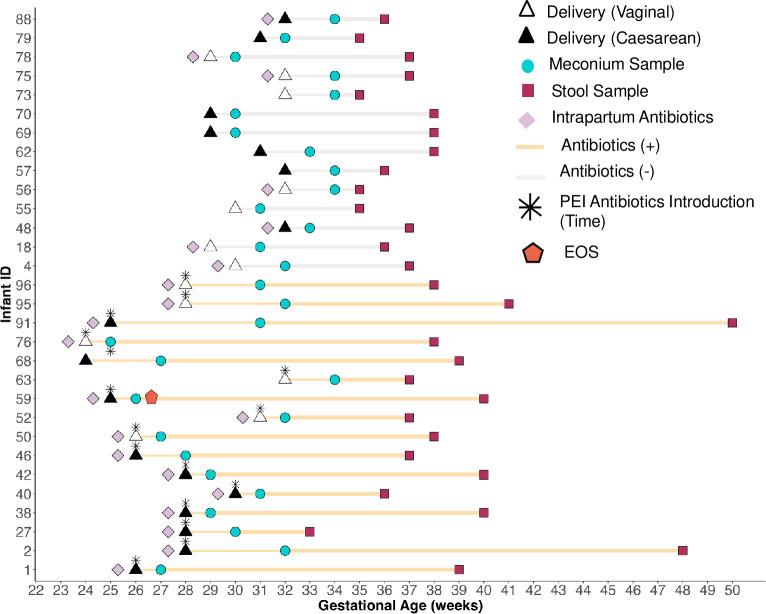
Sampling schematic and participant demographics. A total of 30 infants were included, and stool samples were collected at two time points: immediately after birth (base stool) and just before hospital discharge (end stool). Among the 30 neonates, 16 received antibiotic treatment at birth (yellow line), while 14 did not (gray line). Neonates who did not receive antibiotics at birth remained free from antibiotic exposure throughout the study. The gestational age of the infants ranged from 24 to 32 weeks, with a median age of 29 weeks and a standard deviation (SD) of 2.55. Additional information included mode of delivery, antibiotic administration to mothers (with 21 mothers receiving antibiotics during delivery), and the timing of stool sample collection (marked as a blue circle for the first stool after birth and a red square prior to discharge). PEI stands for preterm infant. Infant ID refers to a unique identifier assigned to each participant throughout this study.

Based on sample availability, 30 preterm infants were selected for inclusion in our study (Fig. 1). Of the 30 preterm infants, meconium (first stool passed after birth) and stool collected (before hospital release, ranging from 2 to 23 weeks after birth) from symptomatic preterm infants who were administered antibiotics (*n* = 16 treatment group) and asymptomatic preterm infants who did not receive antibiotics (*n* = 14 control group) were analyzed in this study (Fig. 2; Table 1).

**TABLE 1:** Clinical characteristics of preterm infants analyzed in this study (*n* = 30)^*a*^

Variables	Neonatal treatment groups	
	Antibiotics 53% (*n* = 16)	Control 46% (*n* = 14)
Infant sex		
Male	62.5% (*n* = 10)	35.7% (*n* = 5)
Female	37.5% (*n* = 6)	64.3% (*n* = 9)
Gestational age		
Extremely preterm birth (PB) (<28 weeks)	43.8% (*n* = 7)	0% (*n* = 0)
Very PB (28 to <32 weeks)	50% (*n* = 8)	57% (*n* = 8)
Moderately PB (32 to <37 weeks)	6.2% (*n* = 1)	43% (*n* = 6)
Delivery mode and maternal health		
Vaginal	43.8% (*n* = 7)	50% (*n* = 7)
Cesarean	56.2% (*n* = 9)	50% (*n* = 7)
Rupture of membrane (ROM) length < 18 hours	68.7% (*n* = 11)	64.3% (*n* = 9)
ROM length > 18 hours	31.3% (*n* = 5)	35.7% (*n* = 5)
GBS (+)	6.3% (*n* = 1)	14.3% (*n* = 2)
GBS (−)	56.2% (*n* = 9)	64.3% (*n* = 9)
GBS (unknown)	37.5% (*n* = 6)	21.4% (*n* = 3)
Chlamydia (−)	94.7% (*n* = 15)	93% (*n* = 13)
Magnesium sulfate (+)	100% (*n* = 16)	79% (*n* = 11)
Magnesium sulfate (−)	0% (*n* = 0)	21% (*n* = 3)
Birth weight		
≤1,128 g	75% (*n* = 12)	21.4% (*n* = 3)
>1,128 g	25% (*n* = 4)	78.6% (*n* = 11)
Maternal antibiotic utilization		
ANC antibiotics (+)	56% (*n* = 9)	78% (*n* = 11)
Intrapartum antibiotics (+)	88% (*n* = 14)	50% (*n* = 7)
PEI antibiotic utilization		
Gentamicin (+)	100% (*n* = 16)	0% (*n* = 0)
Ampicillin (+)	100% (*n* = 16)	0% (*n* = 0)
Azithromycin (+)	12.5% (*n* = 2)	0% (*n* = 0)
Metronidazole (+)	37.5% (*n* = 6)	0% (*n* = 0)
Piperacillin (+)	50% (*n* = 8)	0% (*n* = 0)
Cefotaxime (+)	25% (*n* = 4)	0% (*n* = 0)
Ceftazidime (+)	18.7% (*n* = 3)	0% (*n* = 0)
Vancomycin (+)	50% (*n* = 8)	0% (*n* = 0)
PEI feeding practices		
No enteral feeding (+)	94.7% (*n* = 15)	79% (*n* = 11)
Fed mother and donor breastmilk (+)	68.7% (*n* = 11)	28.6% (*n* = 4)
Fed mother breastmilk (+)	75% (*n* = 12)	71% (*n* = 10)
Fed donor breastmilk (+)	68.7% (*n* = 11)	28.6% (*n* = 4)
Fed formula (+)	81.3% (*n* = 13)	85.7% (*n* = 12)
Received human milk fortifier (+)	81.3% (*n* = 13)	64.3% (*n* = 9)
PEI health outcomes		
Necrotizing enterocolitis (NEC)	13% (*n* = 2)	0% (*n* = 0)
EOS	6.3% (*n* = 1)	0% (*n* = 0)
Late-onset sepsis (LOS)	44% (*n* = 7)	0% (*n* = 0)
Bronchopulmonary dysplasia (BPD)	63% (*n* = 10)	0% (*n* = 0)
Deceased	6% (*n* = 1)	0% (*n* = 0)

^*a*^ The gestational age of preterm infants ranged between 24 and 32 weeks. Still, it was further classified into subcategories of PB based on the World Health Organization standards ranging from extremely preterm (less than 28 weeks), very preterm (28 to less than 32 weeks), and moderate to late preterm (32 to 37 weeks) births. PEI stands for preterm infant.

### Sample processing, DNA extraction, and sequencing

Total DNA was extracted from 60 samples using the DNAesasy PowerLyzwer Power Soil Kit (Qiagen, Maryland, USA), followed by DNA quantification using dsDNA High Sensitivity Assay (Thermo Fisher Scientific, Massachusetts, USA) on the Qubit 4.0 platform to assess the DNA yield. After quantification, we barcoded 50 ng of genomic DNA per sample using the Native Barcoding Kit 96V14 (SQK-NBD 114.96) with a modification that involved a 10× increase in the volume of end-prepped DNA and other reagents for barcode ligation. Nuclease-free water was used as a negative control (background noise) during DNA extraction, library preparation, and sequencing. The final library was loaded onto an R10.4.1 (FLO-MIN106) flow cell and sequenced using a MinION device for 72 hours, followed by data analysis using the EPI2ME ARMA workflow for real-time antimicrobial resistance profiling based on the Comprehensive Antibiotic Resistance Database (CARD) AMR tool (18).

The nanopore raw signal was processed into reads in real time through Guppy. Barcodes were trimmed, and reads with a *q* score < 10 were filtered using Chopper before EPI2ME (19). A total of 2,581,644 reads were analyzed, with an average of 34,536 reads per sample, an average sequencing length of 4,174, and a quality score > 12. To determine the taxonomic composition of the microbial community, taxonomic profiling was conducted using the What’s In My Pot tool, which compares the sequenced reads against the NCBI RefSeq database (20). For microbiome analysis, taxonomic classification with <2,504 centrifuge score was removed (21, 22). Functional annotation was performed to identify potential ARGs by aligning the sequences against CARD with minimap2. Only clinically relevant ARGs with an alignment accuracy greater or equal to 87% were considered. ARG abundances were normalized based on the library size in average gene counts/giga base pair (gbp). This normalization method has been validated by other published literature (23, 24). According to the equation below, normalized ARG abundance at the species level is calculated by dividing the total count of ARGs (per species) by the total number of nucleotides in the data/1 gbp (23, 24). ARGs were categorized into subgroups based on antibiotic classes, adhering to the Center for Disease Control’s (CDC) definition of multidrug resistance, which involves resistance to at least one antibiotic in three or more drug classes.

Last, using the DIAMOND + MEGAN pipeline, sequences were aligned to the NCBI-nr database, followed by functional binning based on SEED classification.


$$\text{ ARG abundance (species)}=\frac{\text{ ARG counts }\text{ (per species) }}{\left[\text{ total no. of nucleotides }sequenced/1(Gbp)\right]}$$

### Statistical analysis

All statistical analyses were executed in R v4.3.1, with all survey data, including maternal health, neonatal treatment, and feeding practices, securely stored in REDCap, and exported as CSV files for processing (25, 26). The preterm infant resistome and microbiome diversity normality were tested using the Shapiro-Wilks test. Alpha diversity of the intestinal resistome was measured using the Shannon Index. The Shannon Index measures the diversity of a community by considering the number of species present and their relative abundance. The beta diversity of the infant resistome and microbiome was assessed using principal coordinates analysis with a Bray-Curtis dissimilarity matrix. Permutational Multivariate Analysis of Variance (PERMANOVA) implemented through the (Adonis) tests from the vegan package using distance matrices was conducted for each factor to determine significant differences in composition and metadata variables in the preterm infant resistome and microbiome (27, 28). Last, differential abundance of the preterm infant resistome was analyzed using the Wilcoxon signed-rank test. ARG abundance was transformed using the Centered Log Ratio (CLR) (29). This approach mitigates the inherent bias associated with varying total ARG counts across different samples by calibrating each ARG value against the geometric mean of all ARG abundances within the same sample. Similarly, functional output from MEGAN was analyzed after rarefaction to a minimum sampling depth of 1,000 sequences. The effect size was calculated using the wilcox_effsize function from the rstatix package (30).

## RESULTS

### Cohort description

Thirty infants (19 males and 11 females) across varying gestational ages, 23.3% (7) delivered extremely prematurely (<28 weeks’ gestation), 53.3% (16) delivered very prematurely (28 to <32 weeks), and 23.3% delivered moderately prematurely (32 to <37 weeks), were included in the study. Of the 30 neonates, 43% (13/30) were delivered vaginally, while 57% (17/30) were delivered via Cesarean section. The duration of ruptured membranes [rupture of membrane (ROM) length] ranged widely, from <1 hour to as long as 647 hours, with an average ROM length of 77 hours. Infant birth weight ranged between 525 and 2,230 g with a median of 1128 g.

Antibiotic usage by both mothers and neonates was diligently documented. In total, 67% (20/30) of mothers reported receiving antibiotics during pregnancy. Azithromycin was the most frequently administered antibiotic, followed by ampicillin and amoxicillin as standalone treatments. In addition, 50% of mothers who received antibiotics indicated receiving a combination of amoxicillin, gentamicin, and cefazolin at some point during their pregnancy. During delivery, 21/30 mothers (70%) received antibiotics. Once again, ampicillin was the most prescribed antibiotic, administered either as a standalone treatment (12/21 mothers) or combined with cefazolin (14/21 mothers).

The antibiotic regimen for preterm infants was meticulously tailored to individual health evaluations during NICU observations, resulting in a diverse range of antibiotics administered. However, all preterm infants receiving antibiotics were provided with broad-spectrum agents, including ampicillin alone or combined with gentamicin. This strategic combination effectively targeted a broad spectrum of Gram-positive and Gram-negative bacteria—aligning with the World Health Organization recommendations, which follow a standardized protocol for managing common childhood illnesses. According to this approach, prophylactic antibiotic therapy is initiated for at least 2 days, followed by reassessment in neonates with documented infection risks. Aminoglycosides, such as gentamicin- and penicillin-type antibiotics, are commonly employed as first-line antimicrobial therapy for treating EOS in neonatal settings (31). While ampicillin and gentamicin were the primary antibiotic agents, administering other antibiotics, including amoxicillin, vancomycin, cefotaxime, azithromycin, clindamycin, and oxacillin, was recorded. The duration of neonatal antibiotic administration varied on a case-by-case basis, spanning from 3 days to sequential courses lasting up to 12 days. Hospital stays ranged between 1 and 19 weeks post-delivery. Preterm infants who received antibiotics remained in the hospital 3× longer (median = 11, SD 4.48 weeks) than controls (median = 4, SD 2.3 weeks). Further details on patient demographics and health status can be found in Table 1.

### The intestinal resistome of preterm infants exhibited a rich diversity of ARGs

**Fig 3:**
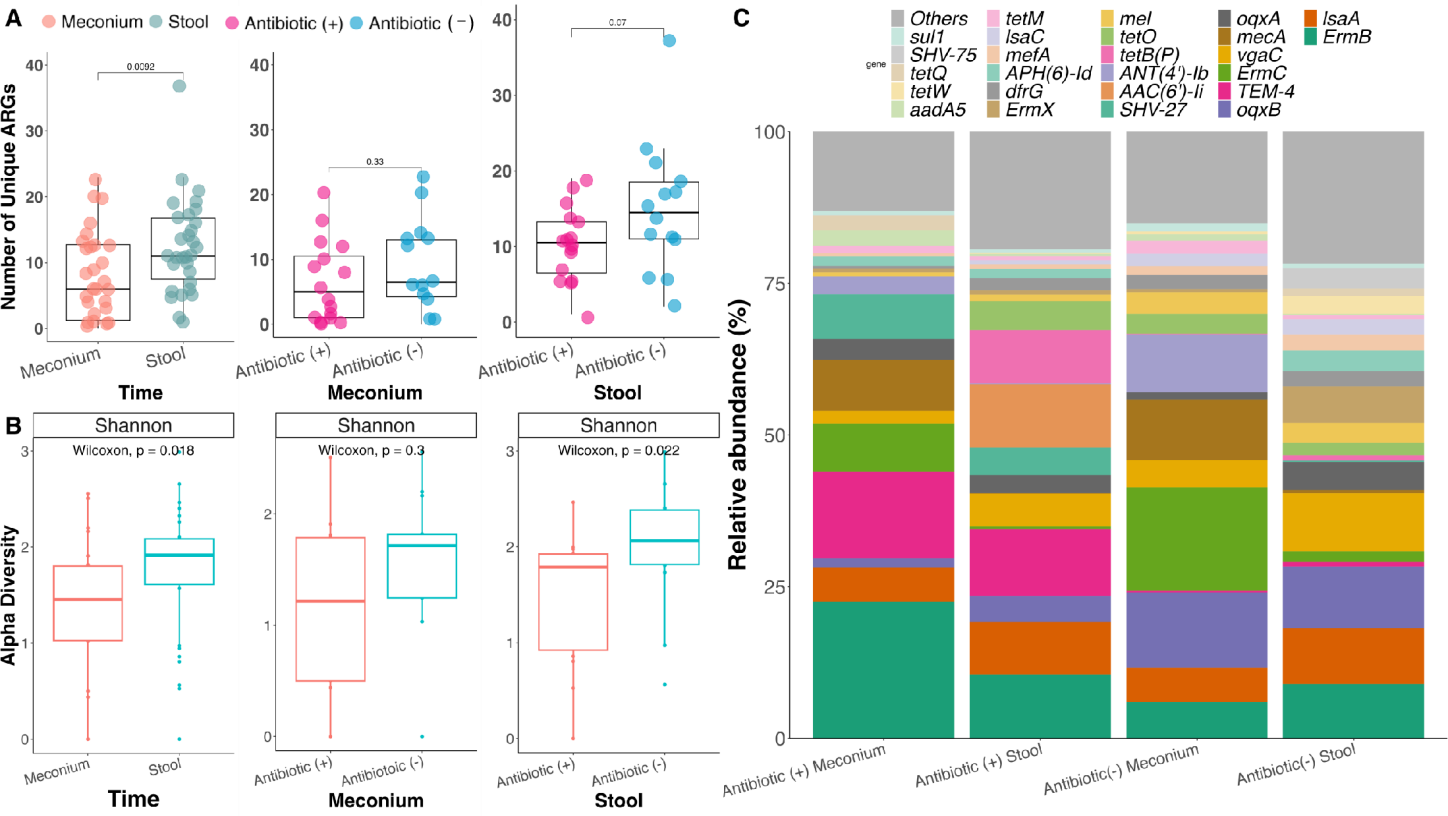
ARG diversity and abundance in preterm infant meconium and stool. (**A**) The number of unique ARGs identified in each infant based on time (meconium and stool) and treatment using the Wilcoxon signed-rank test. Antibiotic (+) represents those who received treatment compared with PEIs, who did not receive antibiotic (−). (**B**) Alpha diversity for the PEI resistome is depicted based on treatment and time. (**C**) Relative abundance of ARGs based on treatment and sample timeline. The top 25 most abundant ARGs are reported. PEI stands for preterm infant.

Throughout the study, a total of 175 distinct ARGs were detected. Among these, ARGs were identified in meconium samples from 26 preterm infants and in stool samples from all 30 preterm infants. On average, each positive sample contained approximately 11 ARGs, with a standard deviation of 7.4, indicating variability across samples. ARG detection increased in 79% of cases as the infants matured (*P* = 0.009, effect size = 0.337, and power = 99%) (Fig. 3A). While neonatal antibiotic treatment did not impact the number of ARGs detected in meconium samples, it moderately affected ARG detection in stool samples (*P* = 0.069, effect size = 0.335, and power = 62%) (Fig. 3A).

The 175 ARGs identified corresponded to 15 different drug classes. Among them, beta-lactams 62.3% (109/175 ARGs), tetracycline 9.7% (17/175 ARGs), and aminoglycosides 6.3% (11/175 ARGs) were the most prevalent, followed by macrolides-lincosamides-sulfides (MLS) 5.1% (9/175 ARGs) (Table S1). In meconium, ARGs associated with all 15 drug classes were detected, of which beta-lactams (67/109 ARGs), tetracycline (10/17 ARGs), and aminoglycosides (5/11 ARGs) were predominant.

Resistance to fusidane and phosphonic acids was exclusively detected in meconium, involving 3 out of 26 infants, indicating that ARGs were found for 13 drug classes in stool samples. The analysis revealed no significant temporal differences in the number of unique ARGs, except for tetracycline, which exhibited an increase in 99% of preterm infants over time (*P* = 0.023, effect size = 0.89, and power = 100%) (Fig. S1). No statistical differences between neonatal antibiotic treatment and drug resistance were identified using meconium samples (Fig. S2). However, infants who did not receive antibiotics had more ARGs related to diaminopyrimidine than neonates who received antibiotics (*P* = 0.031, effect size = 0.68, and power = 99.5%) (Fig. S3). Last, 23.4% (41/175) of ARGs persisted regardless of neonatal or intrapartum antibiotic exposure; again, beta-lactam-associated ARG was most common at 51.2% (21/41), followed by MLS at 12.3% (5/41) and tetracycline at 9.8% (4/41). When classified under their respective resistance mechanisms (i.e., antibiotic inactivation, target alteration, antibiotic target protection, target replacement, or efflux), genes associated with antibiotic inactivation commonly encode for enzymes responsible for modifying or degrading antibiotics, had the highest prevalence over time, and were observed regardless of the neonatal antibiotic treatment regimen (Fig. S4).

Changes in drug resistance profiles, as measured by the diversity of ARGs, were observed over time. Furthermore, the resistome exhibited an increasing trend in ARG diversity (*P* = 0.022, effect size = 0.32, and power = 81%). While neonatal antibiotic treatment did not influence the diversity of the meconium resistome, it did affect the diversity of the stool resistome. Preterm infants not directly exposed to antibiotics exhibited a greater ARG diversity than those who received antibiotics (*P* = 0.022, effect size = 0.42, and power = 79%) (Fig. 3B). Additionally, 33% of infants born from mothers with ruptured membranes (ROM) lasting more than 18 hours exhibits a higher ARG diversity in the stool resistome compared with infants with a shorter ROM duration (*P* = 0.035, effect size = 0.39, and power = 76%) (Fig. S6). Specifically, 99% of these infants from mothers with prolonged ROM exhibited an increased quantity of unique ARGs corresponding to beta-lactam resistance, indicating a significant difference (*P* = 0.0079, effect size = 1.28, and power = 100%) (Fig. S8). Although maternal GBS colonization did not statistically impact the infant meconium or stool resistome, this may be attributed to the fact that 30% of infants in our study had mothers with unknown GBS status at the time of delivery. Notably, 71% of infants whose mothers’ GBS status was unknown exhibited greater ARG diversity in meconium compared with GBS-negative cases (*P* = 0.056, effect size = 0.396, and power = 55%). This finding underscores the critical need for early GBS screening measures. The absence of early GBS screening and the presence of maternal symptoms often lead to the use of prophylactic antibiotics, with 78% (*n* = 7/9) of mothers with unknown GBS status receiving intrapartum antibiotics. ARG diversity was not influenced by delivery mode, infant sex, clinical diagnosis of chorioamnionitis, or maternal antibiotic utilization after birth or before hospital discharge (Fig. S5 to S15).

### Maternal and neonatal antibiotic utilization shapes the preterm infant resistome over time

PERMANOVA analysis was conducted to understand the factors affecting the ARG beta diversity of the preterm infant meconium and stool resistome. Multivariate analysis incorporating maternal antibiotic use, delivery method, neonate treatment, and feeding practices did not identify these factors as significant contributors to variations in the meconium resistome (Table 2). The *R²* values from pairwise comparisons indicated that antibiotic use during gestation accounted for 7% (*R²* = 0.066, *P* = 0.018) and intrapartum antibiotic use explained 6% (*R²* = 0.059, p = 0.041) of the variations in the meconium resistome. Specifically, ampicillin use during delivery accounted for 8% (*R*^2^ = 0.082, *P* = 0.006) of this variance (Table S2).

**TABLE 2:** PERMANOVA analysis of the meconium resistome and microbiome of preterm neonates^*a*^

Factors	Variable	Resistome			Microbiome		
		*R* ^2^	*F*	*P* value	*R* ^2^	*F*	*P* value
ANC	Prenatal antibiotics (yes/no)	0.03412	0.8011	0.726	0.05224	1.8826	0.072*
	No. of prenatal antibiotics administered	0.04475	1.0509	0.464	0.04403	1.5869	0.112
Delivery	No. of intrapartum antibiotics administered	0.04089	0.9602	0.571	0.02562	0.9234	0.539
	Intrapartum antibiotics (yes/no)	0.06624	1.5554	0.116	0.0641	2.3102	0.021**
	Chlamydia	0.05093	1.1959	0.326	0.04042	1.4567	0.157
	GBS	0.09961	1.1695	0.322	0.08173	1.4728	0.118
	Magnesium sulfate	0.04378	1.0281	0.471	0.03176	1.1445	0.348
	ROM length ≥ 18 hours	0.03861	0.9066	0.607	0.04991	1.7989	0.09*
	Delivery mode	0.03772	0.8857	0.62	0.03437	1.2387	0.28
	Gestational age	0.03724	0.8745	0.649	0.01371	0.4943	0.924
	Infant birth weight	0.05381	1.2636	0.259	0.08096	2.9178	0.005**
PEI treatment	Infant sex	0.04792	1.1253	0.371	0.03462	1.2476	0.252
	Group (antibiotic: yes/no)	0.04702	1.104	0.404	0.02939	1.0592	0.388
	Ampicillin and gentamicin administration	0.01824	0.4283	0.987	0.03053	1.1003	0.382
	No. of antibiotics administered	0.03387	0.7953	0.734	0.03861	1.3915	0.19
	Average duration of antibiotics	0.03294	0.7735	0.772	0.03366	1.2133	0.278
	Length of hospital stay	0.03178	0.7462	0.786	0.03001	1.0816	0.367
PEI feeding	No enteral feeding	0.04751	1.1156	0.388	0.02115	0.7623	0.701
	Fed mother and donor breastmilk (yes/no)	0.02935	0.6892	0.843	0.03992	1.4389	0.165
	Fed mother breastmilk (yes/ no)	0.03232	0.7589	0.764	0.01938	0.6983	0.775
	Fed donor breastmilk (yes/no)	0.03549	0.8334	0.707	0.01955	0.7046	0.736
	Fed formula (yes/no)	0.02822	0.6627	0.866	0.01401	0.5048	0.933
	Received human milk fortifier (yes/no)	0.02506	0.5885	0.922	0.03157	1.1378	0.322

^*a*^ PERMANOVA, permutational multivariate analysis of variance; ARGSs, antibiotic resistance. Variables with a statistically significant *P* value have been annotated in bold and an * and a marginally significant *P* value in bold. Findings from 26 meconium samples are included in this table. The infant resistome and microbiome detected 175 unique ARGs and 774 bacterial species. Variables demonstrating a statistically significant *P* value have been denoted with **, while marginally significant *P* values are marked with *. PEI stands for preterm infant.

In contrast to the meconium resistome, the number of antibiotics utilized by the mother during gestation (*R*^2^ = 0.044, *P* = 0.054), infant birth weight (*R*^2^ = 0.043, *P* = 0. 041), and neonate antibiotic utilization (*R*^2^ = 0.055, *P* = 0. 016) contributed to roughly 5% of variations in the stool resistome, with marginal effect attributed to ampicillin and gentamicin administration to neonates (*R*^2^ = 0.041, *P* = 0.074) (Table 3). The administration of beta-lactams to preterm infants influenced the stool resistome, of which ampicillin and ceftazidime attributed to 5%–6% of the variations in the resistome (ampicillin—*R*^2^ = 0.052, *P* = 0.046, ceftazidime—*R*^2^ = 0.063, *P* = 0.0006) (Table S3).

**TABLE 3:** PERMANOVA analysis of the stool resistome and microbiome of preterm neonates^*a*^

Factors	Variable	Resistome			Microbiome		
		*R* ^2^	*F*	*P* value	*R* ^2^	*F*	*P* value
ANC	Prenatal antibiotics (yes/no)	0.03667	1.3046	0.151	0.04574	1.7035	0.078*
	No. of prenatal antibiotics administered	0.04355	1.5496	0.054**	0.05043	1.8782	0.054**
Delivery	No. of intrapartum antibiotics administered	0.0372	1.3237	0.143	0.04229	1.5749	0.115
	Intrapartum antibiotics (yes/no)	0.03404	1.211	0.232	0.04212	1.5688	0.105
	Chlamydia	0.02987	1.0626	0.39	0.03385	1.2607	0.279
	GBS	0.07365	1.3102	0.115	0.05195	0.9674	0.504
	Magnesium sulfate	0.03817	1.3579	0.106	0.04006	1.4921	0.119
	ROM length ≥ 18 hours	0.03748	1.3335	0.118	0.04751	1.7694	0.094*
	Delivery mode	0.03764	1.3393	0.141	0.03039	1.1319	0.347
	Gestational age	0.03916	1.3934	0.103	0.02226	0.829	0.618
	Infant birth weight	0.04277	1.5217	0.041*	0.04519	1.683	0.088*
PEI treatment	Infant sex	0.03844	1.3675	0.131	0.04113	1.5317	0.13
	Group (antibiotic: yes/no)	0.05463	1.9435	0.016**	0.05739	2.1373	0.03**
	Ampicillin and gentamicin administration	0.04074	1.4494	0.074*	0.03507	1.306	0.225
	No. of antibiotics administered	0.03433	1.2216	0.246	0.02513	0.9361	0.513
	Average duration of antibiotics	0.03423	1.2177	0.218	0.03374	1.2567	0.232
	Length of hospital stay	0.02716	0.9662	0.486	0.02559	0.9529	0.472
PEI feeding	No enteral feeding	0.03474	1.2358	0.201	0.02449	0.9121	0.552
	Fed mother and donor breastmilk (yes/no)	0.03003	1.0684	0.407	0.02622	0.9764	0.491
	Fed mother breastmilk (yes/no)	0.0219	0.7791	0.765	0.01521	0.5665	0.879
	Fed donor breastmilk (yes/no)	0.02647	0.9418	0.579	0.02299	0.8562	0.574
	Fed formula (yes/no)	0.03223	1.1468	0.292	0.05046	1.8794	0.06*
	Received human milk fortifier (yes/no)	0.03437	1.2227	0.209	0.05652	2.105	0.029**

^*a*^ PERMANOVA, permutational multivariate analysis of variance; ARGSs, antibiotic resistance. Variables with a statistically significant *P* value have been annotated in bold and an * and marginally significant *P* value in bold. Findings from 30 stool samples are included in this table. The infant resistome and microbiome detected 175 unique ARGs and 774 bacterial species. Variables demonstrating a statistically significant *P* value have been denoted with **, while marginally significant *P* values are marked with *. PEI stands for preterm infant.

Last, the impact of the meconium and stool infant resistome on adverse health outcomes was investigated. Although no direct link was established between the neonate meconium resistome and adverse clinical outcomes, EOS and bronchopulmonary dysplasia (BPD) status contributed to 4%–6% of the variability in the preterm infant stool resistome (EOS—*R*^2^ = 0.046, *P* = 0.032 and BPD—*R*^2^ = 0.063, *P* = 0.004) (Table 4). This suggests that adverse health events early in life may exert a prolonged influence on the resistome as infants age.

**TABLE 4:** PERMANOVA analysis of the preterm infant meconium/stool resistome and microbiome on adverse neonatal outcomes^*a*^^,*b*^

Variables	Resistome			Microbiome		
	*R* ^2^	*F*	*P* value	*R* ^2^	*F*	*P* value
PEI meconium						
NEC	0.03759	0.9459	0.553	0.03193	0.9533	0.524
EOS	0.03889	0.9785	0.521	0.04624	1.3806	0.058*
LOS	0.04692	1.1806	0.221	0.0483	1.4421	0.136
BPD	0.04206	1.0584	0.369	0.03217	0.9604	0.463
Death	0.03971	0.9991	0.57	0.03748	1.119	0.409
PEI stool						
NEC	0.04292	1.3308	0.094*	0.03577	1.14	0.354
EOS	0.04602	1.4267	0.032**	0.05791	1.8456	0.009**
LOS	0.03524	1.0926	0.326	0.01675	0.5339	0.929
BPD	0.06277	1.9462	0.004**	0.08642	2.7542	0.005**
Death	0.03894	1.2072	0.197	0.05007	1.5956	0.055*

^*a*^ This PERMANOVA assesses the infant resistome and intestinal microbiome and their relationship with neonatal health outcomes. Variables demonstrating a statistically significant *P* value have been denoted with **, while marginally significant *P* values are marked with *. Time was considered a confounding factor in this analysis. PEI stands for preterm infant.

^*b*^ BPD is characterized by an ongoing need for supplemental oxygen and respiratory support in preterm neonates (<32 weeks gestational age) at either 28 days post-natal age or 36 weeks post-menstrual age, accompanied by radiographic evidence of parenchymal lung disease. Infants diagnosed with BPD had this diagnosis made late in their hospital stay, before discharge, and most stool samples collected were before the diagnosis of BPD.

### Early exposure to antibiotics increases the beta-lactam and tetracycline resistance

In this study, the top five most abundant drug resistance classes identified were beta-lactam, MLS, LS, tetracycline, and aminoglycosides. Beta-lactam, MLS, LS, and tetracycline were consistently the most abundant in meconium and stool samples. However, aminoglycosides were found to be more abundant in meconium samples, while multidrug resistance was more abundant in stool samples.

**Fig 4:**
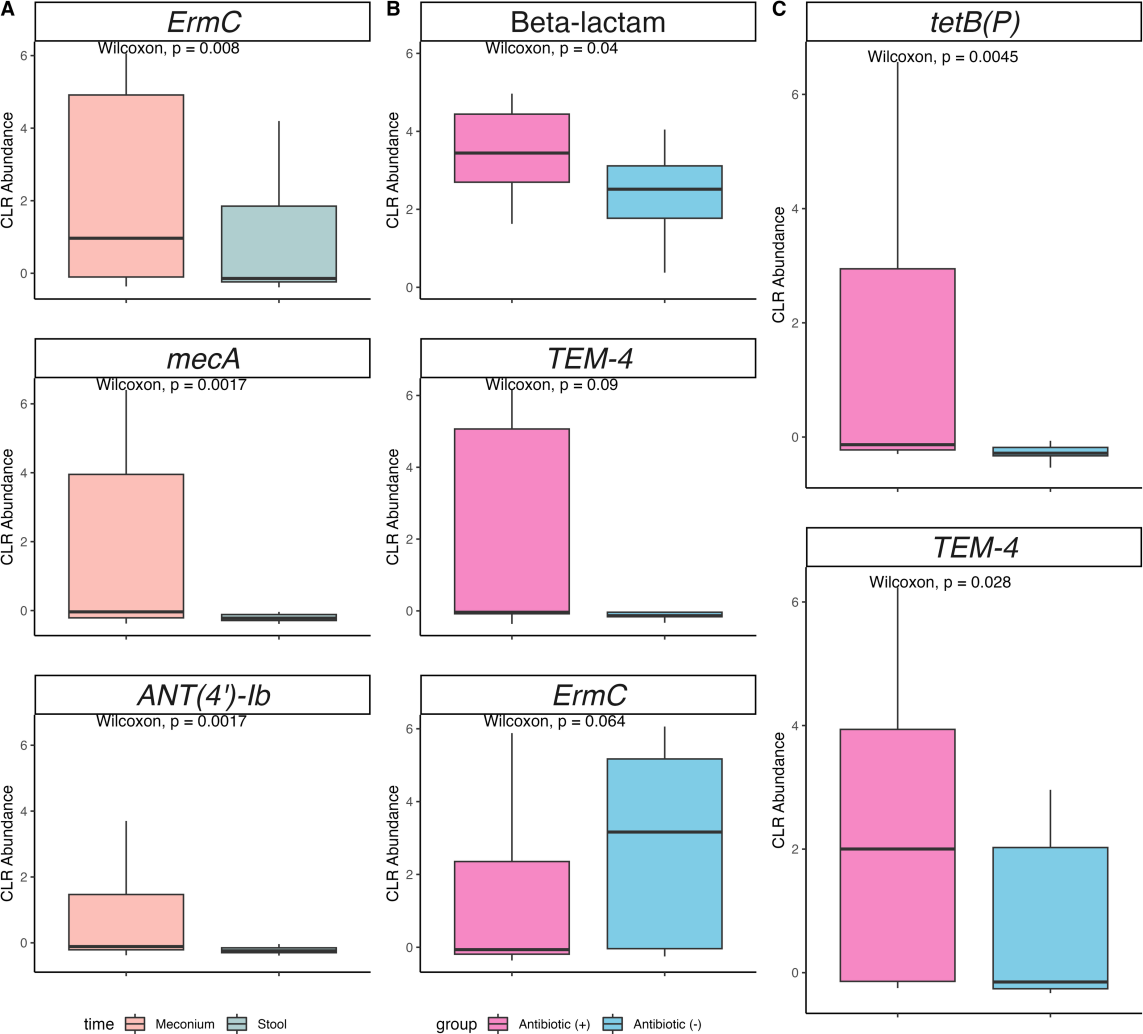
Variance in preterm infant resistance abundance at the ARG level. (**A**) Comparison of ARGs over time using meconium and stool samples. Panels **B** and **C** display the differential abundance of ARGs based on neonatal antibiotic treatment in meconium (*n* = 26 samples/PEIs) and stool samples (*n* = 30 samples/PEIs), respectively. PEI stands for preterm infant.

At the ARG level, ARGs pertaining to MLS (*ErmC* and *ErmB*), beta-lactams (*TEM-4*), LS (*IsaA* and *vgaC*), and oqxB (multidrug) resistance were the most abundant throughout the study (Fig. 3C; SM 16). In 82%–85% of the samples, ARGs associated with MLS resistance encoded by *ErmC* (*P* = 0.008, effect size = 0.36, and power = 99%), *mecA* (encoding beta-lactams) (*P* = 0.0017, effect size = 0.42, and power = 99%), and *ANT(4′)-Ib* encoding for aminoglycosides (*P* = 0.0017, effect size = 0.42, and power = 99%) were more abundant in meconium versus stool samples (Fig. 4A; ST 4). In meconium samples, neonatal antibiotic treatment displayed a moderate effect on ARG abundance, as preterm infants who did not receive antibiotic treatment had a greater abundance of *ErmC* (*P* = 0.064, effect size = 0.37, and power = 70%).

Seventy-two percent of preterm infants who received antibiotics had a greater abundance of genes with resistance to the beta-lactam class (*P* = 0.04, effect size = 0.32, and power = 58%), of which the *TEM-4* ARG was the most abundant (*P* = 0.09, effect size = 0.32, and power = 63%) (Fig. 4B; ST 5). The *TEM-4* gene persisted in being more abundant and significantly higher in stool samples from preterm infants who received antibiotics (*P* = 0.028, effect size = 0.40, and power = 77%). Although the abundance of tetracycline resistance genes remained relatively low across both time points, preterm infants treated with antibiotics had a greater abundance of *tetB(P)* as they got older (*P* = 0.0045, effect size = 0.51, and power = 92%) (Fig. 4C; ST 6).

**Fig 5:**
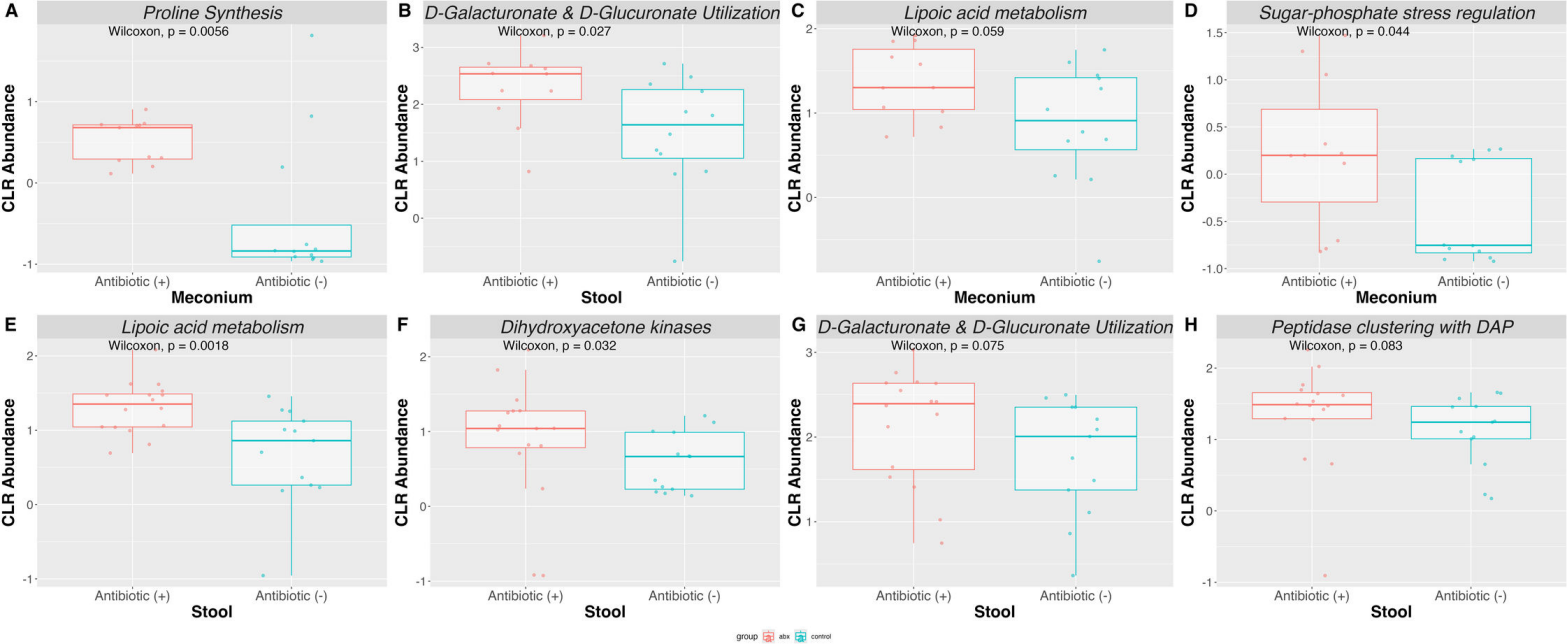
Functional profiling of infant gut post-antibiotic exposure. Functional analysis using output from MEGAN. Differential abundance was performed using meconium (**A–D**) and stool samples (**E–H**) (*n* = 60 samples total).

Even though only two ARGs associated with multidrug resistance (oqxA and oqxB) were detected, it was identified in 97% (*n* = 29/30) of preterm infants and increased in prevalence and abundance as the infant aged (Fig. 5). Although oqxB was more abundant in preterm infants who did not receive antibiotic treatment (Fig. 5A B), this difference was not statistically significant. While the duration of ROM significantly influenced the abundance of numerous ARGs across different drug classes in stool samples, it was observed that 76% of preterm infants born to mothers with prolonged ROM exhibited a higher abundance of multidrug resistance (*P* = 0.023, effect size = 0.42, and power = 80%) (ST 7). Specifically, the gene *oqxB* was more abundant in this group in 80% of preterm infants (*P* = 0.0038, effect size = 0.51, and power = 93%).

### Metabolic shifts enhance antibiotic tolerance

Based on functional annotation, we found that in meconium, 79% of infants who received antibiotic treatment exhibited a greater abundance of cyclic di-GMP turnover proteins (*P* = 0.03, effect size = 0.43, and power = 84%). Cyclic di-GMP is a critical bacterial messenger that regulates various cellular processes, significantly influencing adherence and developing mature biofilm structures (32, 33). Furthermore, cyclic di-GMP turnover proteins maintain the balanced cyclic di-GMP levels within the cell, influencing bacterial behavior and adaptation to environmental changes. Additionally, several metabolic pathways were affected by antibiotic treatment. Specifically, 70%–85% of antibiotic-treated infants showed increased proline synthesis (*P* = 0.0056, effect size = 0.56, and power = 83%), known for its dual role as an osmoprotectant under osmotic stress and in supporting efflux pump activities (34). This was followed by increased D-galacturonate and D-glucuronate utilization (*P* = 0.027, effect size = 0.46, and power = 67%), lipoic acid metabolism (*P* = 0.059, effect size = 0.39, and power = 55%), and sugar-phosphate stress regulation (*P* = 0.044, effect size = 0.42, and power = 60%) (Fig. 5A through D; ST8). These findings indicate that antibiotic treatment leads to significant metabolic alterations, enhancing bacterial survival in unfavorable environmental conditions.

This trend persisted as infants aged, with antibiotic-treated infants having a greater abundance of lipoic acid metabolism (*P* = 0.0018, effect size = 0.39, and power = 75%), D-galacturonate and D-glucuronate utilization (*P* = 0.075, effect size = 0.46, and power = 87%), and peptidase clustering with diaminopimelic acid (DAP) (*P* = 0.083, effect size = 0.44, and power = 84%), which play a role in facilitating peptidoglycan synthesis (35). In addition, changes in energy generation were observed in 79% of antibiotic-treated infants, who exhibited a significantly higher abundance of dihydroxyacetone kinases (*P* = 0.032, effect size = 0.31, and power = 71%) compared with untreated infants (Fig. 5E through H; ST9). Dihydroxyacetone kinases play a crucial role in metabolism by phosphorylating dihydroxyacetone, which integrates into metabolic pathways such as glycolysis and contributes to biofilm formation (36). These results suggest that early antibiotic exposure in preterm neonates selects for gut organisms with altered metabolic pathways. This further supports that gut-associated bacteria employ sophisticated survival strategies, underscoring the importance of metabolic flexibility in bacterial survival.

### The interplay between the preterm infant resistome and the microbiota

A total of 774 species were detected across all 60 samples. Among these species, those belonging to the genera *Streptococcus* (44 species), *Staphylococcus* (31 species), *Corynebacterium* (29 species), and *Clostridium* (27 species) exhibited the highest ARG diversity, and *Escherichia coli*, *Klebsiella pneumoniae*, *Enterococcus faecalis*, *Staphylococcus epidermidis*, and *Fusobacterium nucleatum* were the most abundant.

Of the detected species, 40 harbored ARGs, with 60% (*n* = 24/40) belonging to the *Proteobacteria* phylum. Of the 15 different drug resistance classes detected in this study, the species richness was high concerning three drug classes, including MLS (*n* = 17/40), beta-lactam (*n* = 14/40), and tetracycline (*n* = 11/40), followed by aminoglycoside (*n* = 7/40) resistance. *E. coli* and *Staphylococcus aureus* carried the most ARGs related to different drug classes, with *K. pneumonia* (*n* = 60/175) and *E. coli* (*n* = 41/175) harboring the highest number of ARGs, with the majority conferring to beta-lactam resistance.

**Fig 6:**
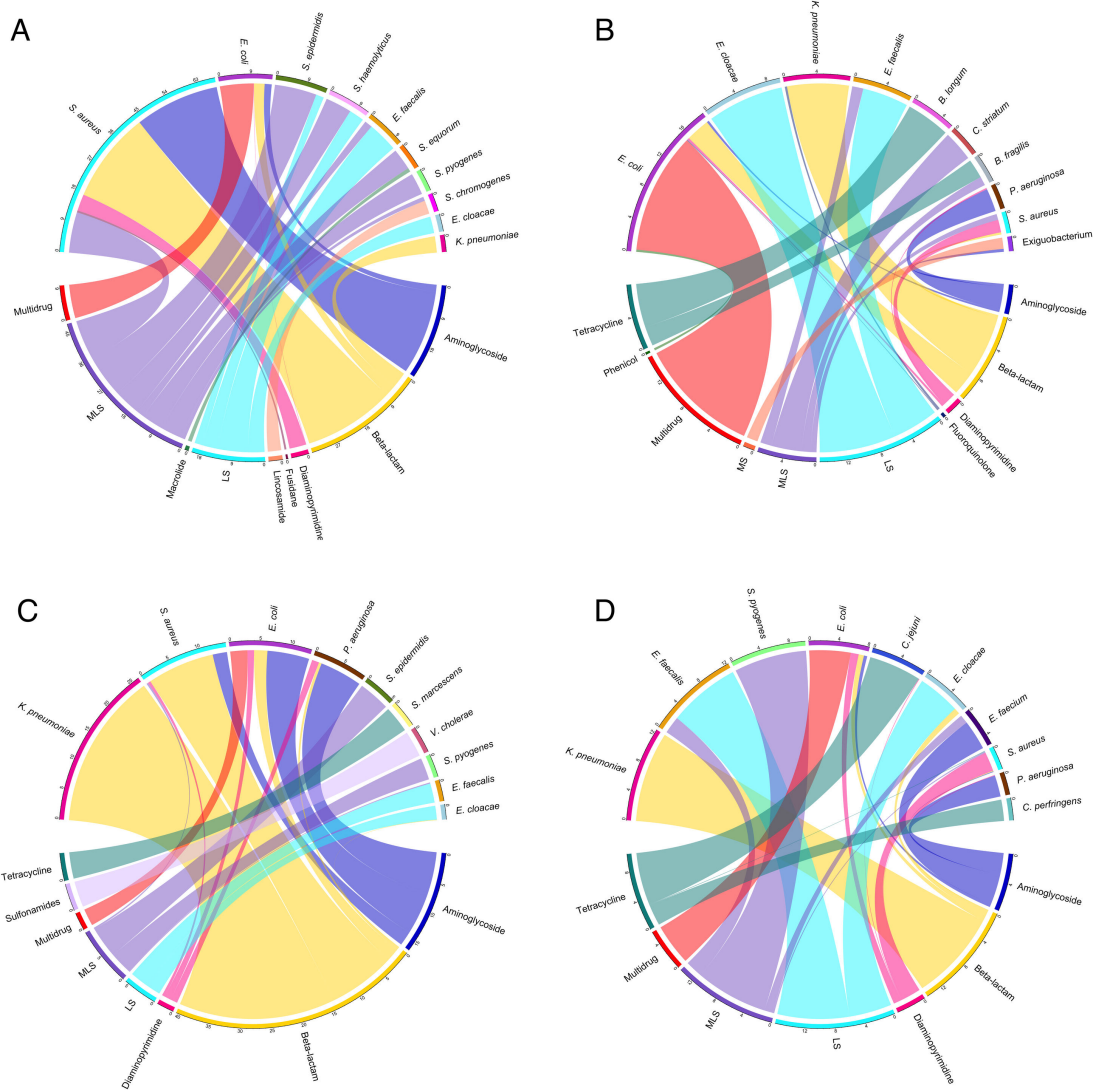
Comparative chord diagrams of bacterial species and ARG abundance in neonatal meconium and stool samples. This figure presents two pairs of chord diagrams, each illustrating the interplay between the top 50 most prevalent ARGs categorized by drug class and the top 10 bacterial species associated with the ARG. Diagrams **A** and **B** represent preterm infants who did not receive direct exposure to antibiotics, whereas diagrams **C** and **D** correspond to the antibiotic-treated group. Specifically, diagrams A and C highlight the relationships within meconium samples, and diagrams B and D show the connections within stool samples collected just before hospital discharge.

In this study, a group of pathogens called ESKAPE (comprised of *Enterococcus faecium*, *S. aureus*, *K. pneumoniae*, *Acinetobacter baumannii*, *Pseudomonas aeruginosa*, and *Enterobacter* species) was identified and linked to at least one ARG. From the ESKAPE group, *S. aureus* (21 ARGs/gbp), *K. pneumoniae* (18 ARGs /gbp), *Enterobacter cloacae* (14 ARGs/gbp), and *P. aeruginosa* (13 ARGs/gbp) exhibited the highest abundance of ARGs. *S. aureus* harbored the most diverse ARGs conferring to beta-lactam (including mecA-encoding methicillin), diaminopyrimidine, MLS, aminoglycoside, fusidane, and tetracycline resistance, followed by *P. aeruginosa*, also linked to diaminopyrimidine, beta-lactam, and aminoglycoside resistance, of which aminoglycoside (*APH(6)-Id* and *APH(3′’-Ib)* was the most abundant (Fig. 6). Furthermore, several non-ESKAPE species were also found to carry ARGs, including *E. coli*, *E. faecalis*, *Streptococcus pyogenes*, and *Campylobacter jejuni*. Interestingly, only *E. coli* carried ARGs associated with multidrug resistance.

Last, ARG diversity in the resistome is also associated with shifts in microbiota diversity. Moreover, *S. aureus* carrying ARGs were more prevalent (*P* = 0.033, effect size = 0.55, and power = 96%) and abundant (*P* = 0.0016, effect size = 0.57, and power = 97%) in meconium samples, of which *Staphylococcus* species carrying ARGs were more prevalent from preterm infants who did not receive direct antibiotic exposure (*P* = 0.024, effect size = 0.53, and power = 95%), while preterm infants who received antibiotics had more *P. aeruginosa* harboring ARGs (*P* = 0.11, effect size = 0.67, and power = 58%) (Fig. 6A and C). In stool samples, preterm infants who did not receive antibiotics had more *S. aureus* harboring ARGs than those treated with antibiotics (*P* = 0.033, effect size = 0.53, and power = 40%).

### Antibiotic use, feeding practices, and time drive microbiota dynamics

Beta diversity of the meconium microbiota was influenced by several factors, in particular, birth weight, which explained the most significant variation of 8% (*R*^2^ = 0.081, *P* = 0.021), followed by antibiotics administered during delivery (*R*^2^ = 0.064, *P* = 0.021) of which administration of ampicillin attributed 7% (*R*^2^ = 0.066, *P* = 0.054) variations to the microbiota (Table 2; Table S2). Furthermore, antibiotic utilization during gestation (*R*^2^ = 0.052, *P* = 0.072), specifically utilization of amoxicillin (*R*^2^ = 0.067, *P* = 0.056), and prolonged duration of ruptured membranes (*R*^2^ = 0.049, *P* = 0.09) contributed to marginal effects on the meconium microbiota (Table 2; Table S2).

Neonatal antibiotic treatment and the intake of human milk fortifier were responsible for approximately 6% of the variability observed in the stool microbiota (*R*^2^ = 0.057, *P* = 0.03 and *R*^2^ = 0.056, *P* = 0.029) (Table 3). Consistent with observations in the stool resistome, the microbiota was also influenced by the administration of beta-lactams, primarily ceftazidime, which explained 8% of the observed variations (*R*^2^ = 0.080, *P* = 0.003), followed by minor variability attributed to ampicillin and piperacillin (*R*^2^ = 0.052, *P* = 0.079 and *R*^2^ = 0.053, *P* = 0.069) (Table S3). Furthermore, antibiotic exposure during gestation (*R*^2^ = 0.050, *P* = 0.054) and birth weight exhibited a marginal impact on the microbiota as the infant aged (*R*^2^ = 0.045, *P* = 0.088). Additionally, prenatal antibiotic exposure (*R*^2^ = 0.046, *P* = 0.078) and prolonged rupture of membranes (*R*^2^ = 0.048, *P* = 0.094) continued to exert a marginal influence on the microbiota as it evolved (Table 3).

Regarding clinical implications, the variability in the meconium microbiota was associated with early-onset sepsis (*R*^2^ = 0.046, *P* = 0.058). The incidence of EOS continued to drive the variability in the microbiota as the infant aged (*R*^2^ = 0.058, *P* = 0. 009), highlighting the critical influence of the microbiota on neonatal health outcomes. Moreover, BPD status emerged as a significant factor, explaining 9% of the variability in the stool microbiota (*R*^2^ = 0.086, *P* = 0.005) (Table 4). These findings emphasize the impact of antibiotic use and neonatal nutrition on the microbiota and illustrate the microbiome’s significant influence on key neonatal health challenges, including sepsis and BPD.

## DISCUSSION

The infant gut serves as a critical reservoir for ARGs (37, 38). Our study reveals that the intestines of premature infants harbor a high prevalence and an array of ARGs, a trend that persists irrespective of direct antibiotic exposure and intensifies with the infant’s age. This pattern aligns with recent research by Leo et al. (1) and Guitor et al. (15), which detected ARGs in full-term and preterm neonates 7–10 days post-partum who had not been administered antibiotics. In addition, our results suggest that antibiotic use 48 hours after birth did not affect the quantity or diversity of ARGs in meconium but did have a lasting effect on the stool resistome. Preterm infants not exposed to antibiotics had higher quantities and diversity of ARGs in stool samples, suggesting that ARG enrichment is a natural aspect of the evolving gut microbiota, correlating with increased species richness—a trend mirrored in the growing number of species carrying ARGs as the infants mature.

Early antibiotic exposure contributed to heightened beta-lactam resistance, which was more prevalent in meconium from antibiotic-treated infants, as beta-lactams were the most prescribed antibiotic within 48 hours post-delivery. Beyond genomic analysis, we found that the gut microbiota withstands antibiotic pressure by altering several pathways, including cell signaling, energy production, and metabolic reprogramming. For instance, an increase in cyclic di-GMP turnover proteins and dihydroxyacetone kinase levels in antibiotic-treated infants indicates a transition toward biofilm formation, a known survival strategy. Additionally, antibiotic-treated infants exhibited significant metabolic shifts, such as increased proline synthesis, lipoic acid metabolism, and D-galacturonate/D-glucuronate utilization. The persistence of these metabolic changes as infants age underscores the long-term impact of early antibiotic exposure on the developing gut microbiome. Collectively, these modifications enhance bacterial resilience under stress by slowing growth, thereby aiding in antibiotic tolerance—compromising the efficacy of bactericidal drugs like beta-lactams (39–41). This illustrates a complex and multifaceted bacterial defense mechanism that may diminish the effectiveness of antibiotic treatments, posing a challenge for clinical management and highlighting the critical need for judicious antibiotic use.

Despite significant advancements in antenatal care and antimicrobial stewardship, sepsis ranks among the top 10 causes of neonatal mortality in the United States (9). In response to this threat, administering antibiotics as an empiric therapy has become widespread in neonatal care. Considering such prevalent antibiotic use in this setting, the REASON study is the first trial to randomize neonates to receive or not receive antibiotics during the first 48 hours after birth to evaluate the impact of antibiotic treatment on the developing gut microbiome and metabolome (13, 42). Thus, this study aimed to assess whether the routine use of antibiotics in preterm infants augments ARG detection within the resistome and evaluate how it alters the premature infant microbiome.

ARGs were detected across 15 drug classes in the infant meconium, persisting as neonates aged. Corroborating with other studies, the most detected ARGs conferred resistance to beta-lactam, tetracycline, aminoglycosides, and macrolides. Beta-lactam, aminoglycosides, and macrolide resistance could be attributed to the antibiotics administered to neonates directly or to the mother during gestation or at birth, as they were the most frequently prescribed antibiotics during our study. Three species harboring beta-lactam resistance were identified: *K. pneumoniae*, *S. aureus*, and *E. coli*. Notably, *K. pneumoniae* was more prevalent in antibiotic-treated infants, while *S. aureus* predominated beta-lactam resistance in preterm infants unexposed to antibiotics. However, the identification of ARGs conferring resistance to other drug classes, such as tetracycline, diaminopyrimidine, and multidrug resistance, could be driven by maternal antibiotic utilization earlier in life, which was outside the scope of our study.

Nonetheless, tetracycline resistance *tet* genes have been reported to be the most frequently shared ARGs between mothers and newborn infants (43, 44), . Although tetracycline is not recommended during pregnancy and early life due to its potential dental staining effects, it is often used to treat infections and used in animals, resulting in environmental and dietary sources for *tet* genes in women, which can be found in infants without direct exposure to the antibiotic (17). In our study, tetracycline resistance genes were more abundant in meconium from antibiotic-treated infants, increased over time in both infant groups, and were detected in multiple species. Beyond vertical transmission, it is critical to acknowledge the role of environmental factors in the emergence of ARGs. The rise of antibiotic-resistant bacteria poses a significant risk in NICUs, particularly affecting preterm infants who typically have longer hospital stays than their full-term counterparts (45). This extended hospitalization period for preterm infants may increase their vulnerability to encountering and harboring antibiotic-resistant bacteria.

Apart from the direct administration of antibiotics to newborns, our study highlights how maternal antibiotic usage and health status significantly influence the microbiota and the resistome of preterm infants. We found that antibiotic use by mothers during pregnancy and childbirth accounts for 6%–7% of the variance in the diversity of microbial species and ARG profiles. Specifically, administering an ampicillin regimen during delivery, a strategy commonly aimed at reducing GBS colonization in newborns, attributed to 6%–8% of the variance in the meconium resistome and microbiome, with enduring effects in the stool resistome.

GBS is a prominent cause of infection in newborns, with virulence factors like adhesins and hemolytic activities that enhance its survival and ability to colonize various sites, occasionally even affecting the respiratory tract (46). Current guidelines recommend screening for GBS between 36 and 37 weeks of gestation. However, this strategy may not adequately account for preterm deliveries, often resulting in mothers missing the critical screening. Consequently, in scenarios where GBS status is indeterminate—particularly when there’s no maternal fever or when a prolonged ROM extends beyond 18 hours—beta-lactam antibiotics, like penicillin and ampicillin, are administered during labor as a preventative measure against GBS, which is known to lead to both early-onset and later-life neonatal infections (47, 48). Intriguingly, research by Roesch et al. (48) involving 27 mothers who received intrapartum antibiotics discovered an increased microbial diversity and lower species dominance in their vaginal microbiota relative to those who did not receive treatment. This heightened diversity could expose neonates to a broader range of bacteria, including those harboring ARGs. Of the neonates in this study, 33.3% (10/30 infants) had mothers who experienced prolonged ROM. While ROM did not immediately impact beta diversity or ARG diversity in the meconium resistome, it did have a lasting effect on the stool resistome, resulting in an increased number of unique ARGs and ARG diversity. Prolonged ROM also significantly influenced the abundance of various ARGs spanning multiple drug classes, including macrolides, diaminopyrimidine, beta-lactam, Phenicol, and tetracycline, within the stool samples of preterm infants. Infants born to mothers who experienced ROM for longer than 18 hours exhibited a higher abundance of ARGs associated with multidrug resistance. Among the identified bacterial species, *E. coli* stood out as the sole carrier of multidrug resistance, displaying an increased abundance as preterm infants matured. This finding aligns with recent studies highlighting *E. coli* as a major contributor to the infant resistome and contributes to a higher proportion of multidrug resistance in preterm infants (49, 50). Overall, these findings underscore the intricate relationship between maternal health during gestation, particularly the duration of ROM, and the development of microbial resistance in neonates, shedding light on the complex interplay influencing microbial health in this population.

Despite challenges such as constrained sample size and the lack of environmental data, our study provides valuable insights into the intricate interactions among the preterm infant’s resistome, microbiome, and adverse outcomes. Leveraging metagenomic sequencing, we delve into the distribution of ARGs within the gut microbiota at the species level. While multiple filtering techniques were used to mitigate misclassification risks, future studies should incorporate ultra-deep sequencing to enhance accuracy. Additionally, DNA sequencing served as a proxy for ARG transcription, but further studies are needed to validate these findings through direct RNA analysis. Moreover, our examination of meconium, although sometimes delayed due to late passage, provides a unique glimpse into the initial gut microbial landscape, capturing the state of the intrauterine environment and shedding light on maternal-fetal microbial interactions, early colonization, and potential long-term health consequences (51). By analyzing a longitudinal series of meconium and stool samples from the same neonates, we can trace the evolution of the microbiome, reflecting the sustained influence of external factors on the gut.

Furthermore, neonatal research frequently encounters limitations in studies examining microbial and ARG diversity relative to antibiotic treatments in neonates; such studies often lack the statistical power necessary for conclusive findings and rely solely on *P* values (52, 53). This reliance on *P* values can be misleading, as it does not adequately convey the clinical relevance of observed differences (54), thus obscuring the true influence of antibiotics or other factors on the premature infant resistome and microbiome. Given that both effect size and sample size can sway *P* values, there is a potential for falsely significant outcomes with little clinical value due to minuscule differences between groups (52). Unlike *P* values, effect size offers a consistent metric independent of sample size, allowing for a more reliable interpretation of results. Consequently, acknowledging the vulnerabilities inherent in *P* value-based analyses, especially in our limited sample size, we have employed effect size as our evaluative measure throughout this study.

Our study found a high prevalence of ARGs in preterm infants irrespective of direct antibiotic exposure, which intensified with age in parallel to the developing microbiome. Specifically, antibiotic-treated infants harbored more beta-lactam-associated ARGs and exhibited augmented antibiotic tolerance mechanisms. The presence of ARGs unrelated to drugs administered to the neonate or mother during gestation or delivery suggests maternal antibiotic utilization outside of the gestational period, along with environmental exposures, can impact the premature infant resistome and microbiome. Maternal health and antibiotic use during pregnancy and delivery were linked to alterations in the infant’s resistome and microbiome. These results warrant cautious antibiotic application and call for continued improvements in antenatal care, such as earlier microbial screenings and alternative approaches to reduce the incidence of prolonged ROM. By preventing these pregnancy-related complications, we can decrease the dependence on maternal antibiotic utilization, which can potentially prevent the further spread of ARGs to preterm infants. Future studies should better emphasize exploring the maternal microbiome during gestation in shaping neonatal health outcomes. Evaluating maternal health in tandem with infant outcomes is crucial to understanding factors influencing early microbial colonization and antimicrobial resistance/tolerance. This integrated approach may reveal critical windows for intervention, allowing for improved health outcomes and refinement of current care practices, ensuring that interventions are tailored to protect and promote health in the immediate and long term.

## Data Availability

Raw sequences were submitted to NCBI SRA and can be accessed with BioProject accession number PRJNA1095378.

## SUPPLEMENTAL MATERIAL

Supplemental material msphere.00286-24-s0001.docx Supplemental figures and tables.

ASM does not own the copyrights to Supplemental Material that may be linked to, or accessed through, an article. The authors have granted ASM a non-exclusive, world-wide license to publish the Supplemental Material files. Please contact the corresponding author directly for reuse.
