# Supplementary material for: Increased antibiotic resistance in preterm neonates under early antibiotic use
Source: mSphere. 2024 Oct 7;9(10):e00286-24. doi: 10.1128/msphere.00286-24 (PMC11542550; doi:10.1128/msphere.00286-24)
Supplement: Supplemental material — Supplemental figures and tables. [file msphere.00286-24-s0001.docx]

**Supplementary Materials**

**ST1**. Comprehensive overview of identified ARGS (n=175) based on alignment to Comprehensive Antibiotic Resistance Database (CARD), corresponding antibiotic classes (n=15), and Inhibition Mechanisms Across 60 samples (including meconium and stool).


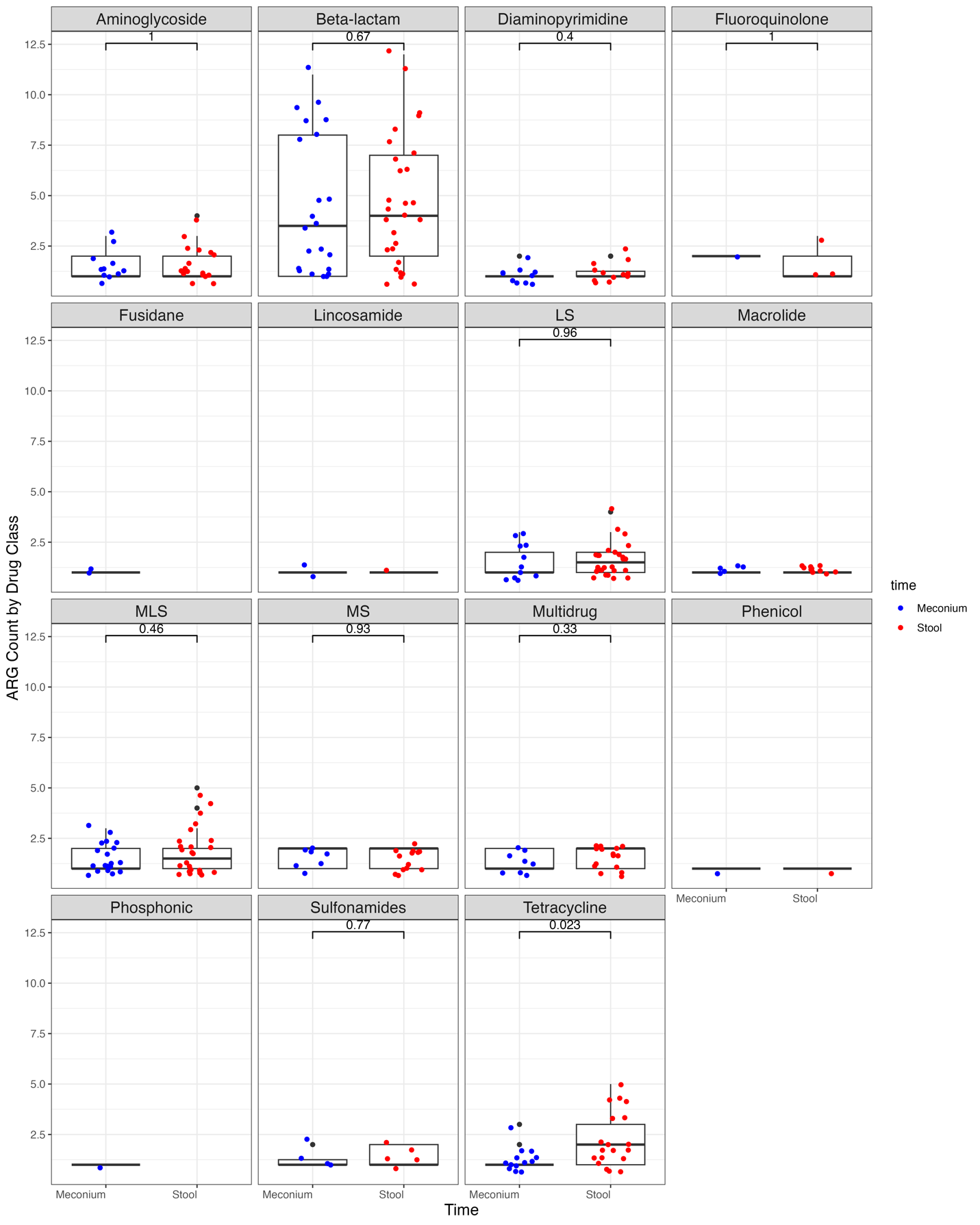


**SM 1**. Summary of the number of unique ARGS based on drug class assignments and sampling timeframe (Meconium= 30) and (Stool= 30).


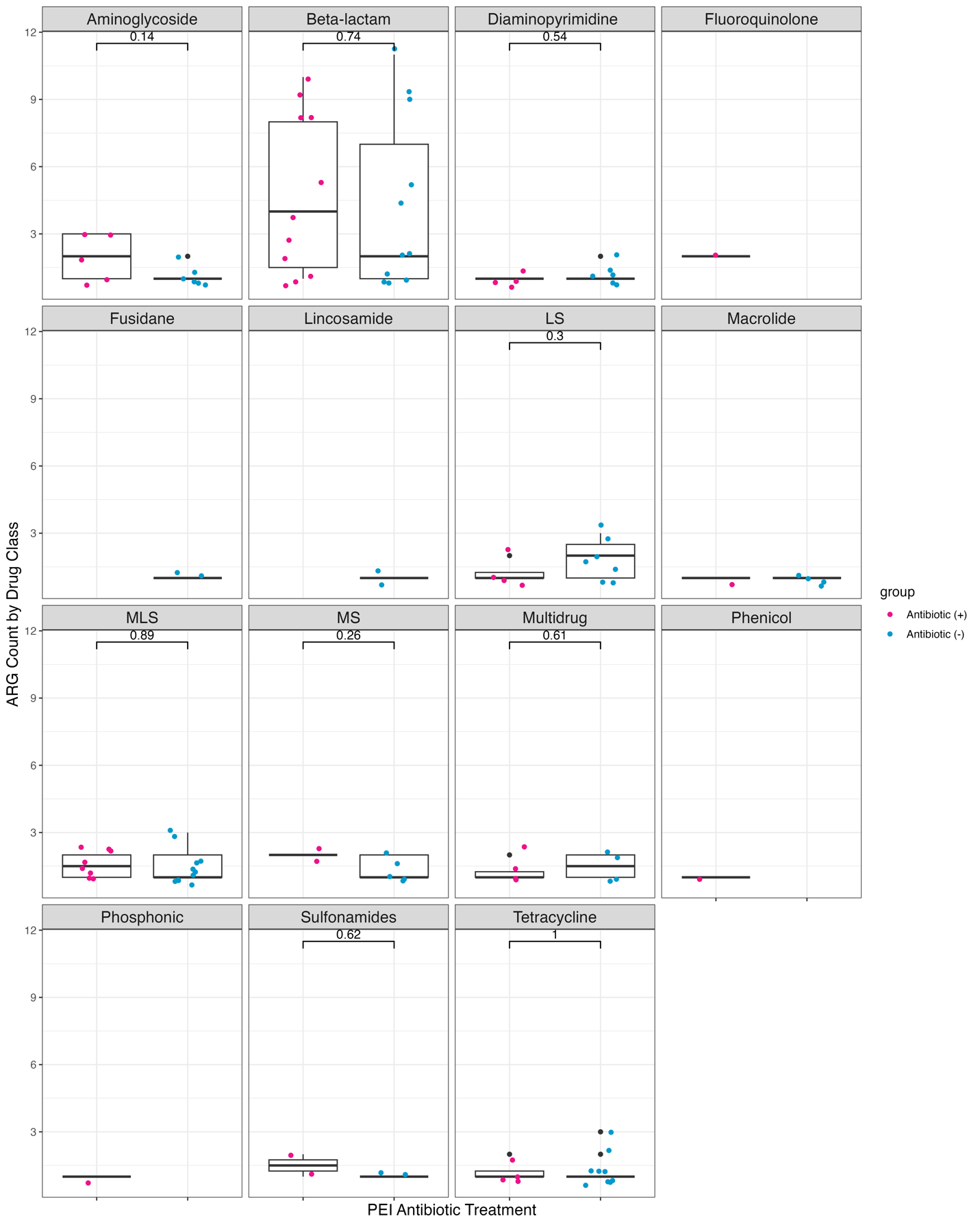


**SM 2**. Summary of the number of unique ARGS based on drug class assignments from meconium samples based on neonatal antibiotic utilization.


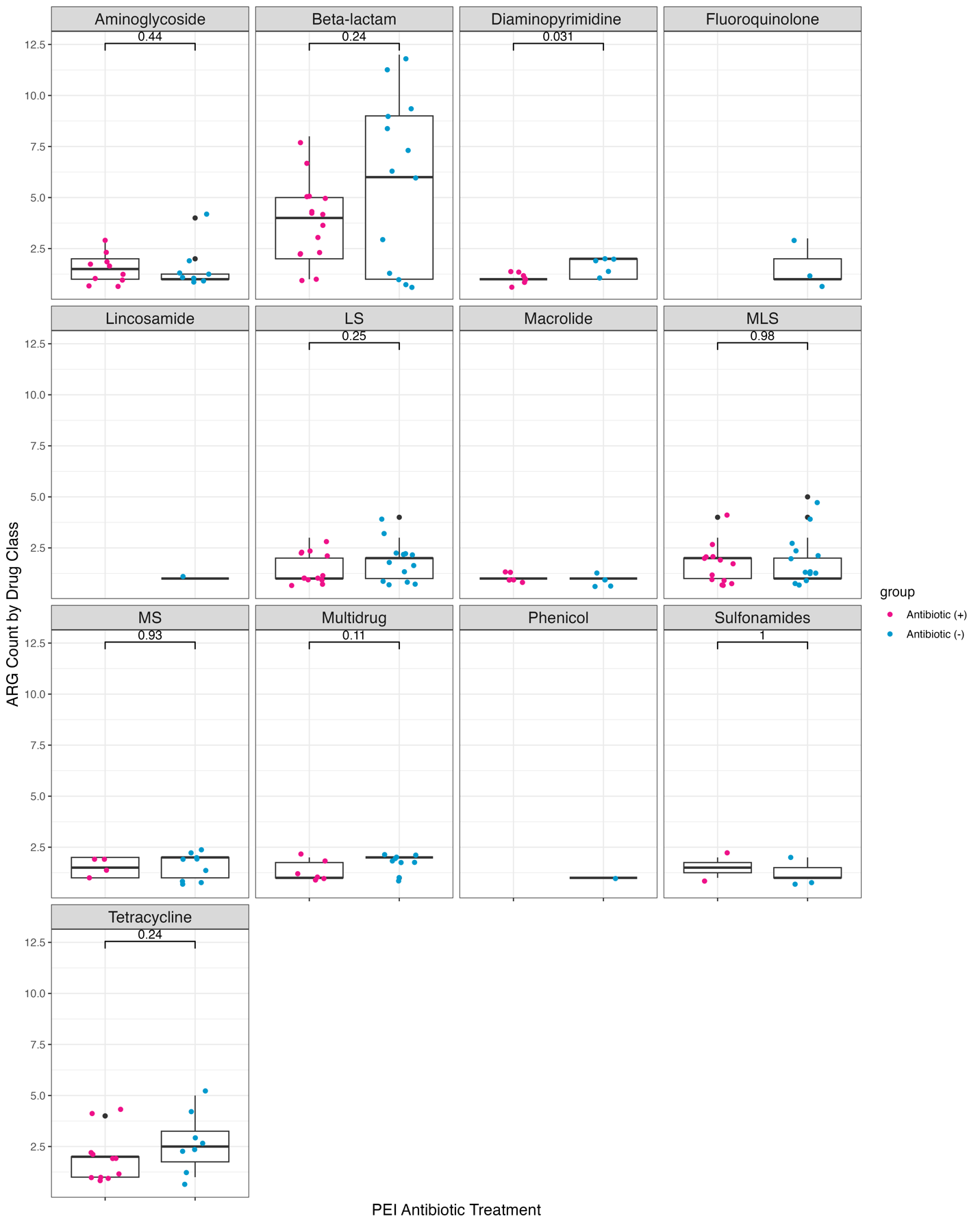


**SM 3**. Summary of the number of unique ARGS based on drug class assignments from stool samples based on neonatal antibiotic utilization.


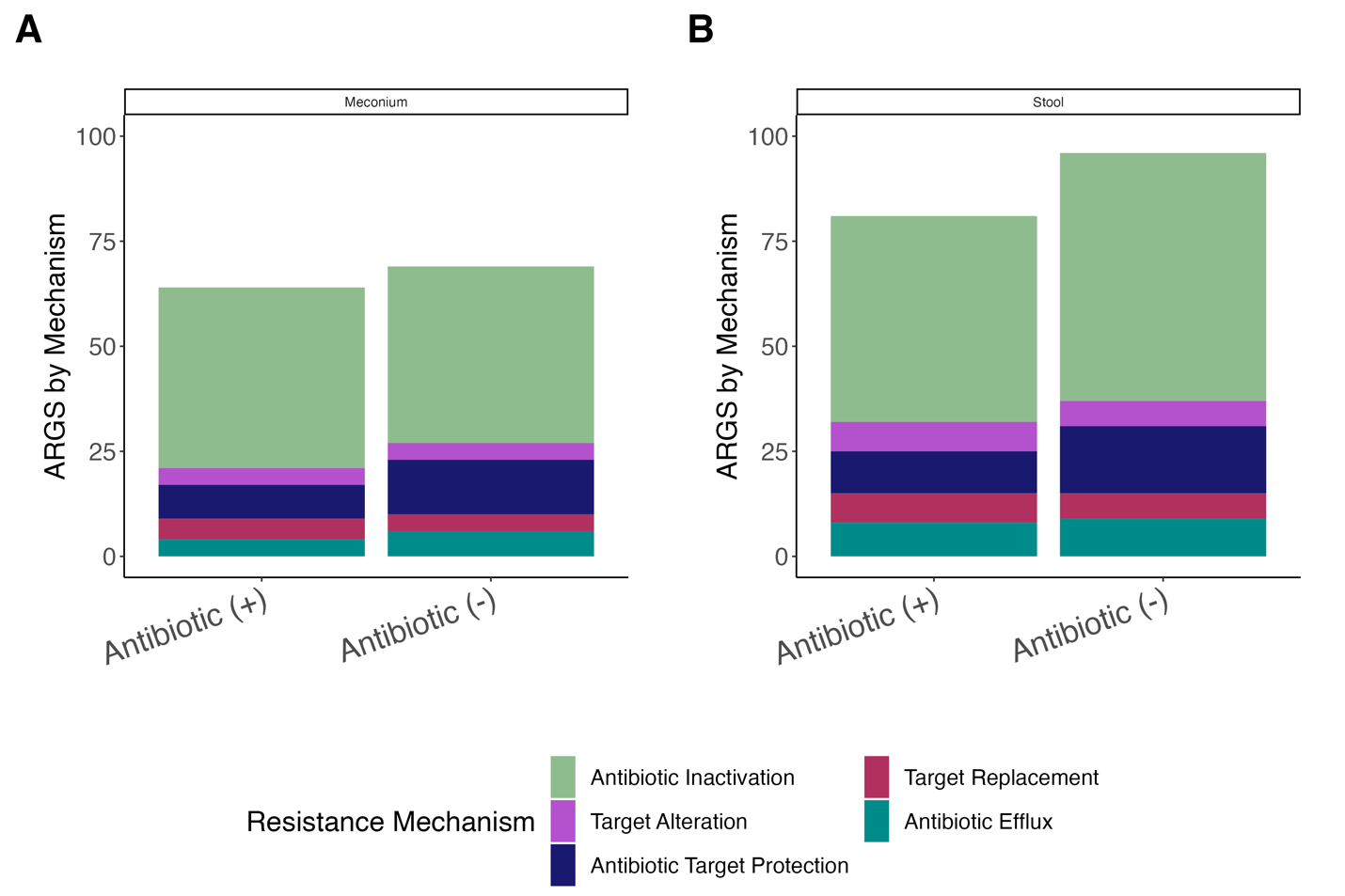


**SM 4**. A breakdown of the antibiotic resistance mechanisms was identified in each PEI antibiotic treatment regime and sampling timeframe. Neonatal antibiotic treatment is denoted as Antibiotic (+).


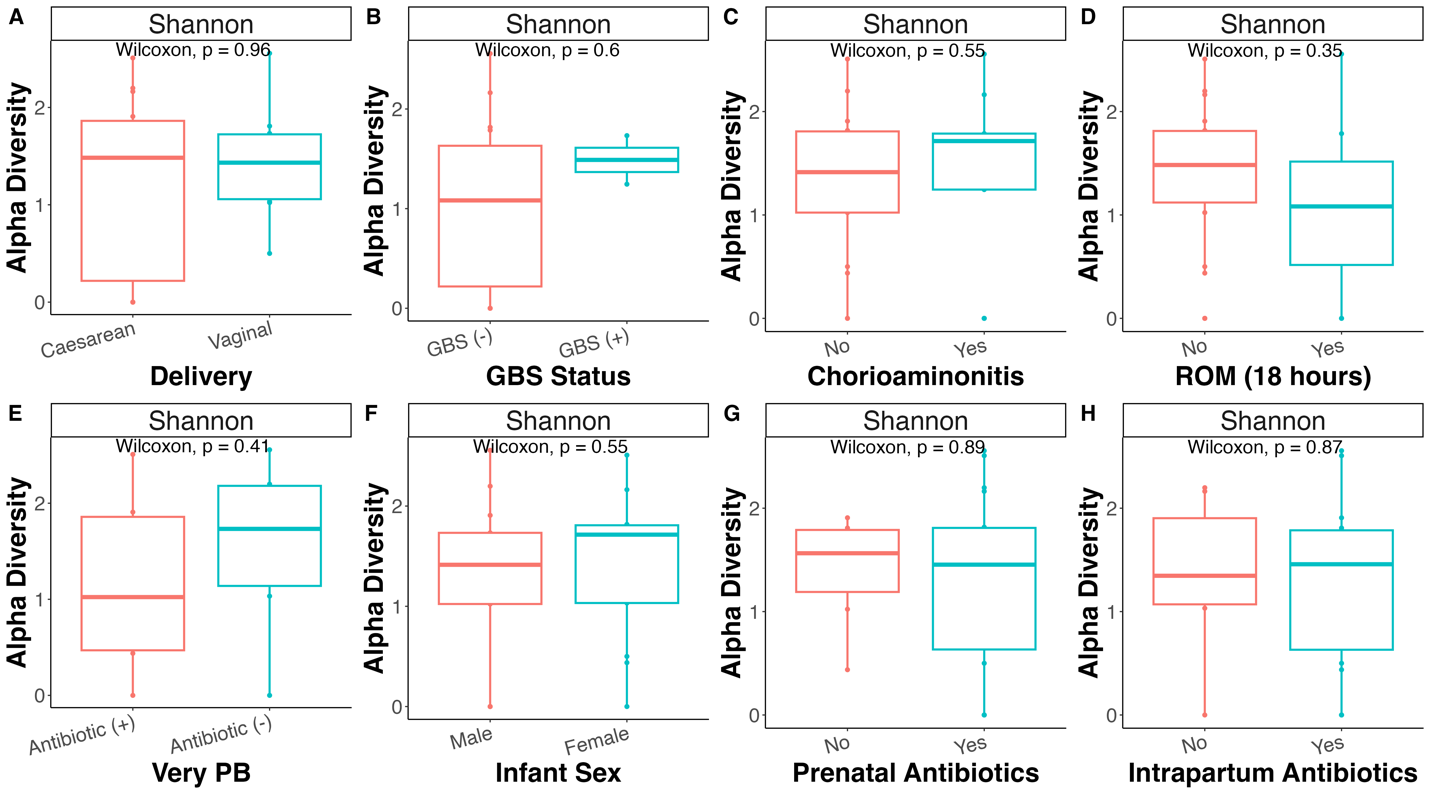


**SM 5**. Variations in Alpha Diversity of the Preterm Infant (PEI) Meconium Resistome across different factors: (**A**) delivery mode, (**B**) Group B Streptococcus (GBS) status, (**C**) Chorioamnionitis, (**D**) ruptured membranes for more than 18 hours, and (E) gestational age. Focusing on very premature infants with neonatal antibiotic treatment is denoted as Antibiotic (+). (F) Infant sex and maternal antibiotic utilization: (G) prenatal care and (H) intrapartum.


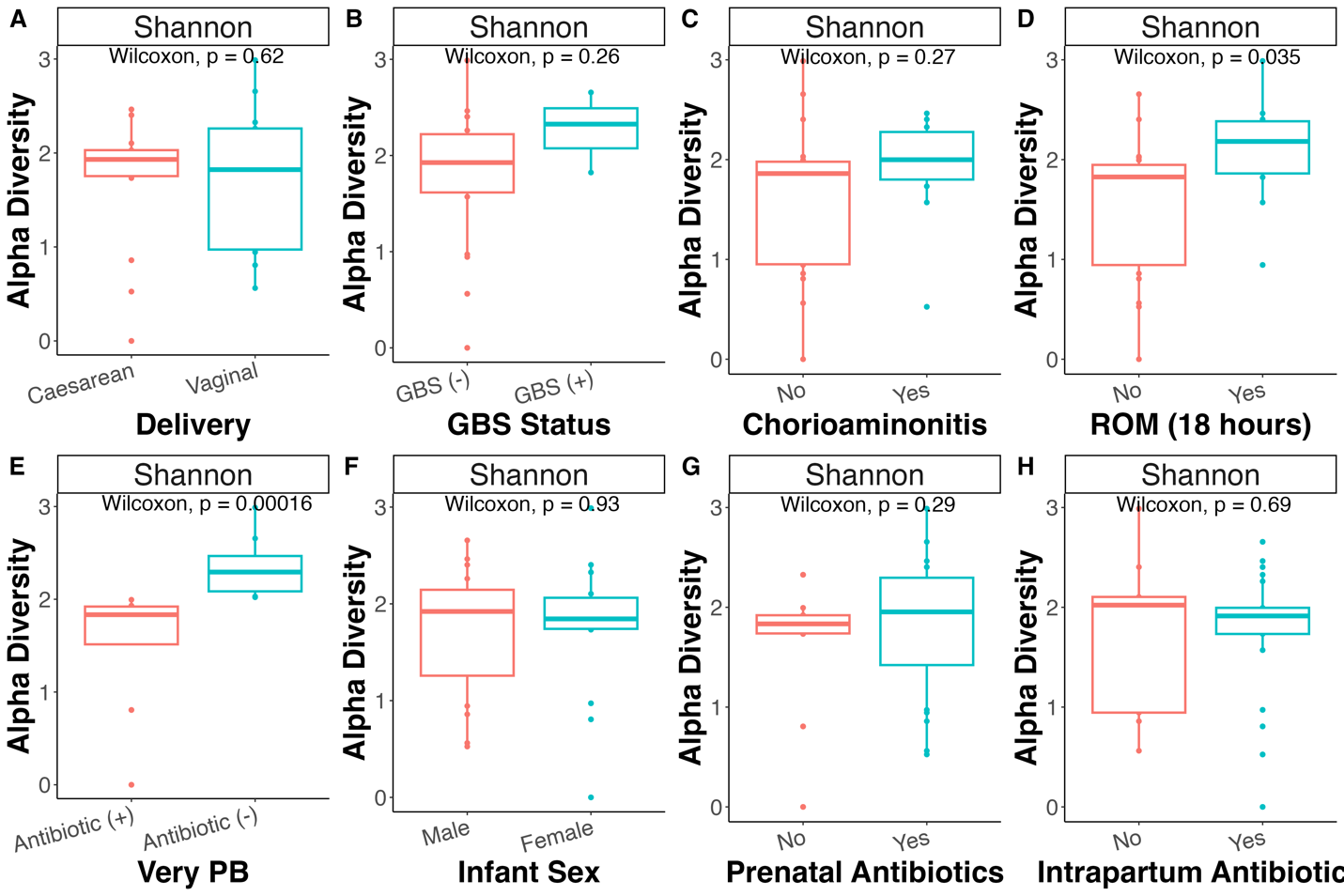


**SM 6**. Variations in Alpha Diversity of the Preterm Infant (PEI) Stool Resistome across different factors: (**A**) delivery mode, (**B**) Group B Streptococcus (GBS) status, (**C**) Chorioamnionitis, (**D**) ruptured membranes for more than 18 hours, (**E**) gestational age, focusing on very premature infants with neonatal antibiotic treatment is denoted as Antibiotic (+), (**F**) Infant sex and maternal antibiotic utilization: (**G**) prenatal care, and (**H**) intrapartum.


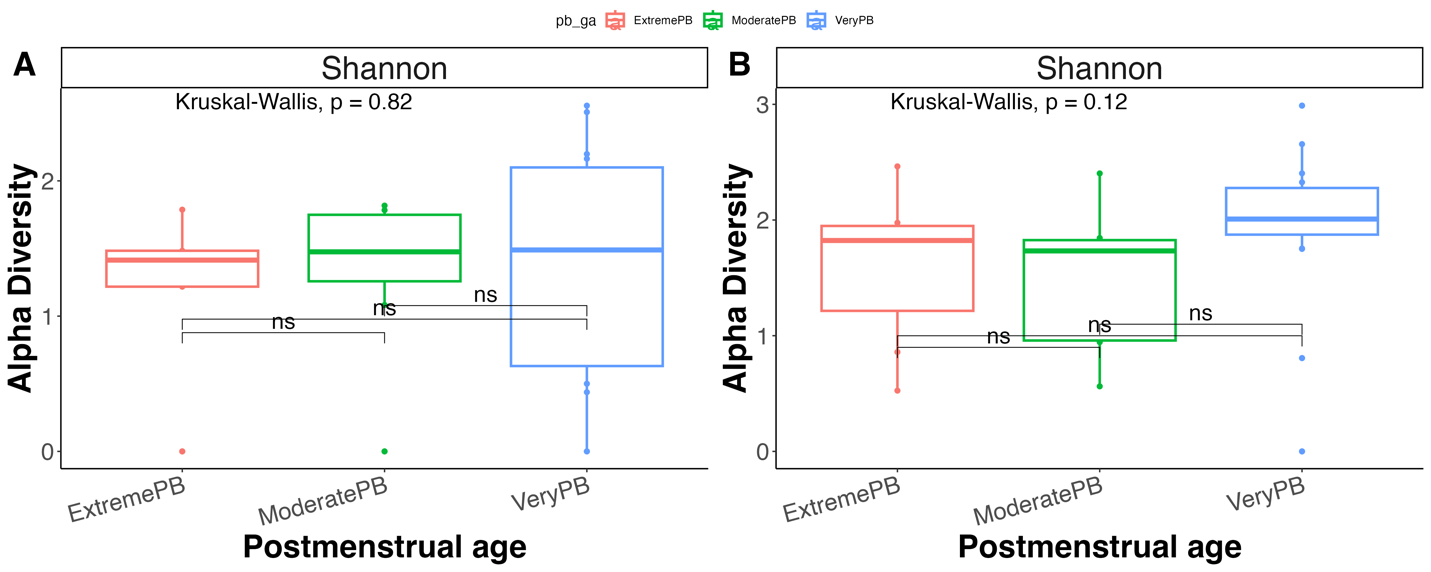


**SM 7.** ARG diversity in meconium (**A**) and Stool (**B**) resistome based on postmenstrual age (PMA). PMA was categorized into three groups to account for differences in developmental stages: Extremely Preterm Birth (PB) (<28 weeks, n=7), Very PB (28 to <32 weeks, n=16), and Moderately PB (32 to <37 weeks, n=7).


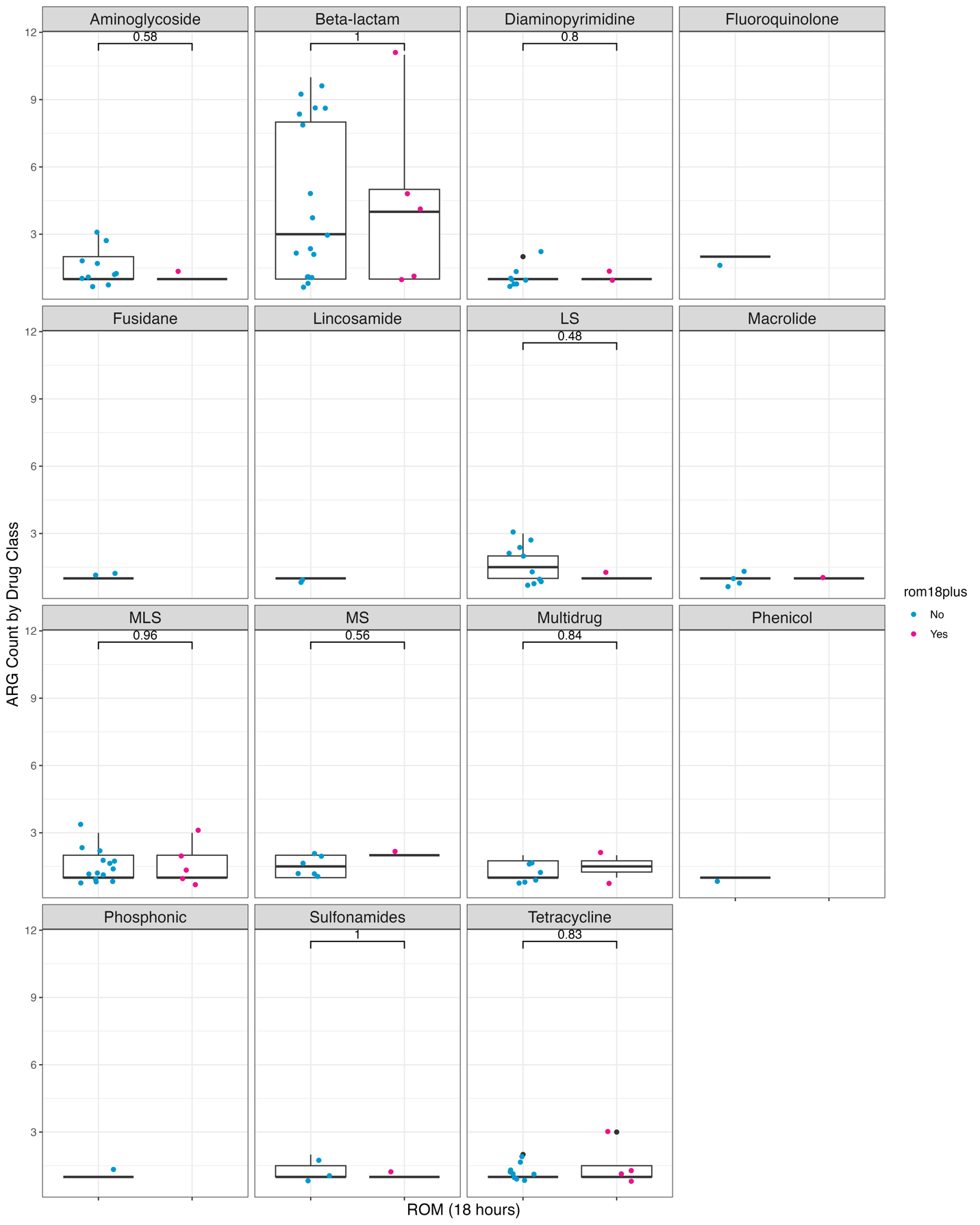


**SM 8**. Summary of the number of unique ARGS based on drug class assignments from meconium samples based on prolonged membrane rupture. Yes, represents PEIs from mothers who experienced prolonger membrane rupture of over 18 hours (n=10/30 PEIs).


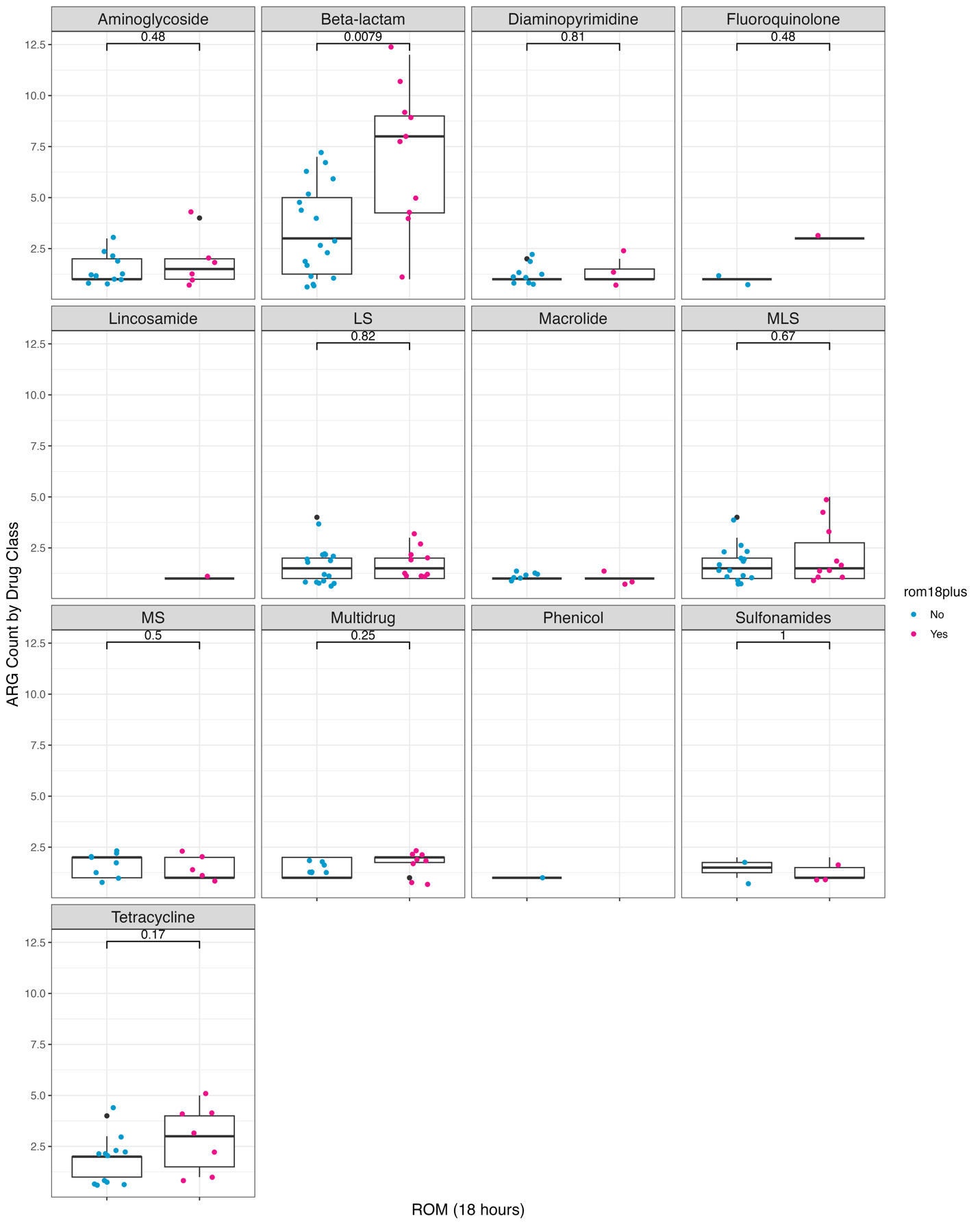


**SM 9**. Summary of the number of unique ARGS based on drug class assignments from stool samples based on prolonged membrane rupture. Yes, represents PEI from mothers who experienced prolonger membrane rupture of over 18 hours (n=10/30 PEIs).


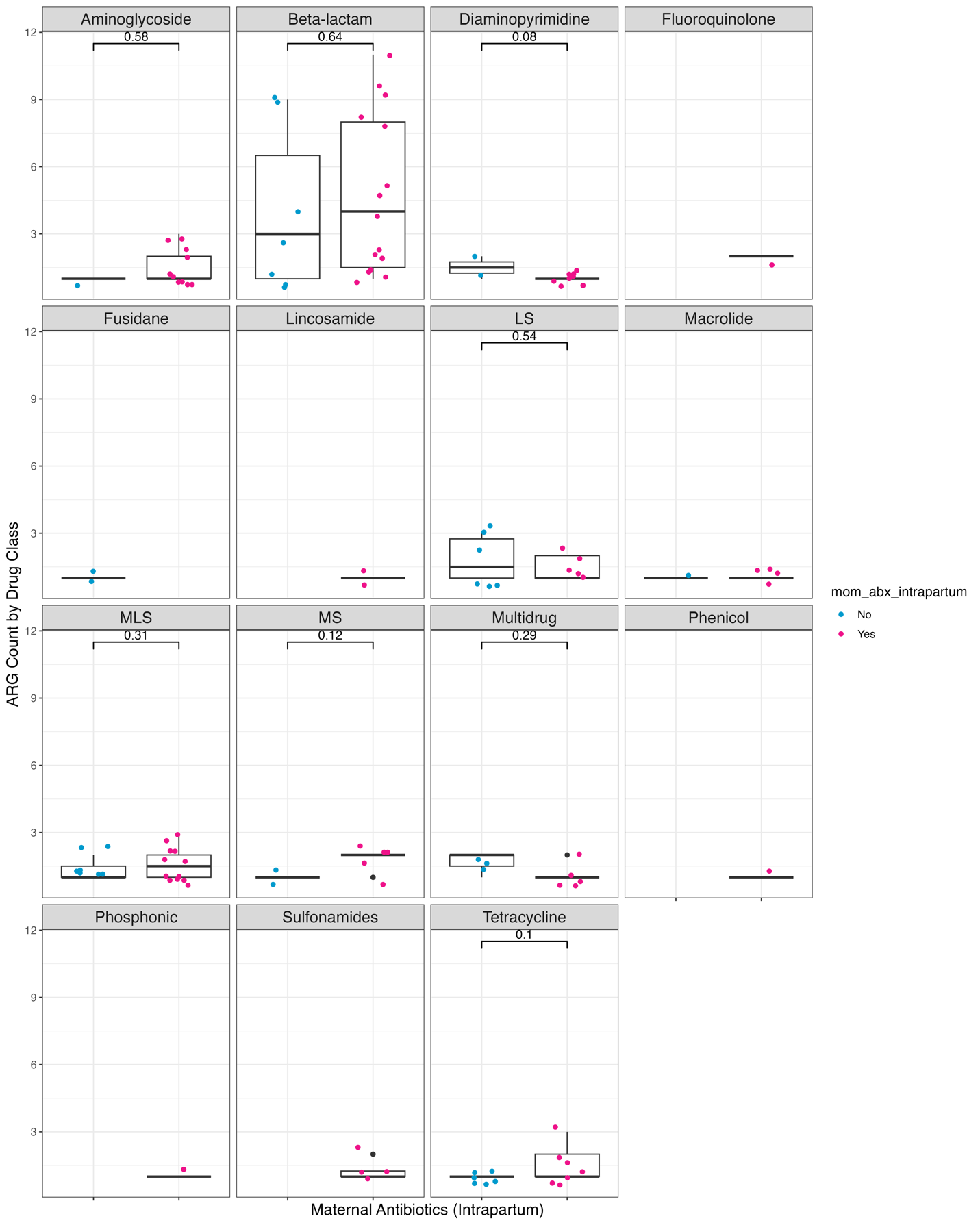


**SM 10**. Summary of the number of unique ARGS based on drug class assignments from meconium samples based on intrapartum antibiotics. (n=21/30 PEIs).


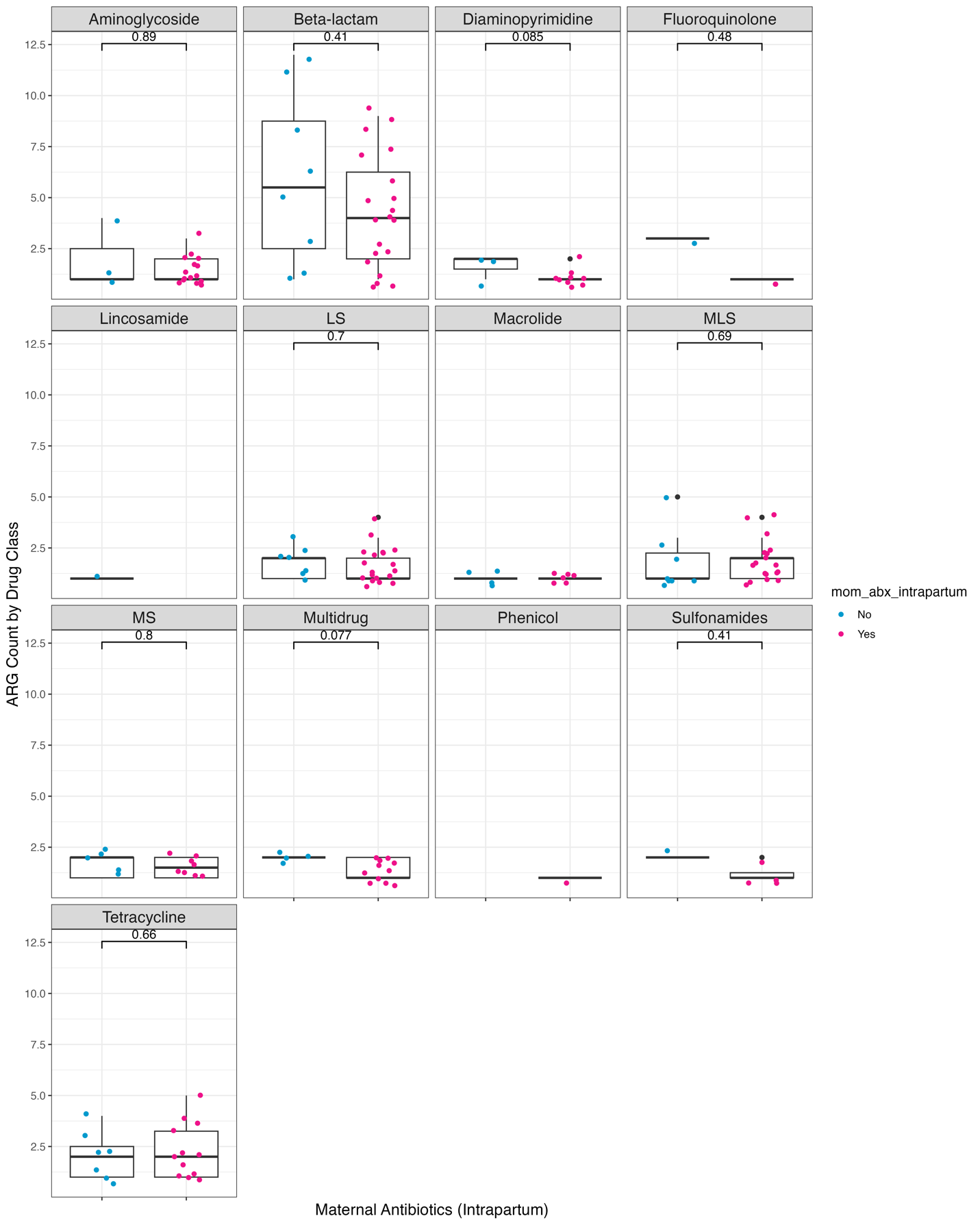


**SM11**. Summary of the number of unique ARGS based on drug class assignments from stool samples based on intrapartum antibiotics. (n=21/30 PEIs).


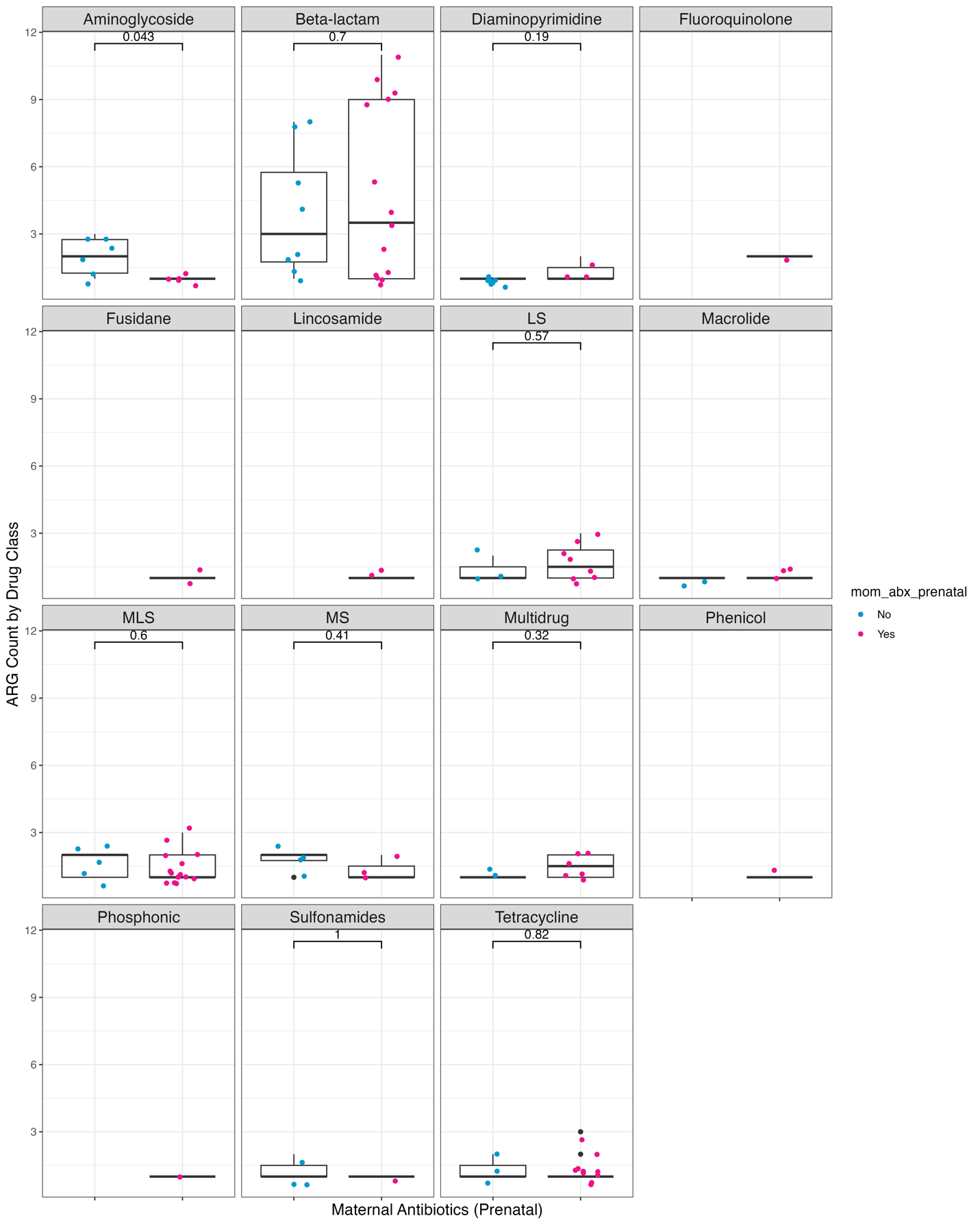


**SM12**. Summary of the number of unique ARGS based on drug class assignments from meconium samples based on prenatal antibiotics. (n=20/30 PEIs).


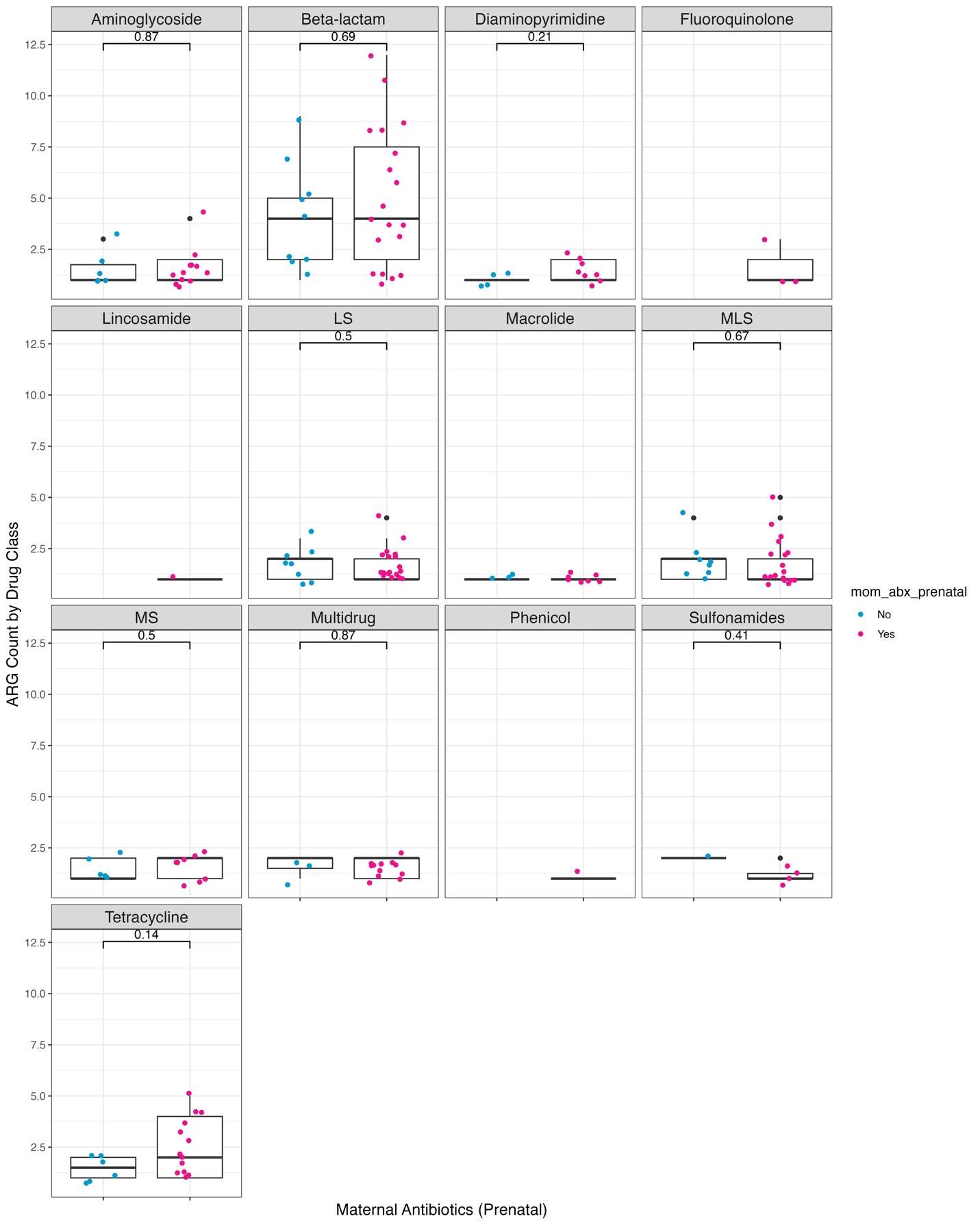


**SM13**. Summary of the number of unique ARGS based on drug class assignments from stool samples based on prenatal antibiotics. (n=20/30 PEIs).


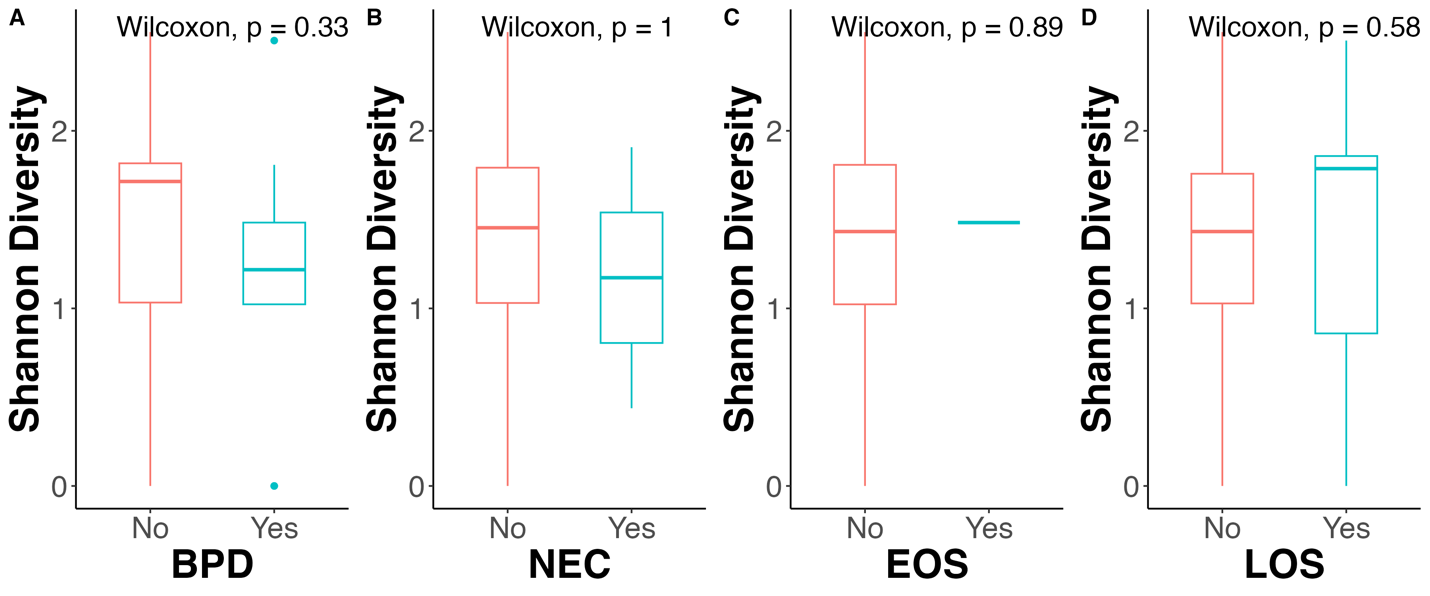


**SM 14.** Differences in Alpha Diversity of the PEI Meconium Resistome based on Health Outcomes. **(A)** Bronchopulmonary Dysplasia (BDP), (**B**) Necrotizing Enterocolitis (NEC), (**C**) Early-Onset Sepsis (EOS), and (D) Late-Onset Sepsis (LOS).


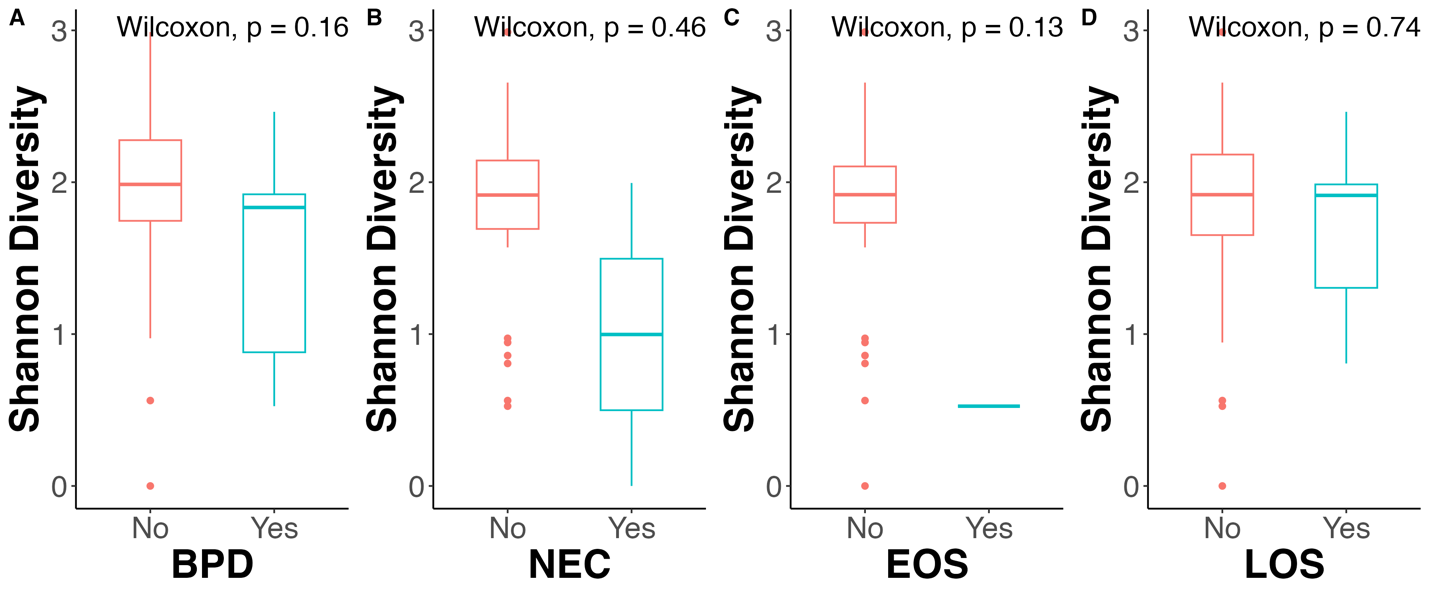


**SM 15.** Differences in Alpha Diversity of the PEI Stool Resistome based on Health Outcomes. **(A)** Bronchopulmonary Dysplasia (BDP), (**B**) Necrotizing Enterocolitis (NEC), (**C**) Early-onset sepsis (EOS), (**D**) Late-onset sepsis (LOS).

**ST2: PERMANOVA Analysis of Antibiotic Treatment Impact on the PEI Meconium Resistome and Microbiome Composition.**

| **Antibiotic Utilization** | **Resistome** | | | **Microbiome** | | |
| --- | --- | --- | --- | --- | --- | --- |
|  | *R^2^* | *F* | *p-value* | *R^2^* | *F* | *p-value* |
| **Antenatal Care (ANC)** | | | | | | |
| Azithromycin | 0.03695 | 0.8954 | 0.597 | 0.02163 | 0.5856 | 0.885 |
| Ampicillin | 0.03988 | 0.9665 | 0.496 | 0.02291 | 0.6202 | 0.843 |
| Cefazolin | 0.03491 | 0.8461 | 0.694 | 0.02692 | 0.7287 | 0.746 |
| Amoxicillin | 0.04114 | 0.9971 | 0.472 | 0.06675 | 1.8072 | **0.056 *** |
| Gentamicin | 0.03082 | 0.7469 | 0.847 | 0.03566 | 0.9654 | 0.489 |
| Abx Combinations | 0.02884 | 0.699 | 0.881 | 0.03747 | 1.0145 | 0.424 |
| **Intrapartum** | | | | | | |
| Amoxicillin | 0.04057 | 0.9833 | 0.513 | 0.01906 | 0.516 | 0.923 |
| Ampicillin | 0.08236 | 1.9961 | **0.006 **** | 0.06696 | 1.8128 | **0.054 *** |
| Azithromycin | 0.03641 | 0.8824 | 0.618 | 0.03189 | 0.8634 | 0.559 |
| Gentamicin | 0.04717 | 1.1431 | 0.281 | 0.03468 | 0.9391 | 0.518 |
| **PEI** | | | | | | |
| Ampicillin | 0.03562 | 0.8632 | 0.623 | 0.03623 | 0.9809 | 0.432 |
| Ceftazidime | 0.02954 | 0.716 | 0.885 | 0.05557 | 1.5044 | 0.106 |
| Cefotaxime | 0.02306 | 0.5589 | 0.976 | 0.01391 | 0.3765 | 0.988 |
| Piperacillin | 0.05158 | 1.2499 | 0.176 | 0.05257 | 1.4232 | 0.143 |
| Metronidazole | 0.03724 | 0.9025 | 0.589 | 0.01302 | 0.3524 | 0.99 |
| Vancomycin | 0.03554 | 0.8614 | 0.696 | 0.01769 | 0.479 | 0.946 |
| Azithromycin | 0.03571 | 0.8655 | 0.684 | 0.02291 | 0.6203 | 0.834 |
| Ampicillin & Gentamicin | 0.04379 | 1.0613 | 0.395 | 0.01788 | 0.4841 | 0.94 |

This PERMANOVA assesses the relationship between PEI resistome and intestinal microbiome and their relationship with antibiotic utilization. Variables demonstrating a statistically significant p-value have been denoted with **, while marginally significant p-values are marked with *. Time was considered a confounding factor in this analysis.

**ST3: PERMANOVA Analysis of Antibiotic Treatment Impact on the PEI Stool Resistome and Microbiome Composition.**

| **Antibiotic Utilization** | **Resistome** | | | **Microbiome** | | |
| --- | --- | --- | --- | --- | --- | --- |
|  | *R^2^* | *F* | *p-value* | *R^2^* | *F* | *p-value* |
| **Antenatal Care (ANC)** | | | | | | |
| Azithromycin | 0.02353 | 0.6882 | 0.899 | 0.03151 | 1.0454 | 0.411 |
| Ampicillin | 0.02716 | 0.7942 | 0.791 | 0.03288 | 1.0908 | 0.346 |
| Cefazolin | 0.04151 | 1.2138 | 0.214 | 0.03437 | 1.1402 | 0.302 |
| Amoxicillin | 0.03625 | 1.0601 | 0.389 | 0.04678 | 1.5519 | 0.101 |
| Gentamicin | 0.02419 | 0.7074 | 0.877 | 0.01746 | 0.5794 | 0.86 |
| Abx Combinations | 0.02128 | 0.6222 | 0.943 | 0.02678 | 0.8885 | 0.572 |
| **Intrapartum** | | | | | | |
| Amoxicillin | 0.03476 | 1.0165 | 0.454 | 0.02285 | 0.758 | 0.693 |
| Ampicillin | 0.04916 | 1.4377 | **0.061*** | 0.04442 | 1.4739 | 0.152 |
| Azithromycin | 0.03847 | 1.1249 | 0.303 | 0.04534 | 1.5042 | 0.13 |
| Gentamicin | 0.03323 | 0.9716 | 0.502 | 0.02529 | 0.8391 | 0.607 |
| **PEI** | | | | | | |
| Ampicillin | 0.05212 | 1.5242 | **0.046**** | 0.05185 | 1.7201 | **0.079*** |
| Ceftazidime | 0.06307 | 1.8444 | **0.006**** | 0.08044 | 2.6687 | **0.003**** |
| Cefotaxime | 0.0193 | 0.5644 | 0.977 | 0.0092 | 0.3052 | 0.994 |
| Piperacillin | 0.0414 | 1.2106 | 0.201 | 0.05287 | 1.754 | **0.069*** |
| Metronidazole | 0.02514 | 0.7352 | 0.843 | 0.01953 | 0.6479 | 0.81 |
| Vancomycin | 0.02794 | 0.8171 | 0.722 | 0.04513 | 1.4972 | 0.139 |
| Azithromycin | 0.02924 | 0.855 | 0.689 | 0.03847 | 1.2764 | 0.214 |
| Ampicillin & Gentamicin | 0.03607 | 1.0547 | 0.399 | 0.0433 | 1.4365 | 0.147 |

This PERMANOVA assesses the relationship between PEI resistome and intestinal microbiome and their relationship with antibiotic utilization. Variables demonstrating a statistically significant p-value have been denoted with **, while marginally significant p-values are marked with *. Time was considered a confounding factor in this analysis.


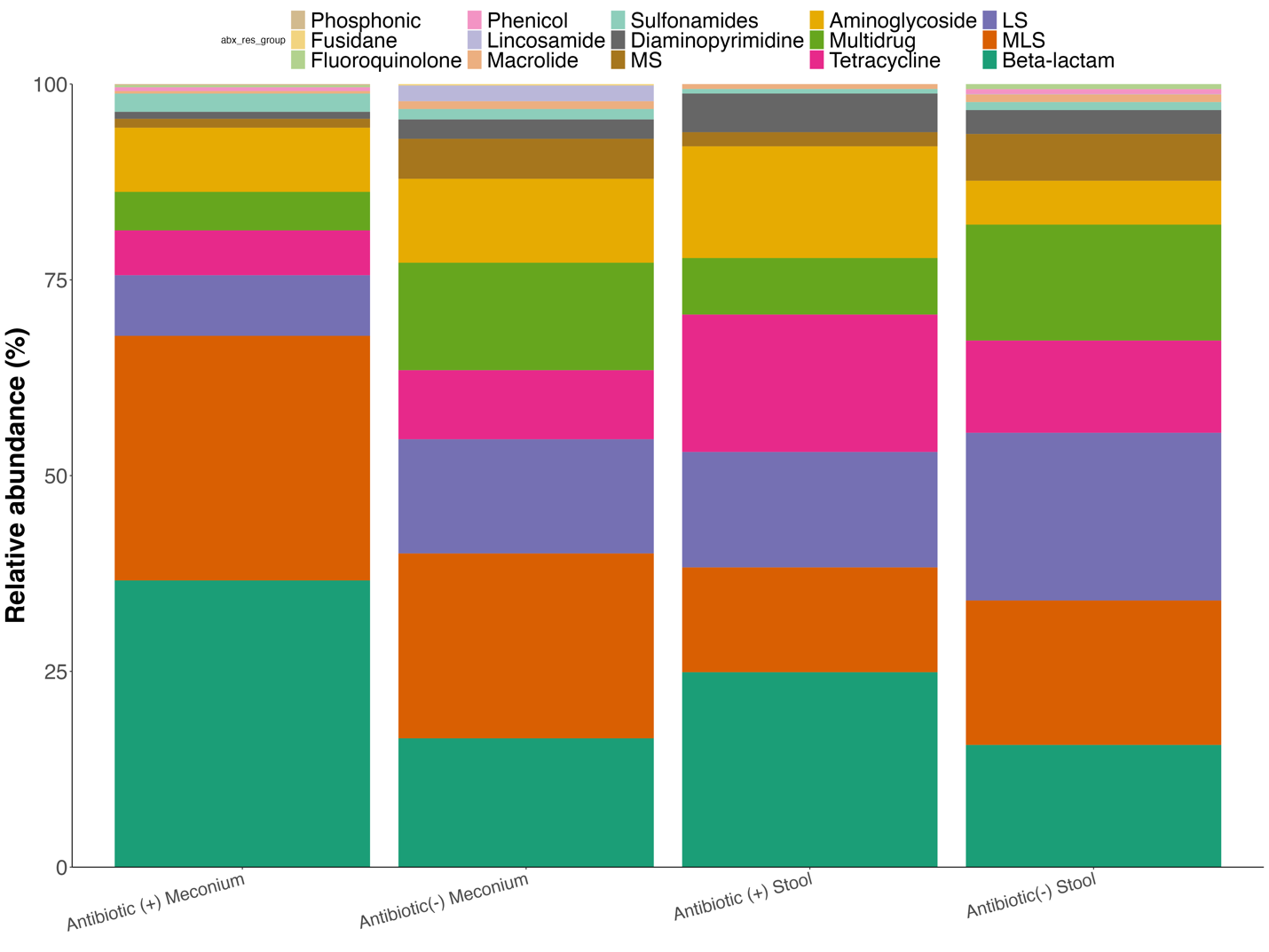


**SM 16**. **Relative abundance of drug resistance classes based on neonatal antibiotic treatment and sample timeline**. Of the 175 ARGs identified, they corresponded to 15 different drug classes.

**ST 4. Differential Abundance of Resistome over Time using Wilcoxon Signed Rank test. (n=56 samples). ARGs are arranged in order of effect size (greatest to least).**

| **ARG** | **p-value** | **Effect Size** | **Effect Magnitude** | **ARG (Drug Class)** |
| --- | --- | --- | --- | --- |
| tetK | 0.00046423 | 0.46888427 | moderate | Tetracycline |
| ANT(4')-Ib | 0.0016512 | 0.42159433 | moderate | Aminoglycoside |
| mecA | 0.00174356 | 0.41946306 | moderate | Beta-lactam |
| fusB | 0.00236249 | 0.40739125 | moderate | Fusidane |
| TEM-127 | 0.00293294 | 0.39860653 | moderate | Beta-lactam |
| TEM-198 | 0.00362654 | 0.38982182 | moderate | Beta-lactam |
| TEM-135 | 0.00424125 | 0.38323328 | moderate | Beta-lactam |
| TEM-206 | 0.00470203 | 0.37884092 | moderate | Beta-lactam |
| TEM-28 | 0.00470203 | 0.37884092 | moderate | Beta-lactam |
| TEM-70 | 0.00470203 | 0.37884092 | moderate | Beta-lactam |
| CTX-M-103 | 0.00520767 | 0.37444856 | moderate | Beta-lactam |
| CTX-M-22 | 0.00520767 | 0.37444856 | moderate | Beta-lactam |
| CTX-M-3 | 0.00520767 | 0.37444856 | moderate | Beta-lactam |
| CTX-M-33 | 0.00520767 | 0.37444856 | moderate | Beta-lactam |
| CTX-M-72 | 0.00520767 | 0.37444856 | moderate | Beta-lactam |
| CTX-M-88 | 0.00520767 | 0.37444856 | moderate | Beta-lactam |
| SHV-110 | 0.00520767 | 0.37444856 | moderate | Beta-lactam |
| APH(3')-IIb | 0.00547848 | 0.37225238 | moderate | Aminoglycoside |
| catB7 | 0.00547848 | 0.37225238 | moderate | Phenicol |
| FosA | 0.00547848 | 0.37225238 | moderate | Phosphonic |
| OXA-50 | 0.00547848 | 0.37225238 | moderate | Beta-lactam |
| OXY-1-6 | 0.00547848 | 0.37225238 | moderate | Beta-lactam |
| QnrB47 | 0.00547848 | 0.37225238 | moderate | Fluoroquinolone |
| QnrB50 | 0.00547848 | 0.37225238 | moderate | Fluoroquinolone |
| SHV-51 | 0.00547848 | 0.37225238 | moderate | Beta-lactam |
| SHV-71 | 0.00547848 | 0.37225238 | moderate | Beta-lactam |
| msrC | 0.00576194 | 0.3700562 | moderate | MS |
| SHV-134 | 0.00576194 | 0.3700562 | moderate | Beta-lactam |
| SHV-42 | 0.00576194 | 0.3700562 | moderate | Beta-lactam |
| vgaA | 0.00621285 | 0.36675566 | moderate | LS |
| ErmC | 0.00795064 | 0.35577495 | moderate | MLS |
| TEM-57 | 0.0081453 | 0.35468295 | moderate | Beta-lactam |
| TEM-95 | 0.0081453 | 0.35468295 | moderate | Beta-lactam |
| tetD | 0.0081453 | 0.35468295 | moderate | Tetracycline |
| SHV-102 | 0.00897205 | 0.35029059 | moderate | Beta-lactam |
| SHV-38 | 0.00897205 | 0.35029059 | moderate | Beta-lactam |
| TEM-122 | 0.01191982 | 0.33711351 | moderate | Beta-lactam |
| TEM-141 | 0.01191982 | 0.33711351 | moderate | Beta-lactam |
| TEM-30 | 0.01191982 | 0.33711351 | moderate | Beta-lactam |
| TEM-8 | 0.01191982 | 0.33711351 | moderate | Beta-lactam |
| TEM-96 | 0.01191982 | 0.33711351 | moderate | Beta-lactam |
| CfxA4 | 0.01369348 | 0.33052498 | moderate | Beta-lactam |
| dfrA17 | 0.01369348 | 0.33052498 | moderate | Diaminopyrimidine |
| SHV-40 | 0.01369348 | 0.33052498 | moderate | Beta-lactam |
| SHV-89 | 0.01369348 | 0.33052498 | moderate | Beta-lactam |
| TEM-168 | 0.01369348 | 0.33052498 | moderate | Beta-lactam |
| vgaALC | 0.01369348 | 0.33052498 | moderate | LS |
| OXA-15 | 0.0150018 | 0.32613262 | moderate | Beta-lactam |
| OXY-1-1 | 0.0150018 | 0.32613262 | moderate | Beta-lactam |
| OXY-1-2 | 0.0150018 | 0.32613262 | moderate | Beta-lactam |
| ACT-2 | 0.01569633 | 0.32393644 | moderate | Beta-lactam |
| ACT-7 | 0.01569633 | 0.32393644 | moderate | Beta-lactam |
| SHV-137 | 0.01569633 | 0.32393644 | moderate | Beta-lactam |
| SHV-56 | 0.01569633 | 0.32393644 | moderate | Beta-lactam |
| CTX-M-82 | 0.01641899 | 0.32174026 | moderate | Beta-lactam |
| CTX-M-15 | 0.01717072 | 0.31954408 | moderate | Beta-lactam |
| SHV-81 | 0.01717072 | 0.31954408 | moderate | Beta-lactam |
| CTX-M-117 | 0.01795247 | 0.3173479 | moderate | Beta-lactam |
| TEM-143 | 0.01795247 | 0.3173479 | moderate | Beta-lactam |
| TEM-208 | 0.01961 | 0.31295554 | moderate | Beta-lactam |
| ACT-1 | 0.02234666 | 0.30636701 | moderate | Beta-lactam |
| tetM | 0.02540952 | 0.29977847 | small | Tetracycline |
| SHV-75 | 0.02882913 | 0.29318993 | small | Beta-lactam |
| lnuA | 0.02943877 | 0.29208685 | small | Lincosamide |
| SHV-96 | 0.03005381 | 0.29099375 | small | Beta-lactam |
| SHV-85 | 0.03132294 | 0.28879757 | small | Beta-lactam |
| TEM-33 | 0.03132294 | 0.28879757 | small | Beta-lactam |
| SHV-164 | 0.03331575 | 0.28549842 | small | Beta-lactam |
| TEM-82 | 0.0339996 | 0.28440521 | small | Beta-lactam |
| AAC(6')-Ii | 0.03540972 | 0.28220903 | small | Aminoglycoside |
| SHV-45 | 0.03686943 | 0.28001285 | small | Beta-lactam |
| sul2 | 0.03686943 | 0.28001285 | small | Sulfonamides |
| CfxA3 | 0.03838008 | 0.27781667 | small | Beta-lactam |
| OXY-1-3 | 0.03838008 | 0.27781667 | small | Beta-lactam |
| OXY-4-1 | 0.03838008 | 0.27781667 | small | Beta-lactam |
| SHV-106 | 0.03838008 | 0.27781667 | small | Beta-lactam |
| tetQ | 0.03838008 | 0.27781667 | small | Tetracycline |
| DHA-1 | 0.04074799 | 0.27451771 | small | Beta-lactam |
| SHV-26 | 0.04155965 | 0.27342432 | small | Beta-lactam |
| aadA24 | 0.04323132 | 0.27122814 | small | Aminoglycoside |
| dfrA1 | 0.04323132 | 0.27122814 | small | Diaminopyrimidine |
| Erm(33) | 0.04323132 | 0.27122814 | small | MLS |
| lsaA | 0.04323132 | 0.27122814 | small | LS |
| TEM-106 | 0.04323132 | 0.27122814 | small | Beta-lactam |
| TEM-166 | 0.04323132 | 0.27122814 | small | Beta-lactam |
| aadA5 | 0.04495947 | 0.26903196 | small | Aminoglycoside |
| ANT(6)-Ib | 0.04495947 | 0.26903196 | small | Aminoglycoside |
| OXY-5-2 | 0.04495947 | 0.26903196 | small | Beta-lactam |
| SHV-150 | 0.04495947 | 0.26903196 | small | Beta-lactam |
| SHV-48 | 0.04495947 | 0.26903196 | small | Beta-lactam |
| SHV-66 | 0.04495947 | 0.26903196 | small | Beta-lactam |
| SHV-79 | 0.04495947 | 0.26903196 | small | Beta-lactam |
| tet38 | 0.04495947 | 0.26903196 | small | Tetracycline |
| tetA | 0.04495947 | 0.26903196 | small | Tetracycline |
| tetL | 0.04495947 | 0.26903196 | small | Tetracycline |
| ACT-15 | 0.04859088 | 0.2646396 | small | Beta-lactam |
| ACT-5 | 0.04859088 | 0.2646396 | small | Beta-lactam |
| APH(3')-Ia | 0.04859088 | 0.2646396 | small | Aminoglycoside |
| SHV-153 | 0.04859088 | 0.2646396 | small | Beta-lactam |
| AAC(6')-Ic | 0.05049704 | 0.26244342 | small | Aminoglycoside |
| CTX-M-101 | 0.05049704 | 0.26244342 | small | Beta-lactam |
| ErmG | 0.05049704 | 0.26244342 | small | MLS |
| ErmT | 0.05049704 | 0.26244342 | small | MLS |
| OXY-2-8 | 0.05049704 | 0.26244342 | small | Beta-lactam |
| QnrB60 | 0.05049704 | 0.26244342 | small | Fluoroquinolone |
| SHV-101 | 0.05049704 | 0.26244342 | small | Beta-lactam |
| SHV-11 | 0.05049704 | 0.26244342 | small | Beta-lactam |
| SHV-165 | 0.05049704 | 0.26244342 | small | Beta-lactam |
| SHV-178 | 0.05049704 | 0.26244342 | small | Beta-lactam |
| SHV-5 | 0.05049704 | 0.26244342 | small | Beta-lactam |
| SHV-61 | 0.05049704 | 0.26244342 | small | Beta-lactam |
| SHV-62 | 0.05049704 | 0.26244342 | small | Beta-lactam |
| SHV-78 | 0.05049704 | 0.26244342 | small | Beta-lactam |
| TEM-104 | 0.05049704 | 0.26244342 | small | Beta-lactam |
| tetB | 0.05049704 | 0.26244342 | small | Tetracycline |
| OXY-2-7 | 0.05246547 | 0.26024724 | small | Beta-lactam |
| CfxA2 | 0.05449764 | 0.25805106 | small | Beta-lactam |
| CTX-M-139 | 0.05449764 | 0.25805106 | small | Beta-lactam |
| CTX-M-55 | 0.05449764 | 0.25805106 | small | Beta-lactam |
| OXA-347 | 0.05449764 | 0.25805106 | small | Beta-lactam |
| tetX | 0.05449764 | 0.25805106 | small | Tetracycline |
| floR | 0.05659505 | 0.25585488 | small | Phenicol |
| QnrS3 | 0.05659505 | 0.25585488 | small | Fluoroquinolone |
| QnrS4 | 0.05659505 | 0.25585488 | small | Fluoroquinolone |
| SHV-147 | 0.05659505 | 0.25585488 | small | Beta-lactam |
| SHV-28 | 0.05659505 | 0.25585488 | small | Beta-lactam |
| SHV-98 | 0.05659505 | 0.25585488 | small | Beta-lactam |
| TEM-10 | 0.05659505 | 0.25585488 | small | Beta-lactam |
| tetJ | 0.05659505 | 0.25585488 | small | Tetracycline |
| SHV-24 | 0.05875922 | 0.2536587 | small | Beta-lactam |
| TEM-186 | 0.05875922 | 0.2536587 | small | Beta-lactam |
| ErmA | 0.06099167 | 0.25146252 | small | MLS |
| SHV-2 | 0.07063498 | 0.24267781 | small | Beta-lactam |
| tetW | 0.07590653 | 0.23828545 | small | Tetracycline |
| SHV-33 | 0.08441156 | 0.23169691 | small | Beta-lactam |
| dfrA14 | 0.10028571 | 0.22071601 | small | Diaminopyrimidine |
| SHV-16 | 0.10372959 | 0.21851984 | small | Beta-lactam |
| SHV-60 | 0.10372959 | 0.21851984 | small | Beta-lactam |
| SHV-143 | 0.10726679 | 0.21632366 | small | Beta-lactam |
| ErmQ | 0.11089887 | 0.21412748 | small | MLS |
| SHV-67 | 0.1184538 | 0.20973512 | small | Beta-lactam |
| tet41 | 0.1184538 | 0.20973512 | small | Tetracycline |
| tetS | 0.1184538 | 0.20973512 | small | Tetracycline |
| ErmF | 0.13476894 | 0.2009504 | small | MLS |
| SHV-77 | 0.13476894 | 0.2009504 | small | Beta-lactam |
| mphC | 0.13910743 | 0.19875422 | small | Macrolide |
| QnrS1 | 0.13910743 | 0.19875422 | small | Fluoroquinolone |
| SHV-161 | 0.13910743 | 0.19875422 | small | Beta-lactam |
| SHV-8 | 0.13910743 | 0.19875422 | small | Beta-lactam |
| sul1 | 0.15276899 | 0.19216568 | small | Sulfonamides |
| tet32 | 0.16242874 | 0.18777333 | small | Tetracycline |
| vgaC | 0.16492142 | 0.18667204 | small | LS |
| SHV-120 | 0.17254234 | 0.18338097 | small | Beta-lactam |
| SHV-27 | 0.18311968 | 0.17898861 | small | Beta-lactam |
| ErmB | 0.20581847 | 0.17016025 | small | MLS |
| mphA | 0.21772409 | 0.16581153 | small | Macrolide |
| SHV-1 | 0.22392099 | 0.16361535 | small | Beta-lactam |
| SHV-57 | 0.23669089 | 0.159223 | small | Beta-lactam |
| APH(3'')-Ib | 0.2432654 | 0.15702682 | small | Aminoglycoside |
| lsaC | 0.24660843 | 0.15592606 | small | LS |
| SHV-49 | 0.27084228 | 0.1482421 | small | Beta-lactam |
| OXY-1-4 | 0.3488728 | 0.12628031 | small | Beta-lactam |
| ErmX | 0.39277017 | 0.11529941 | small | MLS |
| dfrG | 0.40657373 | 0.11200323 | small | Diaminopyrimidine |
| SHV-148 | 0.43985924 | 0.10431851 | small | Beta-lactam |
| APH(6)-Id | 0.56514622 | 0.07796436 | small | Aminoglycoside |
| tetO | 0.56514622 | 0.07796436 | small | Tetracycline |
| tetB(P) | 0.5763772 | 0.07575523 | small | Tetracycline |
| ANT(6)-Ia | 0.64539304 | 0.06259111 | small | Aminoglycoside |
| mel | 0.66319074 | 0.05929583 | small | MS |
| oqxB | 0.81167757 | 0.03293706 | small | Multidrug |
| mefA | 0.82441924 | 0.03074598 | small | MS |
| tetA(P) | 0.82441924 | 0.03074598 | small | Tetracycline |
| TEM-4 | 0.94758578 | 0.00988281 | small | Beta-lactam |
| oqxA | 0.97377873 | 0.00549045 | small | Multidrug |

**ST 5. Differential Abundance of the Meconium Resistome based on Neonatal Antibiotic Treatment using Wilcoxon Signed Rank test. (n=56 samples). ARGs are arranged in order of effect size (greatest to least).**

| **ARG** | **p-value** | **Effect Size** | **Effect Magnitude** | **ARG (Drug Class)** |
| --- | --- | --- | --- | --- |
| TEM-143 | 0.05093136 | 0.38786724 | moderate | Beta-lactam |
| ErmC | 0.06441194 | 0.36771829 | moderate | MLS |
| SHV-27 | 0.08071058 | 0.34756934 | moderate | Beta-lactam |
| TEM-4 | 0.09003598 | 0.33749487 | moderate | Beta-lactam |
| APH(6)-Id | 0.11127957 | 0.31734592 | moderate | Aminoglycoside |
| sul2 | 0.11127957 | 0.31734592 | moderate | Sulfonamides |
| TEM-206 | 0.11127957 | 0.31734592 | moderate | Beta-lactam |
| TEM-28 | 0.11127957 | 0.31734592 | moderate | Beta-lactam |
| TEM-70 | 0.11127957 | 0.31734592 | moderate | Beta-lactam |
| aadA5 | 0.18167403 | 0.26697355 | small | Aminoglycoside |
| APH(3')-IIb | 0.19905503 | 0.25689908 | small | Aminoglycoside |
| catB7 | 0.19905503 | 0.25689908 | small | Phenicol |
| FosA | 0.19905503 | 0.25689908 | small | Phosphonic |
| OXA-50 | 0.19905503 | 0.25689908 | small | Beta-lactam |
| OXY-1-1 | 0.19905503 | 0.25689908 | small | Beta-lactam |
| OXY-1-2 | 0.19905503 | 0.25689908 | small | Beta-lactam |
| OXY-1-6 | 0.19905503 | 0.25689908 | small | Beta-lactam |
| QnrB47 | 0.19905503 | 0.25689908 | small | Fluoroquinolone |
| QnrB50 | 0.19905503 | 0.25689908 | small | Fluoroquinolone |
| SHV-45 | 0.19905503 | 0.25689908 | small | Beta-lactam |
| SHV-51 | 0.19905503 | 0.25689908 | small | Beta-lactam |
| SHV-71 | 0.19905503 | 0.25689908 | small | Beta-lactam |
| TEM-57 | 0.21762109 | 0.24682461 | small | Beta-lactam |
| TEM-95 | 0.21762109 | 0.24682461 | small | Beta-lactam |
| tetD | 0.21762109 | 0.24682461 | small | Tetracycline |
| tetK | 0.25841781 | 0.22667566 | small | Tetracycline |
| APH(3'')-Ib | 0.28069085 | 0.21660119 | small | Aminoglycoside |
| mecA | 0.28069085 | 0.21660119 | small | Beta-lactam |
| oqxA | 0.28069085 | 0.21660119 | small | Multidrug |
| CfxA3 | 0.30423273 | 0.20652671 | small | Beta-lactam |
| CfxA4 | 0.30423273 | 0.20652671 | small | Beta-lactam |
| SHV-40 | 0.30423273 | 0.20652671 | small | Beta-lactam |
| SHV-75 | 0.30423273 | 0.20652671 | small | Beta-lactam |
| SHV-85 | 0.30423273 | 0.20652671 | small | Beta-lactam |
| SHV-89 | 0.30423273 | 0.20652671 | small | Beta-lactam |
| TEM-122 | 0.30423273 | 0.20652671 | small | Beta-lactam |
| TEM-141 | 0.30423273 | 0.20652671 | small | Beta-lactam |
| TEM-168 | 0.30423273 | 0.20652671 | small | Beta-lactam |
| TEM-186 | 0.30423273 | 0.20652671 | small | Beta-lactam |
| TEM-208 | 0.30423273 | 0.20652671 | small | Beta-lactam |
| TEM-30 | 0.30423273 | 0.20652671 | small | Beta-lactam |
| TEM-8 | 0.30423273 | 0.20652671 | small | Beta-lactam |
| TEM-82 | 0.30423273 | 0.20652671 | small | Beta-lactam |
| TEM-96 | 0.30423273 | 0.20652671 | small | Beta-lactam |
| tetQ | 0.30423273 | 0.20652671 | small | Tetracycline |
| dfrG | 0.32905018 | 0.19645224 | small | Diaminopyrimidine |
| mel | 0.32905018 | 0.19645224 | small | MS |
| OXA-15 | 0.35514337 | 0.18637776 | small | Beta-lactam |
| TEM-198 | 0.3825056 | 0.17630329 | small | Beta-lactam |
| mphC | 0.44097426 | 0.15615434 | small | Macrolide |
| ErmA | 0.47203055 | 0.14607987 | small | MLS |
| oqxB | 0.47248595 | 0.14592976 | small | Multidrug |
| AAC(6')-Ic | 0.50425539 | 0.1360054 | small | Aminoglycoside |
| AAC(6')-Ii | 0.50425539 | 0.1360054 | small | Aminoglycoside |
| aadA24 | 0.50425539 | 0.1360054 | small | Aminoglycoside |
| ACT-15 | 0.50425539 | 0.1360054 | small | Beta-lactam |
| ACT-5 | 0.50425539 | 0.1360054 | small | Beta-lactam |
| ANT(6)-Ia | 0.50425539 | 0.1360054 | small | Aminoglycoside |
| ANT(6)-Ib | 0.50425539 | 0.1360054 | small | Aminoglycoside |
| APH(3')-Ia | 0.50425539 | 0.1360054 | small | Aminoglycoside |
| CfxA2 | 0.50425539 | 0.1360054 | small | Beta-lactam |
| CTX-M-101 | 0.50425539 | 0.1360054 | small | Beta-lactam |
| CTX-M-139 | 0.50425539 | 0.1360054 | small | Beta-lactam |
| CTX-M-55 | 0.50425539 | 0.1360054 | small | Beta-lactam |
| dfrA1 | 0.50425539 | 0.1360054 | small | Diaminopyrimidine |
| dfrA14 | 0.50425539 | 0.1360054 | small | Diaminopyrimidine |
| Erm(33) | 0.50425539 | 0.1360054 | small | MLS |
| ErmF | 0.50425539 | 0.1360054 | small | MLS |
| ErmG | 0.50425539 | 0.1360054 | small | MLS |
| ErmQ | 0.50425539 | 0.1360054 | small | MLS |
| ErmT | 0.50425539 | 0.1360054 | small | MLS |
| floR | 0.50425539 | 0.1360054 | small | Phenicol |
| OXA-347 | 0.50425539 | 0.1360054 | small | Beta-lactam |
| OXY-1-3 | 0.50425539 | 0.1360054 | small | Beta-lactam |
| OXY-1-4 | 0.50425539 | 0.1360054 | small | Beta-lactam |
| OXY-2-7 | 0.50425539 | 0.1360054 | small | Beta-lactam |
| OXY-2-8 | 0.50425539 | 0.1360054 | small | Beta-lactam |
| OXY-4-1 | 0.50425539 | 0.1360054 | small | Beta-lactam |
| OXY-5-2 | 0.50425539 | 0.1360054 | small | Beta-lactam |
| QnrB60 | 0.50425539 | 0.1360054 | small | Fluoroquinolone |
| QnrS1 | 0.50425539 | 0.1360054 | small | Fluoroquinolone |
| QnrS3 | 0.50425539 | 0.1360054 | small | Fluoroquinolone |
| QnrS4 | 0.50425539 | 0.1360054 | small | Fluoroquinolone |
| SHV-1 | 0.50425539 | 0.1360054 | small | Beta-lactam |
| SHV-101 | 0.50425539 | 0.1360054 | small | Beta-lactam |
| SHV-106 | 0.50425539 | 0.1360054 | small | Beta-lactam |
| SHV-11 | 0.50425539 | 0.1360054 | small | Beta-lactam |
| SHV-143 | 0.50425539 | 0.1360054 | small | Beta-lactam |
| SHV-147 | 0.50425539 | 0.1360054 | small | Beta-lactam |
| SHV-148 | 0.50425539 | 0.1360054 | small | Beta-lactam |
| SHV-150 | 0.50425539 | 0.1360054 | small | Beta-lactam |
| SHV-153 | 0.50425539 | 0.1360054 | small | Beta-lactam |
| SHV-16 | 0.50425539 | 0.1360054 | small | Beta-lactam |
| SHV-164 | 0.50425539 | 0.1360054 | small | Beta-lactam |
| SHV-165 | 0.50425539 | 0.1360054 | small | Beta-lactam |
| SHV-178 | 0.50425539 | 0.1360054 | small | Beta-lactam |
| SHV-28 | 0.50425539 | 0.1360054 | small | Beta-lactam |
| SHV-48 | 0.50425539 | 0.1360054 | small | Beta-lactam |
| SHV-5 | 0.50425539 | 0.1360054 | small | Beta-lactam |
| SHV-60 | 0.50425539 | 0.1360054 | small | Beta-lactam |
| SHV-61 | 0.50425539 | 0.1360054 | small | Beta-lactam |
| SHV-62 | 0.50425539 | 0.1360054 | small | Beta-lactam |
| SHV-66 | 0.50425539 | 0.1360054 | small | Beta-lactam |
| SHV-67 | 0.50425539 | 0.1360054 | small | Beta-lactam |
| SHV-78 | 0.50425539 | 0.1360054 | small | Beta-lactam |
| SHV-79 | 0.50425539 | 0.1360054 | small | Beta-lactam |
| SHV-98 | 0.50425539 | 0.1360054 | small | Beta-lactam |
| TEM-10 | 0.50425539 | 0.1360054 | small | Beta-lactam |
| TEM-104 | 0.50425539 | 0.1360054 | small | Beta-lactam |
| TEM-106 | 0.50425539 | 0.1360054 | small | Beta-lactam |
| TEM-166 | 0.50425539 | 0.1360054 | small | Beta-lactam |
| TEM-33 | 0.50425539 | 0.1360054 | small | Beta-lactam |
| tet38 | 0.50425539 | 0.1360054 | small | Tetracycline |
| tet41 | 0.50425539 | 0.1360054 | small | Tetracycline |
| tetA | 0.50425539 | 0.1360054 | small | Tetracycline |
| tetB | 0.50425539 | 0.1360054 | small | Tetracycline |
| tetJ | 0.50425539 | 0.1360054 | small | Tetracycline |
| tetL | 0.50425539 | 0.1360054 | small | Tetracycline |
| tetS | 0.50425539 | 0.1360054 | small | Tetracycline |
| tetX | 0.50425539 | 0.1360054 | small | Tetracycline |
| SHV-110 | 0.53760465 | 0.12593092 | small | Beta-lactam |
| TEM-135 | 0.53760465 | 0.12593092 | small | Beta-lactam |
| mphA | 0.57202661 | 0.11585645 | small | Macrolide |
| dfrA17 | 0.60746218 | 0.10578197 | small | Diaminopyrimidine |
| tetM | 0.60746218 | 0.10578197 | small | Tetracycline |
| fusB | 0.64384509 | 0.0957075 | small | Fusidane |
| lsaC | 0.64384509 | 0.0957075 | small | LS |
| SHV-49 | 0.64384509 | 0.0957075 | small | Beta-lactam |
| ANT(4')-Ib | 0.64418576 | 0.09560915 | small | Aminoglycoside |
| ACT-1 | 0.68110223 | 0.08563303 | small | Beta-lactam |
| ACT-2 | 0.68110223 | 0.08563303 | small | Beta-lactam |
| ACT-7 | 0.68110223 | 0.08563303 | small | Beta-lactam |
| ErmX | 0.68110223 | 0.08563303 | small | MLS |
| SHV-26 | 0.68110223 | 0.08563303 | small | Beta-lactam |
| tetB(P) | 0.68110223 | 0.08563303 | small | Tetracycline |
| tetO | 0.68110223 | 0.08563303 | small | Tetracycline |
| vgaALC | 0.68110223 | 0.08563303 | small | LS |
| DHA-1 | 0.71915409 | 0.07555855 | small | Beta-lactam |
| TEM-127 | 0.71915409 | 0.07555855 | small | Beta-lactam |
| tetA(P) | 0.71915409 | 0.07555855 | small | Tetracycline |
| ErmB | 0.75823638 | 0.06539441 | small | MLS |
| lsaA | 0.83719732 | 0.04533513 | small | LS |
| vgaA | 0.87752315 | 0.03526066 | small | LS |
| SHV-102 | 0.91816942 | 0.02518618 | small | Beta-lactam |
| SHV-120 | 0.91816942 | 0.02518618 | small | Beta-lactam |
| SHV-161 | 0.91816942 | 0.02518618 | small | Beta-lactam |
| SHV-2 | 0.91816942 | 0.02518618 | small | Beta-lactam |
| SHV-24 | 0.91816942 | 0.02518618 | small | Beta-lactam |
| SHV-33 | 0.91816942 | 0.02518618 | small | Beta-lactam |
| SHV-38 | 0.91816942 | 0.02518618 | small | Beta-lactam |
| SHV-57 | 0.91816942 | 0.02518618 | small | Beta-lactam |
| SHV-8 | 0.91816942 | 0.02518618 | small | Beta-lactam |
| SHV-96 | 0.91816942 | 0.02518618 | small | Beta-lactam |
| tet32 | 0.91816942 | 0.02518618 | small | Tetracycline |
| vgaC | 0.91816942 | 0.02518618 | small | LS |
| lnuA | 0.95903074 | 0.01511171 | small | Lincosamide |
| msrC | 0.95903074 | 0.01511171 | small | MS |
| SHV-134 | 0.95903074 | 0.01511171 | small | Beta-lactam |
| SHV-137 | 0.95903074 | 0.01511171 | small | Beta-lactam |
| SHV-42 | 0.95903074 | 0.01511171 | small | Beta-lactam |
| SHV-56 | 0.95903074 | 0.01511171 | small | Beta-lactam |
| SHV-77 | 0.95903074 | 0.01511171 | small | Beta-lactam |
| SHV-81 | 0.95903074 | 0.01511171 | small | Beta-lactam |
| CTX-M-103 | 1 | 0.00503724 | small | Beta-lactam |
| CTX-M-117 | 1 | 0.00503724 | small | Beta-lactam |
| CTX-M-15 | 1 | 0.00503724 | small | Beta-lactam |
| CTX-M-22 | 1 | 0.00503724 | small | Beta-lactam |
| CTX-M-3 | 1 | 0.00503724 | small | Beta-lactam |
| CTX-M-33 | 1 | 0.00503724 | small | Beta-lactam |
| CTX-M-72 | 1 | 0.00503724 | small | Beta-lactam |
| CTX-M-82 | 1 | 0.00503724 | small | Beta-lactam |
| CTX-M-88 | 1 | 0.00503724 | small | Beta-lactam |
| mefA | 1 | 0.00503724 | small | MS |
| sul1 | 1 | 0.00503724 | small | Sulfonamides |
| tetW | 1 | 0.00503724 | small | Tetracycline |

**ST 6. Differential Abundance of the Stool Resistome based on Neonatal Antibiotic Treatment using Wilcoxon Signed Rank test. (n=56 samples). ARGs are arranged in order of effect size (greatest to least).**

| **ARG** | **p-value** | **Effect Size** | **Effect Magnitude** | **ARG (Drug Class)** |
| --- | --- | --- | --- | --- |
| tetB(P) | 0.00448564 | 0.50851052 | large | Tetracycline |
| OXY-2-7 | 0.0068956 | 0.48574139 | moderate | Beta-lactam |
| SHV-110 | 0.0068956 | 0.48574139 | moderate | Beta-lactam |
| SHV-81 | 0.0068956 | 0.48574139 | moderate | Beta-lactam |
| CTX-M-101 | 0.01179331 | 0.45538256 | moderate | Beta-lactam |
| CTX-M-82 | 0.01179331 | 0.45538256 | moderate | Beta-lactam |
| ErmG | 0.01179331 | 0.45538256 | moderate | MLS |
| ErmT | 0.01179331 | 0.45538256 | moderate | MLS |
| OXY-2-8 | 0.01179331 | 0.45538256 | moderate | Beta-lactam |
| tetB | 0.01179331 | 0.45538256 | moderate | Tetracycline |
| APH(3')-Ia | 0.01340299 | 0.44779285 | moderate | Aminoglycoside |
| SHV-153 | 0.01340299 | 0.44779285 | moderate | Beta-lactam |
| TEM-82 | 0.01340299 | 0.44779285 | moderate | Beta-lactam |
| SHV-150 | 0.01519665 | 0.44020314 | moderate | Beta-lactam |
| SHV-56 | 0.01519665 | 0.44020314 | moderate | Beta-lactam |
| SHV-66 | 0.01519665 | 0.44020314 | moderate | Beta-lactam |
| SHV-79 | 0.01519665 | 0.44020314 | moderate | Beta-lactam |
| SHV-85 | 0.01519665 | 0.44020314 | moderate | Beta-lactam |
| tet38 | 0.01519665 | 0.44020314 | moderate | Tetracycline |
| ACT-15 | 0.01718947 | 0.43261343 | moderate | Beta-lactam |
| aadA24 | 0.01939956 | 0.42502372 | moderate | Aminoglycoside |
| ANT(4')-Ib | 0.01939956 | 0.42502372 | moderate | Aminoglycoside |
| dfrA1 | 0.01939956 | 0.42502372 | moderate | Diaminopyrimidine |
| Erm(33) | 0.01939956 | 0.42502372 | moderate | MLS |
| OXY-1-1 | 0.01939956 | 0.42502372 | moderate | Beta-lactam |
| OXY-1-2 | 0.01939956 | 0.42502372 | moderate | Beta-lactam |
| OXY-1-3 | 0.01939956 | 0.42502372 | moderate | Beta-lactam |
| OXY-4-1 | 0.01939956 | 0.42502372 | moderate | Beta-lactam |
| OXY-5-2 | 0.01939956 | 0.42502372 | moderate | Beta-lactam |
| SHV-106 | 0.01939956 | 0.42502372 | moderate | Beta-lactam |
| TEM-106 | 0.01939956 | 0.42502372 | moderate | Beta-lactam |
| TEM-166 | 0.01939956 | 0.42502372 | moderate | Beta-lactam |
| AAC(6')-Ii | 0.02184376 | 0.41743401 | moderate | Aminoglycoside |
| TEM-208 | 0.02184376 | 0.41743401 | moderate | Beta-lactam |
| TEM-33 | 0.02184376 | 0.41743401 | moderate | Beta-lactam |
| ACT-1 | 0.02454202 | 0.4098443 | moderate | Beta-lactam |
| ACT-2 | 0.02751274 | 0.40225459 | moderate | Beta-lactam |
| ACT-7 | 0.02751274 | 0.40225459 | moderate | Beta-lactam |
| APH(3')-IIb | 0.02751274 | 0.40225459 | moderate | Aminoglycoside |
| catB7 | 0.02751274 | 0.40225459 | moderate | Phenicol |
| CfxA4 | 0.02751274 | 0.40225459 | moderate | Beta-lactam |
| CTX-M-103 | 0.02751274 | 0.40225459 | moderate | Beta-lactam |
| CTX-M-22 | 0.02751274 | 0.40225459 | moderate | Beta-lactam |
| CTX-M-3 | 0.02751274 | 0.40225459 | moderate | Beta-lactam |
| CTX-M-33 | 0.02751274 | 0.40225459 | moderate | Beta-lactam |
| CTX-M-72 | 0.02751274 | 0.40225459 | moderate | Beta-lactam |
| CTX-M-88 | 0.02751274 | 0.40225459 | moderate | Beta-lactam |
| FosA | 0.02751274 | 0.40225459 | moderate | Phosphonic |
| fusB | 0.02751274 | 0.40225459 | moderate | Fusidane |
| msrC | 0.02751274 | 0.40225459 | moderate | MS |
| OXA-15 | 0.02751274 | 0.40225459 | moderate | Beta-lactam |
| OXA-50 | 0.02751274 | 0.40225459 | moderate | Beta-lactam |
| OXY-1-6 | 0.02751274 | 0.40225459 | moderate | Beta-lactam |
| QnrB47 | 0.02751274 | 0.40225459 | moderate | Fluoroquinolone |
| QnrB50 | 0.02751274 | 0.40225459 | moderate | Fluoroquinolone |
| SHV-1 | 0.02751274 | 0.40225459 | moderate | Beta-lactam |
| SHV-102 | 0.02751274 | 0.40225459 | moderate | Beta-lactam |
| SHV-134 | 0.02751274 | 0.40225459 | moderate | Beta-lactam |
| SHV-38 | 0.02751274 | 0.40225459 | moderate | Beta-lactam |
| SHV-40 | 0.02751274 | 0.40225459 | moderate | Beta-lactam |
| SHV-42 | 0.02751274 | 0.40225459 | moderate | Beta-lactam |
| SHV-51 | 0.02751274 | 0.40225459 | moderate | Beta-lactam |
| SHV-71 | 0.02751274 | 0.40225459 | moderate | Beta-lactam |
| SHV-89 | 0.02751274 | 0.40225459 | moderate | Beta-lactam |
| TEM-122 | 0.02751274 | 0.40225459 | moderate | Beta-lactam |
| TEM-127 | 0.02751274 | 0.40225459 | moderate | Beta-lactam |
| TEM-135 | 0.02751274 | 0.40225459 | moderate | Beta-lactam |
| TEM-141 | 0.02751274 | 0.40225459 | moderate | Beta-lactam |
| TEM-168 | 0.02751274 | 0.40225459 | moderate | Beta-lactam |
| TEM-198 | 0.02751274 | 0.40225459 | moderate | Beta-lactam |
| TEM-206 | 0.02751274 | 0.40225459 | moderate | Beta-lactam |
| TEM-28 | 0.02751274 | 0.40225459 | moderate | Beta-lactam |
| TEM-30 | 0.02751274 | 0.40225459 | moderate | Beta-lactam |
| TEM-4 | 0.02751274 | 0.40225459 | moderate | Beta-lactam |
| TEM-57 | 0.02751274 | 0.40225459 | moderate | Beta-lactam |
| TEM-70 | 0.02751274 | 0.40225459 | moderate | Beta-lactam |
| TEM-8 | 0.02751274 | 0.40225459 | moderate | Beta-lactam |
| TEM-95 | 0.02751274 | 0.40225459 | moderate | Beta-lactam |
| TEM-96 | 0.02751274 | 0.40225459 | moderate | Beta-lactam |
| tet32 | 0.02751274 | 0.40225459 | moderate | Tetracycline |
| tetD | 0.02751274 | 0.40225459 | moderate | Tetracycline |
| tetK | 0.02751274 | 0.40225459 | moderate | Tetracycline |
| vgaA | 0.02751274 | 0.40225459 | moderate | LS |
| OXY-1-4 | 0.03077766 | 0.39466488 | moderate | Beta-lactam |
| tet41 | 0.04254753 | 0.37189575 | moderate | Tetracycline |
| SHV-164 | 0.04720531 | 0.36430604 | moderate | Beta-lactam |
| SHV-67 | 0.04720531 | 0.36430604 | moderate | Beta-lactam |
| tetS | 0.04720531 | 0.36430604 | moderate | Tetracycline |
| SHV-27 | 0.05226822 | 0.35671634 | moderate | Beta-lactam |
| APH(3'')-Ib | 0.05776204 | 0.34912663 | moderate | Aminoglycoside |
| mphA | 0.0701369 | 0.33394721 | moderate | Macrolide |
| sul2 | 0.0701369 | 0.33394721 | moderate | Sulfonamides |
| ANT(6)-Ia | 0.08452745 | 0.31876779 | moderate | Aminoglycoside |
| ANT(6)-Ib | 0.08452745 | 0.31876779 | moderate | Aminoglycoside |
| SHV-137 | 0.08452745 | 0.31876779 | moderate | Beta-lactam |
| SHV-48 | 0.08452745 | 0.31876779 | moderate | Beta-lactam |
| SHV-60 | 0.08452745 | 0.31876779 | moderate | Beta-lactam |
| SHV-75 | 0.08452745 | 0.31876779 | moderate | Beta-lactam |
| tetA | 0.08452745 | 0.31876779 | moderate | Tetracycline |
| ACT-5 | 0.09253992 | 0.31117808 | moderate | Beta-lactam |
| dfrA14 | 0.09253992 | 0.31117808 | moderate | Diaminopyrimidine |
| ErmA | 0.09253992 | 0.31117808 | moderate | MLS |
| SHV-143 | 0.09253992 | 0.31117808 | moderate | Beta-lactam |
| SHV-45 | 0.09253992 | 0.31117808 | moderate | Beta-lactam |
| SHV-165 | 0.10113124 | 0.30358837 | moderate | Beta-lactam |
| SHV-77 | 0.10113124 | 0.30358837 | moderate | Beta-lactam |
| TEM-143 | 0.10113124 | 0.30358837 | moderate | Beta-lactam |
| TEM-186 | 0.10113124 | 0.30358837 | moderate | Beta-lactam |
| aadA5 | 0.11032328 | 0.29599866 | small | Aminoglycoside |
| AAC(6')-Ic | 0.1201417 | 0.28840895 | small | Aminoglycoside |
| QnrB60 | 0.1201417 | 0.28840895 | small | Fluoroquinolone |
| SHV-101 | 0.1201417 | 0.28840895 | small | Beta-lactam |
| SHV-11 | 0.1201417 | 0.28840895 | small | Beta-lactam |
| SHV-178 | 0.1201417 | 0.28840895 | small | Beta-lactam |
| SHV-26 | 0.1201417 | 0.28840895 | small | Beta-lactam |
| SHV-5 | 0.1201417 | 0.28840895 | small | Beta-lactam |
| SHV-61 | 0.1201417 | 0.28840895 | small | Beta-lactam |
| SHV-62 | 0.1201417 | 0.28840895 | small | Beta-lactam |
| SHV-78 | 0.1201417 | 0.28840895 | small | Beta-lactam |
| SHV-96 | 0.1201417 | 0.28840895 | small | Beta-lactam |
| TEM-104 | 0.1201417 | 0.28840895 | small | Beta-lactam |
| vgaALC | 0.1201417 | 0.28840895 | small | LS |
| CfxA2 | 0.13060706 | 0.28081924 | small | Beta-lactam |
| CfxA3 | 0.13060706 | 0.28081924 | small | Beta-lactam |
| CTX-M-117 | 0.13060706 | 0.28081924 | small | Beta-lactam |
| CTX-M-139 | 0.13060706 | 0.28081924 | small | Beta-lactam |
| CTX-M-15 | 0.13060706 | 0.28081924 | small | Beta-lactam |
| CTX-M-55 | 0.13060706 | 0.28081924 | small | Beta-lactam |
| DHA-1 | 0.13060706 | 0.28081924 | small | Beta-lactam |
| floR | 0.13060706 | 0.28081924 | small | Phenicol |
| lnuA | 0.13060706 | 0.28081924 | small | Lincosamide |
| OXA-347 | 0.13060706 | 0.28081924 | small | Beta-lactam |
| QnrS3 | 0.13060706 | 0.28081924 | small | Fluoroquinolone |
| QnrS4 | 0.13060706 | 0.28081924 | small | Fluoroquinolone |
| SHV-147 | 0.13060706 | 0.28081924 | small | Beta-lactam |
| SHV-28 | 0.13060706 | 0.28081924 | small | Beta-lactam |
| SHV-98 | 0.13060706 | 0.28081924 | small | Beta-lactam |
| TEM-10 | 0.13060706 | 0.28081924 | small | Beta-lactam |
| tetJ | 0.13060706 | 0.28081924 | small | Tetracycline |
| tetQ | 0.13060706 | 0.28081924 | small | Tetracycline |
| tetX | 0.13060706 | 0.28081924 | small | Tetracycline |
| SHV-161 | 0.14174362 | 0.27322953 | small | Beta-lactam |
| SHV-120 | 0.15356988 | 0.26563982 | small | Beta-lactam |
| tetA(P) | 0.16610811 | 0.25805012 | small | Tetracycline |
| dfrA17 | 0.19338774 | 0.2428707 | small | Diaminopyrimidine |
| tetM | 0.19338774 | 0.2428707 | small | Tetracycline |
| lsaC | 0.22371126 | 0.22769128 | small | LS |
| oqxB | 0.22371126 | 0.22769128 | small | Multidrug |
| tetL | 0.22371126 | 0.22769128 | small | Tetracycline |
| APH(6)-Id | 0.25717871 | 0.21251186 | small | Aminoglycoside |
| oqxA | 0.25717871 | 0.21251186 | small | Multidrug |
| SHV-16 | 0.27511286 | 0.20492215 | small | Beta-lactam |
| ErmQ | 0.29385662 | 0.19733244 | small | MLS |
| SHV-24 | 0.35494423 | 0.17456331 | small | Beta-lactam |
| sul1 | 0.35494423 | 0.17456331 | small | Sulfonamides |
| tetW | 0.35494423 | 0.17456331 | small | Tetracycline |
| SHV-2 | 0.37691802 | 0.1669736 | small | Beta-lactam |
| SHV-33 | 0.37691802 | 0.1669736 | small | Beta-lactam |
| ErmF | 0.39968341 | 0.15938389 | small | MLS |
| mphC | 0.39968341 | 0.15938389 | small | Macrolide |
| QnrS1 | 0.39968341 | 0.15938389 | small | Fluoroquinolone |
| mecA | 0.42323246 | 0.15179419 | small | Beta-lactam |
| mel | 0.42323246 | 0.15179419 | small | MS |
| ErmX | 0.44754743 | 0.14420448 | small | MLS |
| vgaC | 0.47261462 | 0.13661477 | small | LS |
| SHV-49 | 0.49841032 | 0.12902506 | small | Beta-lactam |
| dfrG | 0.52491511 | 0.12143535 | small | Diaminopyrimidine |
| tetO | 0.52491511 | 0.12143535 | small | Tetracycline |
| ErmB | 0.55209936 | 0.11384564 | small | MLS |
| mefA | 0.55209936 | 0.11384564 | small | MS |
| SHV-57 | 0.57993802 | 0.10625593 | small | Beta-lactam |
| ErmC | 0.6670373 | 0.0834868 | small | MLS |
| lsaA | 0.69714515 | 0.07589709 | small | LS |
| SHV-8 | 0.69714515 | 0.07589709 | small | Beta-lactam |
| SHV-148 | 0.82182519 | 0.04553826 | small | Beta-lactam |

**ST 7. Differential Abundance of the Stool Resistome based on ROM duration >18 hours using Wilcoxon Signed Rank test. (n=56 samples). ARGs are arranged in order of effect size (greatest to least).**

| **ARG** | **p-value** | **Effect Size** | **Effect Magnitude** | **ARG (Drug Class)** |
| --- | --- | --- | --- | --- |
| mphC | 0.00194282 | 0.54618914 | large | Macrolide |
| dfrA14 | 0.00231712 | 0.53815695 | large | Diaminopyrimidine |
| TEM-186 | 0.00231712 | 0.53815695 | large | Beta-lactam |
| DHA-1 | 0.00275334 | 0.53012476 | large | Beta-lactam |
| floR | 0.00275334 | 0.53012476 | large | Phenicol |
| SHV-28 | 0.00275334 | 0.53012476 | large | Beta-lactam |
| tetJ | 0.00275334 | 0.53012476 | large | Tetracycline |
| tetL | 0.00275334 | 0.53012476 | large | Tetracycline |
| oqxB | 0.00384283 | 0.51406037 | large | Multidrug |
| AAC(6')-Ic | 0.00616914 | 0.48996379 | moderate | Aminoglycoside |
| ErmG | 0.00616914 | 0.48996379 | moderate | MLS |
| ErmT | 0.00616914 | 0.48996379 | moderate | MLS |
| OXY-2-8 | 0.00616914 | 0.48996379 | moderate | Beta-lactam |
| QnrB60 | 0.00616914 | 0.48996379 | moderate | Fluoroquinolone |
| vgaALC | 0.00616914 | 0.48996379 | moderate | LS |
| ACT-5 | 0.00717597 | 0.4819316 | moderate | Beta-lactam |
| APH(3')-Ia | 0.00717597 | 0.4819316 | moderate | Aminoglycoside |
| SHV-153 | 0.00717597 | 0.4819316 | moderate | Beta-lactam |
| SHV-165 | 0.00717597 | 0.4819316 | moderate | Beta-lactam |
| TEM-82 | 0.00717597 | 0.4819316 | moderate | Beta-lactam |
| aadA24 | 0.00831925 | 0.4738994 | moderate | Aminoglycoside |
| ANT(4')-Ib | 0.00831925 | 0.4738994 | moderate | Aminoglycoside |
| ANT(6)-Ib | 0.00831925 | 0.4738994 | moderate | Aminoglycoside |
| dfrA1 | 0.00831925 | 0.4738994 | moderate | Diaminopyrimidine |
| Erm(33) | 0.00831925 | 0.4738994 | moderate | MLS |
| OXY-1-1 | 0.00831925 | 0.4738994 | moderate | Beta-lactam |
| OXY-1-2 | 0.00831925 | 0.4738994 | moderate | Beta-lactam |
| OXY-1-3 | 0.00831925 | 0.4738994 | moderate | Beta-lactam |
| OXY-4-1 | 0.00831925 | 0.4738994 | moderate | Beta-lactam |
| OXY-5-2 | 0.00831925 | 0.4738994 | moderate | Beta-lactam |
| SHV-137 | 0.00831925 | 0.4738994 | moderate | Beta-lactam |
| SHV-48 | 0.00831925 | 0.4738994 | moderate | Beta-lactam |
| SHV-75 | 0.00831925 | 0.4738994 | moderate | Beta-lactam |
| TEM-106 | 0.00831925 | 0.4738994 | moderate | Beta-lactam |
| TEM-166 | 0.00831925 | 0.4738994 | moderate | Beta-lactam |
| tetA | 0.00831925 | 0.4738994 | moderate | Tetracycline |
| AAC(6')-Ii | 0.00961584 | 0.46586721 | moderate | Aminoglycoside |
| ACT-1 | 0.00961584 | 0.46586721 | moderate | Beta-lactam |
| ACT-15 | 0.00961584 | 0.46586721 | moderate | Beta-lactam |
| SHV-164 | 0.00961584 | 0.46586721 | moderate | Beta-lactam |
| TEM-208 | 0.00961584 | 0.46586721 | moderate | Beta-lactam |
| TEM-33 | 0.00961584 | 0.46586721 | moderate | Beta-lactam |
| ACT-2 | 0.01107951 | 0.45783502 | moderate | Beta-lactam |
| ACT-7 | 0.01107951 | 0.45783502 | moderate | Beta-lactam |
| APH(3')-IIb | 0.01107951 | 0.45783502 | moderate | Aminoglycoside |
| catB7 | 0.01107951 | 0.45783502 | moderate | Phenicol |
| CfxA4 | 0.01107951 | 0.45783502 | moderate | Beta-lactam |
| CTX-M-103 | 0.01107951 | 0.45783502 | moderate | Beta-lactam |
| CTX-M-22 | 0.01107951 | 0.45783502 | moderate | Beta-lactam |
| CTX-M-3 | 0.01107951 | 0.45783502 | moderate | Beta-lactam |
| CTX-M-33 | 0.01107951 | 0.45783502 | moderate | Beta-lactam |
| CTX-M-72 | 0.01107951 | 0.45783502 | moderate | Beta-lactam |
| CTX-M-88 | 0.01107951 | 0.45783502 | moderate | Beta-lactam |
| FosA | 0.01107951 | 0.45783502 | moderate | Phosphonic |
| fusB | 0.01107951 | 0.45783502 | moderate | Fusidane |
| msrC | 0.01107951 | 0.45783502 | moderate | MS |
| OXA-15 | 0.01107951 | 0.45783502 | moderate | Beta-lactam |
| OXA-50 | 0.01107951 | 0.45783502 | moderate | Beta-lactam |
| OXY-1-6 | 0.01107951 | 0.45783502 | moderate | Beta-lactam |
| QnrB47 | 0.01107951 | 0.45783502 | moderate | Fluoroquinolone |
| QnrB50 | 0.01107951 | 0.45783502 | moderate | Fluoroquinolone |
| SHV-102 | 0.01107951 | 0.45783502 | moderate | Beta-lactam |
| SHV-134 | 0.01107951 | 0.45783502 | moderate | Beta-lactam |
| SHV-38 | 0.01107951 | 0.45783502 | moderate | Beta-lactam |
| SHV-40 | 0.01107951 | 0.45783502 | moderate | Beta-lactam |
| SHV-42 | 0.01107951 | 0.45783502 | moderate | Beta-lactam |
| SHV-51 | 0.01107951 | 0.45783502 | moderate | Beta-lactam |
| SHV-71 | 0.01107951 | 0.45783502 | moderate | Beta-lactam |
| SHV-89 | 0.01107951 | 0.45783502 | moderate | Beta-lactam |
| TEM-122 | 0.01107951 | 0.45783502 | moderate | Beta-lactam |
| TEM-127 | 0.01107951 | 0.45783502 | moderate | Beta-lactam |
| TEM-135 | 0.01107951 | 0.45783502 | moderate | Beta-lactam |
| TEM-141 | 0.01107951 | 0.45783502 | moderate | Beta-lactam |
| TEM-168 | 0.01107951 | 0.45783502 | moderate | Beta-lactam |
| TEM-198 | 0.01107951 | 0.45783502 | moderate | Beta-lactam |
| TEM-206 | 0.01107951 | 0.45783502 | moderate | Beta-lactam |
| TEM-28 | 0.01107951 | 0.45783502 | moderate | Beta-lactam |
| TEM-30 | 0.01107951 | 0.45783502 | moderate | Beta-lactam |
| TEM-57 | 0.01107951 | 0.45783502 | moderate | Beta-lactam |
| TEM-70 | 0.01107951 | 0.45783502 | moderate | Beta-lactam |
| TEM-8 | 0.01107951 | 0.45783502 | moderate | Beta-lactam |
| TEM-95 | 0.01107951 | 0.45783502 | moderate | Beta-lactam |
| TEM-96 | 0.01107951 | 0.45783502 | moderate | Beta-lactam |
| tetD | 0.01107951 | 0.45783502 | moderate | Tetracycline |
| tetK | 0.01107951 | 0.45783502 | moderate | Tetracycline |
| vgaA | 0.01107951 | 0.45783502 | moderate | LS |
| SHV-106 | 0.03111524 | 0.39357747 | moderate | Beta-lactam |
| OXY-1-4 | 0.03498877 | 0.38554528 | moderate | Beta-lactam |
| QnrS1 | 0.03924658 | 0.37751308 | moderate | Fluoroquinolone |
| dfrA17 | 0.04391937 | 0.36948089 | moderate | Diaminopyrimidine |
| SHV-2 | 0.04391937 | 0.36948089 | moderate | Beta-lactam |
| tetM | 0.04391937 | 0.36948089 | moderate | Tetracycline |
| SHV-150 | 0.06069213 | 0.34538431 | moderate | Beta-lactam |
| SHV-56 | 0.06069213 | 0.34538431 | moderate | Beta-lactam |
| SHV-66 | 0.06069213 | 0.34538431 | moderate | Beta-lactam |
| SHV-67 | 0.06069213 | 0.34538431 | moderate | Beta-lactam |
| SHV-79 | 0.06069213 | 0.34538431 | moderate | Beta-lactam |
| SHV-85 | 0.06069213 | 0.34538431 | moderate | Beta-lactam |
| tet38 | 0.06069213 | 0.34538431 | moderate | Tetracycline |
| tetS | 0.06069213 | 0.34538431 | moderate | Tetracycline |
| ErmQ | 0.06729948 | 0.33735212 | moderate | MLS |
| tet41 | 0.06729948 | 0.33735212 | moderate | Tetracycline |
| SHV-16 | 0.07446061 | 0.32931992 | moderate | Beta-lactam |
| SHV-60 | 0.07446061 | 0.32931992 | moderate | Beta-lactam |
| sul2 | 0.07446061 | 0.32931992 | moderate | Sulfonamides |
| CTX-M-101 | 0.08220944 | 0.32128773 | moderate | Beta-lactam |
| CTX-M-82 | 0.08220944 | 0.32128773 | moderate | Beta-lactam |
| dfrG | 0.08220944 | 0.32128773 | moderate | Diaminopyrimidine |
| ErmA | 0.08220944 | 0.32128773 | moderate | MLS |
| SHV-143 | 0.08220944 | 0.32128773 | moderate | Beta-lactam |
| tetB | 0.08220944 | 0.32128773 | moderate | Tetracycline |
| TEM-143 | 0.09057017 | 0.31325554 | moderate | Beta-lactam |
| CfxA2 | 0.09957652 | 0.30522334 | moderate | Beta-lactam |
| CfxA3 | 0.09957652 | 0.30522334 | moderate | Beta-lactam |
| CTX-M-117 | 0.09957652 | 0.30522334 | moderate | Beta-lactam |
| CTX-M-139 | 0.09957652 | 0.30522334 | moderate | Beta-lactam |
| CTX-M-15 | 0.09957652 | 0.30522334 | moderate | Beta-lactam |
| CTX-M-55 | 0.09957652 | 0.30522334 | moderate | Beta-lactam |
| OXA-347 | 0.09957652 | 0.30522334 | moderate | Beta-lactam |
| OXY-2-7 | 0.09957652 | 0.30522334 | moderate | Beta-lactam |
| SHV-101 | 0.09957652 | 0.30522334 | moderate | Beta-lactam |
| SHV-11 | 0.09957652 | 0.30522334 | moderate | Beta-lactam |
| SHV-110 | 0.09957652 | 0.30522334 | moderate | Beta-lactam |
| SHV-178 | 0.09957652 | 0.30522334 | moderate | Beta-lactam |
| SHV-26 | 0.09957652 | 0.30522334 | moderate | Beta-lactam |
| SHV-27 | 0.09957652 | 0.30522334 | moderate | Beta-lactam |
| SHV-5 | 0.09957652 | 0.30522334 | moderate | Beta-lactam |
| SHV-61 | 0.09957652 | 0.30522334 | moderate | Beta-lactam |
| SHV-62 | 0.09957652 | 0.30522334 | moderate | Beta-lactam |
| SHV-78 | 0.09957652 | 0.30522334 | moderate | Beta-lactam |
| SHV-81 | 0.09957652 | 0.30522334 | moderate | Beta-lactam |
| SHV-96 | 0.09957652 | 0.30522334 | moderate | Beta-lactam |
| TEM-104 | 0.09957652 | 0.30522334 | moderate | Beta-lactam |
| tetQ | 0.09957652 | 0.30522334 | moderate | Tetracycline |
| tetX | 0.09957652 | 0.30522334 | moderate | Tetracycline |
| lnuA | 0.1092518 | 0.29719115 | small | Lincosamide |
| QnrS3 | 0.1092518 | 0.29719115 | small | Fluoroquinolone |
| QnrS4 | 0.1092518 | 0.29719115 | small | Fluoroquinolone |
| SHV-147 | 0.1092518 | 0.29719115 | small | Beta-lactam |
| SHV-98 | 0.1092518 | 0.29719115 | small | Beta-lactam |
| TEM-10 | 0.1092518 | 0.29719115 | small | Beta-lactam |
| APH(3'')-Ib | 0.13072884 | 0.28112677 | small | Aminoglycoside |
| lsaC | 0.13072884 | 0.28112677 | small | LS |
| SHV-120 | 0.13072884 | 0.28112677 | small | Beta-lactam |
| mefA | 0.15521017 | 0.26506238 | small | MS |
| tetA(P) | 0.15521017 | 0.26506238 | small | Tetracycline |
| SHV-148 | 0.18288405 | 0.24899799 | small | Beta-lactam |
| tetB(P) | 0.18288405 | 0.24899799 | small | Tetracycline |
| mecA | 0.21391123 | 0.23293361 | small | Beta-lactam |
| SHV-45 | 0.21391123 | 0.23293361 | small | Beta-lactam |
| APH(6)-Id | 0.24841871 | 0.21686922 | small | Aminoglycoside |
| vgaC | 0.24841871 | 0.21686922 | small | LS |
| SHV-57 | 0.26700815 | 0.20883703 | small | Beta-lactam |
| SHV-77 | 0.26700815 | 0.20883703 | small | Beta-lactam |
| tet32 | 0.30688472 | 0.19277264 | small | Tetracycline |
| SHV-161 | 0.32817571 | 0.18474045 | small | Beta-lactam |
| SHV-1 | 0.35037107 | 0.17670825 | small | Beta-lactam |
| SHV-8 | 0.35037107 | 0.17670825 | small | Beta-lactam |
| SHV-24 | 0.42227271 | 0.15261167 | small | Beta-lactam |
| tetW | 0.42227271 | 0.15261167 | small | Tetracycline |
| ErmF | 0.44797295 | 0.14457948 | small | MLS |
| SHV-33 | 0.44797295 | 0.14457948 | small | Beta-lactam |
| sul1 | 0.50184738 | 0.12851509 | small | Sulfonamides |
| mel | 0.52996971 | 0.1204829 | small | MS |
| oqxA | 0.52996971 | 0.1204829 | small | Multidrug |
| tetO | 0.52996971 | 0.1204829 | small | Tetracycline |
| ANT(6)-Ia | 0.68111925 | 0.08032193 | small | Aminoglycoside |
| mphA | 0.68111925 | 0.08032193 | small | Macrolide |
| ErmC | 0.71316383 | 0.07228974 | small | MLS |
| SHV-49 | 0.7457092 | 0.06425755 | small | Beta-lactam |
| aadA5 | 0.81207209 | 0.04819316 | small | Aminoglycoside |
| TEM-4 | 0.81207209 | 0.04819316 | small | Beta-lactam |
| ErmX | 0.84577864 | 0.04016097 | small | MLS |
| ErmB | 0.87976465 | 0.03212877 | small | MLS |
| lsaA | 1 | 0 | small | LS |

**ST 8. Differential Abundance of the functional profiling of meconium samples based on antibiotic treatment using the Wilcoxon Signed Rank test. (n=60 samples).**

| **Function** | **p-value** | **Effect Size** | **Effect Magnitude** | **SEED Category** |
| --- | --- | --- | --- | --- |
| Anaerobic module of TCA | 0.002799 | 0.6032 | large | ENERGY |
| Proline Synthesis | 0.005622 | 0.5647 | large | METABOLISM |
| Histidine Biosynthesis | 0.015606 | 0.5005 | large | METABOLISM |
| Hydroxyaromatic decarboxylase family | 0.015606 | 0.5005 | large | METABOLISM |
| L-rhamnonate utilization cluster | 0.015606 | 0.5005 | large | METABOLISM |
| Heme Biosynthesis: protoporphyrin-, coproporphyrin- and siroheme-dependent pathways | 0.0225 | 0.4748 | moderate | METABOLISM |
| D-Galacturonate and D-Glucuronate Utilization | 0.0268 | 0.462 | moderate | METABOLISM |
| tRNA aminoacylation, Ser | 0.031744 | 0.4492 | moderate | PROTEIN PROCESSING |
| Peptidase clustering with DAP | 0.037418 | 0.4363 | moderate | METABOLISM |
| Chitobiose | 0.037418 | 0.4363 | moderate | METABOLISM |
| Sugar-phosphate stress regulation | 0.043879 | 0.4235 | moderate | STRESS RESPONSE, DEFENSE, VIRULENCE |
| Lipoic acid metabolism | 0.059498 | 0.3978 | moderate | METABOLISM |
| D-alanylation of teichoic acid | 0.068817 | 0.385 | moderate | CELL ENVELOPE |
| Fermentations: Mixed acid | 0.068817 | 0.385 | moderate | ENERGY |
| Glutathionylspermidine and Trypanothione | 0.068817 | 0.385 | moderate | STRESS RESPONSE, DEFENSE, VIRULENCE |
| Calvin-Benson cycle | 0.068817 | 0.385 | moderate | METABOLISM |
| Heat shock dnaK gene cluster extended | 0.079231 | 0.3722 | moderate | STRESS RESPONSE, DEFENSE, VIRULENCE |
| TonB-ExbBD energy transducing system | 0.079231 | 0.3722 | moderate | CELL ENVELOPE |
| Fatty Acid Biosynthesis FASII | 0.090842 | 0.3593 | moderate | METABOLISM |
| Poly-gamma-glutamate biosynthesis | 0.090842 | 0.3593 | moderate | METABOLISM |
| Polymyxin resistance, lipid A modifications with 4-amino-4-deoxy-L-arabinose | 0.090842 | 0.3593 | moderate | STRESS RESPONSE, DEFENSE, VIRULENCE |
| Resistance to Ethionamide and Isoniazid | 0.090842 | 0.3593 | moderate | STRESS RESPONSE, DEFENSE, VIRULENCE |
| Macrolides, lincosamides, streptogramins, ketolides, oxazolidinones (MLSKO) resistance: ribosomal protection | 0.103709 | 0.3465 | moderate | STRESS RESPONSE, DEFENSE, VIRULENCE |
| Putrescine utilization | 0.103709 | 0.3465 | moderate | METABOLISM |
| Galactose utilization | 0.117928 | 0.3337 | moderate | METABOLISM |
| Trimethylamine-N-oxide operon | 0.117928 | 0.3337 | moderate | ENERGY |
| Dihydropyrimidine dehydrogenase | 0.133549 | 0.3208 | moderate | METABOLISM |
| Fatty acid degradation | 0.133549 | 0.3208 | moderate | METABOLISM |
| Glycogen metabolism | 0.133549 | 0.3208 | moderate | METABOLISM |
| Phenylalanine and Tyrosine synthesis 1 | 0.133549 | 0.3208 | moderate | METABOLISM |
| Programmed frameshift | 0.133549 | 0.3208 | moderate | PROTEIN PROCESSING |
| Sporulation Cluster | 0.133549 | 0.3208 | moderate | CELLULAR PROCESSES |
| Streptococcal Hyaluronic Acid Capsule | 0.133549 | 0.3208 | moderate | CELL ENVELOPE |
| tRNA aminoacylation, Pro | 0.133549 | 0.3208 | moderate | PROTEIN PROCESSING |
| Dihydroxyacetone kinases | 0.150667 | 0.308 | moderate | ENERGY |
| Inner membrane proteins of MarC family, not involved in antibiotic resistance | 0.150667 | 0.308 | moderate | STRESS RESPONSE, DEFENSE, VIRULENCE |
| tRNA aminoacylation, Leu | 0.150667 | 0.308 | moderate | PROTEIN PROCESSING |
| Chaperones GroEL GroES and Thermosome | 0.150667 | 0.308 | moderate | PROTEIN PROCESSING |
| Glutamine synthetases | 0.169317 | 0.2952 | small | METABOLISM |
| Mevalonate metabolic pathway | 0.169317 | 0.2952 | small | METABOLISM |
| Protection from Reactive Oxygen Species | 0.169317 | 0.2952 | small | STRESS RESPONSE, DEFENSE, VIRULENCE |
| Purine catabolism in Bacillus subtilis | 0.169317 | 0.2952 | small | METABOLISM |
| Arginine biosynthesis | 0.169317 | 0.2952 | small | METABOLISM |
| Ribosome LSU, bacterial | 0.169317 | 0.2952 | small | PROTEIN PROCESSING |
| Urea carboxylase and Allophanate hydrolase cluster | 0.169317 | 0.2952 | small | METABOLISM |
| YoeB-YefM toxin-antitoxin system | 0.169317 | 0.2952 | small | CELLULAR PROCESSES |
| tRNA aminoacylation, Asn | 0.169317 | 0.2952 | small | PROTEIN PROCESSING |
| Biosynthesis of Arabinogalactan in Mycobacteria | 0.169317 | 0.2952 | small | CELL ENVELOPE |
| Carbon storage regulator | 0.169317 | 0.2952 | small | METABOLISM |
| Colicin V and Bacteriocin Production Cluster | 0.169317 | 0.2952 | small | METABOLISM |
| L-fucose dissimilation cluster | 0.189587 | 0.2823 | small | METABOLISM |
| Menaquinone biosynthesis from chorismate via 1,4-dihydroxy-2-naphthoate | 0.189587 | 0.2823 | small | METABOLISM |
| NADH ubiquinone oxidoreductase vs. multi-subunit cation antiporter | 0.189587 | 0.2823 | small | ENERGY |
| Nucleoside triphosphate pyrophosphohydrolase MazG | 0.189587 | 0.2823 | small | CELLULAR PROCESSES |
| Resistance to Daptomycin | 0.189587 | 0.2823 | small | STRESS RESPONSE, DEFENSE, VIRULENCE |
| Arginine biosynthesis via N-acetyl-L-citrulline | 0.189587 | 0.2823 | small | METABOLISM |
| D-gluconate and ketogluconates metabolism | 0.211497 | 0.2695 | small | METABOLISM |
| Fe-S cluster assembly | 0.211497 | 0.2695 | small | METABOLISM |
| Outer membrane | 0.211497 | 0.2695 | small | CELL ENVELOPE |
| Phospholipid and Fatty acid biosynthesis related cluster | 0.211497 | 0.2695 | small | METABOLISM |
| Sulfur transfer pathway CsdAEL | 0.211497 | 0.2695 | small | METABOLISM |
| Xanthine dehydrogenase subunits | 0.211497 | 0.2695 | small | METABOLISM |
| Cadmium resistance | 0.211497 | 0.2695 | small | STRESS RESPONSE, DEFENSE, VIRULENCE |
| Peptide methionine sulfoxide reductase | 0.235117 | 0.2567 | small | PROTEIN PROCESSING |
| Proline, 4-hydroxyproline uptake and utilization | 0.235117 | 0.2567 | small | METABOLISM |
| Proteasome bacterial | 0.235117 | 0.2567 | small | PROTEIN PROCESSING |
| Beta-lactamases Ambler class A | 0.235117 | 0.2567 | small | STRESS RESPONSE, DEFENSE, VIRULENCE |
| Coenzyme PQQ synthesis | 0.235117 | 0.2567 | small | METABOLISM |
| EnvZ and OmpR regulon | 0.260448 | 0.2438 | small | STRESS RESPONSE, DEFENSE, VIRULENCE |
| Siderophore Anthrachelin | 0.260448 | 0.2438 | small | METABOLISM |
| tRNA aminoacylation, Gln | 0.260448 | 0.2438 | small | PROTEIN PROCESSING |
| Beta-lactamases Ambler class C | 0.260448 | 0.2438 | small | STRESS RESPONSE, DEFENSE, VIRULENCE |
| CoA disulfide thiol-disulfide redox system | 0.260448 | 0.2438 | small | STRESS RESPONSE, DEFENSE, VIRULENCE |
| Colanic acid synthesis | 0.260448 | 0.2438 | small | CELL ENVELOPE |
| Glutamate fermentation | 0.287547 | 0.231 | small | METABOLISM |
| One-carbon metabolism by tetrahydropterines | 0.287547 | 0.231 | small | METABOLISM |
| Peptidyl-prolyl cis-trans isomerase | 0.287547 | 0.231 | small | PROTEIN PROCESSING |
| Phenylacetyl-CoA catabolic pathway (core) | 0.287547 | 0.231 | small | METABOLISM |
| Purine nucleotide synthesis regulator | 0.287547 | 0.231 | small | METABOLISM |
| Salmochelin-mediated Iron Acquisition | 0.287547 | 0.231 | small | METABOLISM |
| Selenoprotein O | 0.287547 | 0.231 | small | PROTEIN PROCESSING |
| Tetracycline resistance, ribosomal protection type | 0.287547 | 0.231 | small | STRESS RESPONSE, DEFENSE, VIRULENCE |
| Wall polysaccharide pyruvylation | 0.287547 | 0.231 | small | CELL ENVELOPE |
| Biogenesis of c-type cytochromes | 0.287547 | 0.231 | small | ENERGY |
| Formaldehyde assimilation: Ribulose monophosphate pathway | 0.316386 | 0.2182 | small | METABOLISM |
| Gallic acid utilization | 0.316386 | 0.2182 | small | METABOLISM |
| Glycerol fermentation to 1,3-propanediol | 0.316386 | 0.2182 | small | ENERGY |
| Glycolate, glyoxylate interconversions | 0.316386 | 0.2182 | small | ENERGY |
| Heme O and Heme A biosynthesis | 0.316386 | 0.2182 | small | ENERGY |
| L-galactonate utilization | 0.316386 | 0.2182 | small | METABOLISM |
| Lipoylated proteins | 0.316386 | 0.2182 | small | METABOLISM |
| Pyruvate metabolism II: acetyl-CoA, acetogenesis from pyruvate | 0.316386 | 0.2182 | small | ENERGY |
| Repair of Iron Centers | 0.316386 | 0.2182 | small | STRESS RESPONSE, DEFENSE, VIRULENCE |
| Sucrose to levan conversions | 0.316386 | 0.2182 | small | METABOLISM |
| TCA Cycle | 0.316386 | 0.2182 | small | ENERGY |
| Branched-Chain Amino Acid Biosynthesis | 0.316386 | 0.2182 | small | METABOLISM |
| Cobalamin synthesis, Precorin-2 to Cob(II)yrinate a,c diamide, aerobic | 0.316386 | 0.2182 | small | METABOLISM |
| Cobalamin synthesis, Precorin-2 to Cob(II)yrinate a,c diamide, anaerobic | 0.316386 | 0.2182 | small | METABOLISM |
| Darabinitol utilization | 0.346999 | 0.2053 | small | METABOLISM |
| Dehydrogenase complexes | 0.346999 | 0.2053 | small | ENERGY |
| GMP synthase | 0.346999 | 0.2053 | small | METABOLISM |
| Homogentisate pathway | 0.346999 | 0.2053 | small | METABOLISM |
| Pyrroloquinoline Quinone biosynthesis | 0.346999 | 0.2053 | small | METABOLISM |
| Universal stress protein family | 0.346999 | 0.2053 | small | STRESS RESPONSE, DEFENSE, VIRULENCE |
| V-Type ATP synthase | 0.346999 | 0.2053 | small | ENERGY |
| Bicyclomycin resistance cluster | 0.346999 | 0.2053 | small | STRESS RESPONSE, DEFENSE, VIRULENCE |
| Cold shock proteins of CSP family | 0.346999 | 0.2053 | small | STRESS RESPONSE, DEFENSE, VIRULENCE |
| Cysteine dioxygenase containing cluster 1 | 0.379339 | 0.1925 | small | METABOLISM |
| Acyl-CoA thioesterase II | 0.379339 | 0.1925 | small | METABOLISM |
| Fatty acid synthesis | 0.379339 | 0.1925 | small | METABOLISM |
| Alpha-acetolactate operon | 0.379339 | 0.1925 | small | METABOLISM |
| Listeria surface proteins: Internalin-like proteins | 0.379339 | 0.1925 | small | STRESS RESPONSE, DEFENSE, VIRULENCE |
| Lysine DAP biosynthetic pathway | 0.379339 | 0.1925 | small | METABOLISM |
| MazEF toxin-antitoxing (programmed cell death) system | 0.379339 | 0.1925 | small | CELLULAR PROCESSES |
| Anaerobic dimethyl sulfoxide reductase (EC 1.8.5.3) | 0.379339 | 0.1925 | small | ENERGY |
| Phage shock protein operon | 0.379339 | 0.1925 | small | STRESS RESPONSE, DEFENSE, VIRULENCE |
| Antibiotic targets in metabolic pathways | 0.379339 | 0.1925 | small | STRESS RESPONSE, DEFENSE, VIRULENCE |
| Aspartate to Threonine Module | 0.379339 | 0.1925 | small | METABOLISM |
| Tolerance to colicin E2 and CreBC signal transduction system | 0.379339 | 0.1925 | small | METABOLISM |
| Bacterial Sphingolipids | 0.379339 | 0.1925 | small | METABOLISM |
| Chorismate Synthesis | 0.379339 | 0.1925 | small | METABOLISM |
| CtsR and MscAB regulation of protein degradation | 0.379339 | 0.1925 | small | PROTEIN PROCESSING |
| Actinobacterial signal transduction system MtrAB-LpqB | 0.413411 | 0.1797 | small | STRESS RESPONSE, DEFENSE, VIRULENCE |
| Glycerolipid and Glycerophospholipid Metabolism in Bacteria | 0.413411 | 0.1797 | small | METABOLISM |
| Lipid-linked oligosaccharide synthesis related cluster | 0.413411 | 0.1797 | small | CELL ENVELOPE |
| Riboflavin, FMN and FAD metabolism with fusion events | 0.413411 | 0.1797 | small | METABOLISM |
| Siderophore Enterobactin | 0.413411 | 0.1797 | small | METABOLISM |
| Urea cycle | 0.413411 | 0.1797 | small | METABOLISM |
| Uridine to beta-Alanine and Thymine to 3-aminoisobutanoate module | 0.413411 | 0.1797 | small | METABOLISM |
| tRNA aminoacylation, Phe | 0.413411 | 0.1797 | small | PROTEIN PROCESSING |
| Biofilm formation in Staphylococcus | 0.413411 | 0.1797 | small | CELLULAR PROCESSES |
| Cobalamin synthesis | 0.413411 | 0.1797 | small | METABOLISM |
| Cyanobacterial bypass in the TCA | 0.449137 | 0.1668 | small | ENERGY |
| Cytochrome d ubiquinol oxidase operon | 0.449137 | 0.1668 | small | ENERGY |
| D-threonate, L-threonate and D-erythronate utilization | 0.449137 | 0.1668 | small | METABOLISM |
| Enterobacterial common antigen (LPS O-antigen) | 0.449137 | 0.1668 | small | CELL ENVELOPE |
| Extracellular matrix proteins (PEL) involved in glucose-rich biofilm formation in Pseudomonas | 0.449137 | 0.1668 | small | CELL ENVELOPE |
| Fatty acid catabolic operon fadN-fadA-fadE (yusJKL) | 0.449137 | 0.1668 | small | METABOLISM |
| FeS cluster assembly | 0.449137 | 0.1668 | small | METABOLISM |
| Adenylylsulfate reductase | 0.449137 | 0.1668 | small | ENERGY |
| Galactosamine Substituent of Arabinogalactan in Mycobacteria | 0.449137 | 0.1668 | small | CELL ENVELOPE |
| Adhesins in Staphylococcus | 0.449137 | 0.1668 | small | STRESS RESPONSE, DEFENSE, VIRULENCE |
| Aerotolerance operon | 0.449137 | 0.1668 | small | STRESS RESPONSE, DEFENSE, VIRULENCE |
| Glyoxylate bypass | 0.449137 | 0.1668 | small | ENERGY |
| Aminoglycoside modifying enzymes: O-phosphotransferases | 0.449137 | 0.1668 | small | STRESS RESPONSE, DEFENSE, VIRULENCE |
| Methylglyoxal Metabolism | 0.449137 | 0.1668 | small | ENERGY |
| Multiple Antibiotic Resistance MAR locus | 0.449137 | 0.1668 | small | STRESS RESPONSE, DEFENSE, VIRULENCE |
| Mycobacterial gene cluster associated with resistance against FAS-II antibiotics | 0.449137 | 0.1668 | small | METABOLISM |
| 2-aminophenol Metabolism | 0.449137 | 0.1668 | small | METABOLISM |
| Protein chaperones | 0.449137 | 0.1668 | small | PROTEIN PROCESSING |
| Resistance to Capreomycin and Viomycin | 0.449137 | 0.1668 | small | STRESS RESPONSE, DEFENSE, VIRULENCE |
| Resistance to chromium compounds | 0.449137 | 0.1668 | small | STRESS RESPONSE, DEFENSE, VIRULENCE |
| Bacillithiol synthesis | 0.449137 | 0.1668 | small | STRESS RESPONSE, DEFENSE, VIRULENCE |
| p-Aminobenzoyl-Glutamate Utilization | 0.449137 | 0.1668 | small | METABOLISM |
| tRNA aminoacylation, Gly | 0.449137 | 0.1668 | small | PROTEIN PROCESSING |
| tRNA aminoacylation, Val | 0.449137 | 0.1668 | small | PROTEIN PROCESSING |
| BtuS-PduT gene cluster for metalloporphyrine salvage | 0.449137 | 0.1668 | small | METABOLISM |
| Cardiolipin biosynthesis | 0.449137 | 0.1668 | small | METABOLISM |
| Coenzyme A gjo | 0.449137 | 0.1668 | small | METABOLISM |
| Fosfomycin resistance | 0.486503 | 0.154 | small | STRESS RESPONSE, DEFENSE, VIRULENCE |
| Hydroxy-fatty acid production as stress response | 0.486503 | 0.154 | small | STRESS RESPONSE, DEFENSE, VIRULENCE |
| Quorum sensing in Yersinia | 0.486503 | 0.154 | small | CELLULAR PROCESSES |
| Ribosomal hibernation related cluster | 0.486503 | 0.154 | small | CELLULAR PROCESSES |
| Signal peptidase | 0.486503 | 0.154 | small | PROTEIN PROCESSING |
| Barnasebarstar complex | 0.486503 | 0.154 | small | CELLULAR PROCESSES |
| Bile hydrolysis | 0.486503 | 0.154 | small | STRESS RESPONSE, DEFENSE, VIRULENCE |
| DeNovo Purine Biosynthesis | 0.525399 | 0.1412 | small | METABOLISM |
| Glycine betaine synthesis from choline | 0.525399 | 0.1412 | small | STRESS RESPONSE, DEFENSE, VIRULENCE |
| HipAB system implicated in growth arrest, persistence and drug tolerance | 0.525399 | 0.1412 | small | CELLULAR PROCESSES |
| IMP and Xanthine conversion to XMP and GMP module | 0.525399 | 0.1412 | small | METABOLISM |
| Aminoglycoside modifying enzymes: O-nucleotidyltransferases | 0.525399 | 0.1412 | small | STRESS RESPONSE, DEFENSE, VIRULENCE |
| Antibiotic targets in cell wall biosynthesis | 0.525399 | 0.1412 | small | STRESS RESPONSE, DEFENSE, VIRULENCE |
| Periplasmic nitrate reductase EC 1.7.99.4 | 0.525399 | 0.1412 | small | ENERGY |
| Polymyxin resistance | 0.525399 | 0.1412 | small | STRESS RESPONSE, DEFENSE, VIRULENCE |
| Resistance to Triclosan | 0.525399 | 0.1412 | small | STRESS RESPONSE, DEFENSE, VIRULENCE |
| Selenocysteine metabolism | 0.525399 | 0.1412 | small | PROTEIN PROCESSING |
| Siderophore assembly kit | 0.525399 | 0.1412 | small | METABOLISM |
| tRNA aminoacylation, Ala | 0.525399 | 0.1412 | small | PROTEIN PROCESSING |
| Bacitracin resistance | 0.525399 | 0.1412 | small | STRESS RESPONSE, DEFENSE, VIRULENCE |
| tRNA aminoacylation, Asp | 0.525399 | 0.1412 | small | PROTEIN PROCESSING |
| CoA disulfide thioldisulfide redox system | 0.525399 | 0.1412 | small | STRESS RESPONSE, DEFENSE, VIRULENCE |
| Folate Biosynthesis | 0.565784 | 0.1283 | small | METABOLISM |
| Formate dehydrogenase | 0.565784 | 0.1283 | small | ENERGY |
| L-fucose utilization | 0.565784 | 0.1283 | small | METABOLISM |
| Molybdenum cofactor biosynthesis | 0.565784 | 0.1283 | small | METABOLISM |
| Osmotic stress cluster | 0.565784 | 0.1283 | small | STRESS RESPONSE, DEFENSE, VIRULENCE |
| Antibiotic targets in transcription | 0.565784 | 0.1283 | small | STRESS RESPONSE, DEFENSE, VIRULENCE |
| Ribitol utilization | 0.565784 | 0.1283 | small | METABOLISM |
| Arginine succinyltransferase pathway | 0.565784 | 0.1283 | small | METABOLISM |
| Toxin-antitoxin replicon stabilization systems | 0.565784 | 0.1283 | small | CELLULAR PROCESSES |
| Autoinducer 2 (AI-2) transport and processing (lsrACDBFGE operon) | 0.565784 | 0.1283 | small | CELLULAR PROCESSES |
| Urease subunits | 0.565784 | 0.1283 | small | METABOLISM |
| ZraPRS-based zinc resistance | 0.565784 | 0.1283 | small | STRESS RESPONSE, DEFENSE, VIRULENCE |
| Cysteine synthesis | 0.607524 | 0.1155 | small | METABOLISM |
| De Novo Pyrimidine Synthesis | 0.607524 | 0.1155 | small | METABOLISM |
| L-ascorbate utilization (and related gene clusters) | 0.607524 | 0.1155 | small | METABOLISM |
| Lfucose dissimilation cluster | 0.607524 | 0.1155 | small | METABOLISM |
| Malonate decarboxylase | 0.607524 | 0.1155 | small | METABOLISM |
| Phosphatidylinositol mannosides biosynthesis related cluster | 0.607524 | 0.1155 | small | CELL ENVELOPE |
| Putative TldE-TldD proteolytic complex | 0.607524 | 0.1155 | small | PROTEIN PROCESSING |
| Rcs two-component regulator of capsule synthesis | 0.607524 | 0.1155 | small | CELL ENVELOPE |
| S-Adenosyl-L-homocysteine recycling | 0.607524 | 0.1155 | small | METABOLISM |
| Siderophore Aerobactin | 0.607524 | 0.1155 | small | METABOLISM |
| Smethylmethionine | 0.607524 | 0.1155 | small | METABOLISM |
| Sporulation-associated proteins with broader functions | 0.607524 | 0.1155 | small | CELLULAR PROCESSES |
| Twin-arginine translocation system | 0.607524 | 0.1155 | small | PROTEIN PROCESSING |
| tRNA aminoacylation, Trp | 0.607524 | 0.1155 | small | PROTEIN PROCESSING |
| A Hypothetical Protein Related to Proline Metabolism | 0.607524 | 0.1155 | small | METABOLISM |
| Biotin synthesis & utilization | 0.607524 | 0.1155 | small | METABOLISM |
| Citrate lyase | 0.607524 | 0.1155 | small | ENERGY |
| D-3-phosphoglycerate to phosphoserine module | 0.65055 | 0.1027 | small | METABOLISM |
| DAP (1,3-diaminopropane) production | 0.65055 | 0.1027 | small | METABOLISM |
| Dalanylation of teichoic acid | 0.65055 | 0.1027 | small | CELL ENVELOPE |
| Glutamate dehydrogenases | 0.65055 | 0.1027 | small | METABOLISM |
| Glutathione: Redox cycle | 0.65055 | 0.1027 | small | STRESS RESPONSE, DEFENSE, VIRULENCE |
| Glycolysis and Gluconeogenesis, including Archaeal enzymes | 0.65055 | 0.1027 | small | ENERGY |
| Hyaluronate utilization | 0.65055 | 0.1027 | small | METABOLISM |
| Hydrolysis of sphingomyelin | 0.65055 | 0.1027 | small | STRESS RESPONSE, DEFENSE, VIRULENCE |
| Iron siderophore sensor & receptor system | 0.65055 | 0.1027 | small | METABOLISM |
| Lgalactonate utilization | 0.65055 | 0.1027 | small | METABOLISM |
| Anaerobic glycerol-3-phosphate dehydrogenase subunits | 0.65055 | 0.1027 | small | ENERGY |
| Nicotinic acid utilization | 0.65055 | 0.1027 | small | METABOLISM |
| Putative oxidase COG2907 | 0.65055 | 0.1027 | small | METABOLISM |
| Quinolone (Fluoroquinolone) resistance via Pentapeptide repeat proteins | 0.65055 | 0.1027 | small | STRESS RESPONSE, DEFENSE, VIRULENCE |
| SecY2-SecA2 Specialized Transport System | 0.65055 | 0.1027 | small | PROTEIN PROCESSING |
| Siderophore Mycobactin | 0.65055 | 0.1027 | small | METABOLISM |
| Thiamin, thiazole, hydroxymethylpyrimidine salvage and uptake | 0.65055 | 0.1027 | small | METABOLISM |
| Translation elongation factors, bacterial | 0.65055 | 0.1027 | small | PROTEIN PROCESSING |
| 2-ketoacid oxidoreductases disambiguation | 0.65055 | 0.1027 | small | ENERGY |
| Tricarballylate Utilization | 0.65055 | 0.1027 | small | METABOLISM |
| Ubiquinone biosynthesis | 0.65055 | 0.1027 | small | METABOLISM |
| dTDP-3-acetamido-3,6-dideoxy-alpha-D-galactose synthesis | 0.65055 | 0.1027 | small | CELL ENVELOPE |
| dTDP-rhamnose synthesis | 0.65055 | 0.1027 | small | CELL ENVELOPE |
| tRNA aminoacylation, Arg | 0.65055 | 0.1027 | small | PROTEIN PROCESSING |
| tRNA aminoacylation, Cys | 0.65055 | 0.1027 | small | PROTEIN PROCESSING |
| Bacteroides capsular polysaccharide transcription antitermination proteins | 0.65055 | 0.1027 | small | CELL ENVELOPE |
| Biotin biosynthesis | 0.65055 | 0.1027 | small | METABOLISM |
| Branched-chain amino acids and alpha-keto acids utilization as energy sources | 0.65055 | 0.1027 | small | ENERGY |
| Acetoin, butanediol metabolism | 0.65055 | 0.1027 | small | ENERGY |
| Choline uptake and conversion to betaine clusters | 0.65055 | 0.1027 | small | STRESS RESPONSE, DEFENSE, VIRULENCE |
| Cob(I)alamin adenosyltransferase | 0.65055 | 0.1027 | small | METABOLISM |
| D-allose utilzation | 0.694703 | 0.0898 | small | METABOLISM |
| Degradation of branched-chain amino acids and alpha-keto acids [Leu, Val] | 0.694703 | 0.0898 | small | METABOLISM |
| Ethanolamine utilization | 0.694703 | 0.0898 | small | METABOLISM |
| Glutaconate CoA-transferase or 3-oxoadipate CoA-transferase Subunits | 0.694703 | 0.0898 | small | METABOLISM |
| Isoprenoid Biosynthesis: Interconversions | 0.694703 | 0.0898 | small | METABOLISM |
| Lfucose utilization | 0.694703 | 0.0898 | small | METABOLISM |
| Lipoylation-related cluster | 0.694703 | 0.0898 | small | METABOLISM |
| Lrhamnonate utilization cluster | 0.694703 | 0.0898 | small | METABOLISM |
| Periplasmic disulfide interchange | 0.694703 | 0.0898 | small | PROTEIN PROCESSING |
| Phosphonate metabolism | 0.694703 | 0.0898 | small | METABOLISM |
| Pyruvate metabolism I: anaplerotic reactions, PEP | 0.694703 | 0.0898 | small | ENERGY |
| Ribosomal proteins, single-copy | 0.694703 | 0.0898 | small | PROTEIN PROCESSING |
| S-methylmethionine | 0.694703 | 0.0898 | small | METABOLISM |
| Tagatose utilization | 0.694703 | 0.0898 | small | METABOLISM |
| VraTSR and LiaFSR three-component regulatory systems | 0.694703 | 0.0898 | small | STRESS RESPONSE, DEFENSE, VIRULENCE |
| Cell division related cluster | 0.694703 | 0.0898 | small | CELLULAR PROCESSES |
| D-arabinitol utilization | 0.739898 | 0.077 | small | METABOLISM |
| Encapsulation of DyP-type peroxidase or ferritin-like protein oligomers | 0.739898 | 0.077 | small | PROTEIN PROCESSING |
| Gram-negative cluster | 0.739898 | 0.077 | small | CELL ENVELOPE |
| Homoprotocatechuate degradative cluster | 0.739898 | 0.077 | small | METABOLISM |
| Lactose utilization | 0.739898 | 0.077 | small | METABOLISM |
| NiFe hydrogenase maturation | 0.739898 | 0.077 | small | ENERGY |
| Outer membrane proteases (Omptins) | 0.739898 | 0.077 | small | STRESS RESPONSE, DEFENSE, VIRULENCE |
| Pentose phosphate pathway | 0.739898 | 0.077 | small | ENERGY |
| Propanediol utilization | 0.739898 | 0.077 | small | METABOLISM |
| Pyruvate:ferredoxin oxidoreductase | 0.739898 | 0.077 | small | ENERGY |
| Ribosomal protein S12p Asp methylthiotransferase | 0.739898 | 0.077 | small | PROTEIN PROCESSING |
| tRNA aminoacylation, Thr | 0.739898 | 0.077 | small | PROTEIN PROCESSING |
| tRNA aminoacylation, Tyr | 0.739898 | 0.077 | small | PROTEIN PROCESSING |
| Biogenesis of cytochrome c oxidases | 0.739898 | 0.077 | small | ENERGY |
| A new toxin antitoxin system | 0.739898 | 0.077 | small | CELLULAR PROCESSES |
| Cyanophycin Metabolism | 0.785949 | 0.0642 | small | METABOLISM |
| Fatty Acid Biosynthesis cluster | 0.785949 | 0.0642 | small | METABOLISM |
| Fermentations: Lactate | 0.785949 | 0.0642 | small | ENERGY |
| Glutathione analogs: mycothiol | 0.785949 | 0.0642 | small | STRESS RESPONSE, DEFENSE, VIRULENCE |
| KDO2-Lipid A biosynthesis | 0.785949 | 0.0642 | small | CELL ENVELOPE |
| Lascorbate utilization (and related gene clusters) | 0.785949 | 0.0642 | small | METABOLISM |
| Lserine dehydratase subunits | 0.785949 | 0.0642 | small | METABOLISM |
| Anaerobic Oxidative Degradation of L-Ornithine | 0.785949 | 0.0642 | small | METABOLISM |
| Macrolides, lincosamides, streptogramins, ketolides, oxazolidinones (MLSKO) resistance: enzymatic degradation | 0.785949 | 0.0642 | small | STRESS RESPONSE, DEFENSE, VIRULENCE |
| Mycobacterial heme acquisition system | 0.785949 | 0.0642 | small | METABOLISM |
| Outer membrane porins in Pseudomonas and Acinetobacter | 0.785949 | 0.0642 | small | CELL ENVELOPE |
| Phd-Doc, YdcE-YdcD toxin-antitoxin (programmed cell death) systems | 0.785949 | 0.0642 | small | CELLULAR PROCESSES |
| Polyamine Metabolism | 0.785949 | 0.0642 | small | METABOLISM |
| Possible new toxin-antitoxin system including DivIC | 0.785949 | 0.0642 | small | CELLULAR PROCESSES |
| Possible stress related actinobacterial cluster | 0.785949 | 0.0642 | small | STRESS RESPONSE, DEFENSE, VIRULENCE |
| Proline biosynthesis (for review) | 0.785949 | 0.0642 | small | METABOLISM |
| Pyruvate formate-lyase cluster | 0.785949 | 0.0642 | small | ENERGY |
| Resistance to the fluoroquinolones norfloxacin and ciprofloxacin | 0.785949 | 0.0642 | small | STRESS RESPONSE, DEFENSE, VIRULENCE |
| Arsenic resistance | 0.785949 | 0.0642 | small | STRESS RESPONSE, DEFENSE, VIRULENCE |
| Thiazole.oxazolemodified microcins | 0.785949 | 0.0642 | small | METABOLISM |
| Toxin-Antitoxin system HigAB | 0.785949 | 0.0642 | small | CELLULAR PROCESSES |
| Tryptophan synthesis | 0.785949 | 0.0642 | small | METABOLISM |
| dTDP-rhamnose synthesis -- gjo | 0.785949 | 0.0642 | small | CELL ENVELOPE |
| Biotin synthesis cluster | 0.785949 | 0.0642 | small | METABOLISM |
| Cell wall-associated cluster in Mycobacterium | 0.785949 | 0.0642 | small | CELL ENVELOPE |
| Acyl carrier protein | 0.832752 | 0.0513 | small | METABOLISM |
| Glutathione: Biosynthesis and gamma-glutamyl cycle | 0.832752 | 0.0513 | small | STRESS RESPONSE, DEFENSE, VIRULENCE |
| Glutathione: Non-redox reactions | 0.832752 | 0.0513 | small | STRESS RESPONSE, DEFENSE, VIRULENCE |
| Lipopolysaccharide in K12 | 0.832752 | 0.0513 | small | CELL ENVELOPE |
| Metal chelatases | 0.832752 | 0.0513 | small | METABOLISM |
| Rcn nickel and cobalt homeostasis system | 0.832752 | 0.0513 | small | STRESS RESPONSE, DEFENSE, VIRULENCE |
| Stress proteins YciF, YciE | 0.832752 | 0.0513 | small | STRESS RESPONSE, DEFENSE, VIRULENCE |
| Teicoplanin resistance in Staphylococci | 0.832752 | 0.0513 | small | STRESS RESPONSE, DEFENSE, VIRULENCE |
| Translation termination factors, bacterial | 0.832752 | 0.0513 | small | PROTEIN PROCESSING |
| Twinarginine translocation system | 0.832752 | 0.0513 | small | PROTEIN PROCESSING |
| VType ATP synthase | 0.832752 | 0.0513 | small | ENERGY |
| Cobalamin synthesis from Cob(II)yrinate a,c diamide | 0.832752 | 0.0513 | small | METABOLISM |
| Copper homeostasis: copper tolerance | 0.832752 | 0.0513 | small | STRESS RESPONSE, DEFENSE, VIRULENCE |
| Fusaric acid resistance cluster | 0.880112 | 0.0385 | small | STRESS RESPONSE, DEFENSE, VIRULENCE |
| GDP-fucose to mannose-1P module | 0.880112 | 0.0385 | small | METABOLISM |
| Inositol catabolism | 0.880112 | 0.0385 | small | METABOLISM |
| Macrolides, lincosamides, streptogramins, ketolides, oxazolidinones (MLSKO) resistance: rRNA methylases | 0.880112 | 0.0385 | small | STRESS RESPONSE, DEFENSE, VIRULENCE |
| N-acetylneuraminate utilization | 0.880112 | 0.0385 | small | METABOLISM |
| Nacetylneuraminate utilization | 0.880112 | 0.0385 | small | METABOLISM |
| Natranslocating NADHquinone oxidoreductase | 0.880112 | 0.0385 | small | ENERGY |
| Oxaloacetate decarboxylase Na-pump | 0.880112 | 0.0385 | small | ENERGY |
| Pyruvate Alanine Serine Interconversions | 0.880112 | 0.0385 | small | ENERGY |
| Respiratory Complex I | 0.880112 | 0.0385 | small | ENERGY |
| Arginine decarboxylase and Agmatinase cluster | 0.880112 | 0.0385 | small | METABOLISM |
| Ribosome activity modulation | 0.880112 | 0.0385 | small | PROTEIN PROCESSING |
| Siderophore Yersiniabactin Biosynthesis | 0.880112 | 0.0385 | small | METABOLISM |
| Tetrathionate respiration | 0.880112 | 0.0385 | small | ENERGY |
| Tol-Pal Cell Envelope Complex | 0.880112 | 0.0385 | small | CELL ENVELOPE |
| Translation initiation factors, bacterial | 0.880112 | 0.0385 | small | PROTEIN PROCESSING |
| Biogenesis of ctype cytochromes | 0.880112 | 0.0385 | small | ENERGY |
| Chloramphenicol resistance | 0.880112 | 0.0385 | small | STRESS RESPONSE, DEFENSE, VIRULENCE |
| Entericidin | 0.927908 | 0.0257 | small | CELLULAR PROCESSES |
| Entner-Doudoroff Pathway | 0.927908 | 0.0257 | small | ENERGY |
| F0F1-type ATP synthase | 0.927908 | 0.0257 | small | ENERGY |
| Murein hydrolase regulation and cell death | 0.927908 | 0.0257 | small | CELLULAR PROCESSES |
| Na-translocating NADH-quinone oxidoreductase | 0.927908 | 0.0257 | small | ENERGY |
| Oligopeptide degradation cluster | 0.927908 | 0.0257 | small | PROTEIN PROCESSING |
| Osmoregulation | 0.927908 | 0.0257 | small | STRESS RESPONSE, DEFENSE, VIRULENCE |
| Protein-O-mannosyltransferase and 16S rRNA (cytidine(1402)-2'-O)-methyltransferase cluster | 0.927908 | 0.0257 | small | CELL ENVELOPE |
| Acetyl-CoA Pathway Wood-Ljungdahl | 0.975933 | 0.0128 | small | METABOLISM |
| D-glucosaminate utilization | 0.975933 | 0.0128 | small | METABOLISM |
| Dallose utilzation | 0.975933 | 0.0128 | small | METABOLISM |
| Delta(6)-desaturase containing cluster | 0.975933 | 0.0128 | small | METABOLISM |
| Dthreonate, Lthreonate and Derythronate utilization | 0.975933 | 0.0128 | small | METABOLISM |
| Energy-conserving hydrogenase (ferredoxin) | 0.975933 | 0.0128 | small | ENERGY |
| Fructoselysine and glucoselysine | 0.975933 | 0.0128 | small | METABOLISM |
| Glycine reductase, sarcosine reductase and betaine reductase | 0.975933 | 0.0128 | small | PROTEIN PROCESSING |
| Glycolysis and Gluconeogenesis | 0.975933 | 0.0128 | small | ENERGY |
| 2-O-alpha-mannosyl-D-glycerate utilization | 0.975933 | 0.0128 | small | METABOLISM |
| Aminoglycoside modifying enzymes: N-acetyltransferases | 0.975933 | 0.0128 | small | STRESS RESPONSE, DEFENSE, VIRULENCE |
| Listeria surface proteins: Internalinlike proteins | 0.975933 | 0.0128 | small | STRESS RESPONSE, DEFENSE, VIRULENCE |
| Methionine Salvage | 0.975933 | 0.0128 | small | METABOLISM |
| Methylenetetrahydrofolate reductase | 0.975933 | 0.0128 | small | METABOLISM |
| NAD and NADP cofactor biosynthesis global | 0.975933 | 0.0128 | small | METABOLISM |
| Nucleoside uptake and degradation cluster | 0.975933 | 0.0128 | small | METABOLISM |
| Phosphonate (phosphite) dehydrogenase | 0.975933 | 0.0128 | small | METABOLISM |
| Photorespiration (oxidative C2 cycle) | 0.975933 | 0.0128 | small | METABOLISM |
| Programmed cell death toxin-antitoxin PezAT | 0.975933 | 0.0128 | small | CELLULAR PROCESSES |
| Antibiotic targets in protein synthesis | 0.975933 | 0.0128 | small | STRESS RESPONSE, DEFENSE, VIRULENCE |
| Pyridoxin (Vitamin B6) Biosynthesis | 0.975933 | 0.0128 | small | METABOLISM |
| Pyruvate formate-lyase cluster with possible role in choline utilization | 0.975933 | 0.0128 | small | ENERGY |
| Resistance to Vancomycin and Teicoplanin | 0.975933 | 0.0128 | small | STRESS RESPONSE, DEFENSE, VIRULENCE |
| Ribosomal protein S5p acylation | 0.975933 | 0.0128 | small | PROTEIN PROCESSING |
| Aromatic amino acid interconversions with aryl acids | 0.975933 | 0.0128 | small | METABOLISM |
| Spore germinant receptors | 0.975933 | 0.0128 | small | CELLULAR PROCESSES |
| Spore germination | 0.975933 | 0.0128 | small | CELLULAR PROCESSES |
| Sporulation gene orphans | 0.975933 | 0.0128 | small | CELLULAR PROCESSES |
| Sporulation proteins SigEG cluster | 0.975933 | 0.0128 | small | CELLULAR PROCESSES |
| Sporulation proteins SpoIIIAA-SpoIIIAH | 0.975933 | 0.0128 | small | CELLULAR PROCESSES |
| Sporulation proteins SpoVA cluster | 0.975933 | 0.0128 | small | CELLULAR PROCESSES |
| Aspartate to Homoserine module | 0.975933 | 0.0128 | small | METABOLISM |
| Threonine synthase cluster | 0.975933 | 0.0128 | small | PROTEIN PROCESSING |
| Translation elongation factor G family | 0.975933 | 0.0128 | small | PROTEIN PROCESSING |
| tRNA aminoacylation, Ile | 0.975933 | 0.0128 | small | PROTEIN PROCESSING |
| tRNA aminoacylation, Lys | 0.975933 | 0.0128 | small | PROTEIN PROCESSING |
| Barnase-barstar complex | 0.975933 | 0.0128 | small | CELLULAR PROCESSES |
| Carbon monoxide induced hydrogenase | 0.975933 | 0.0128 | small | ENERGY |
| Coat proteins CotJABC | 0.975933 | 0.0128 | small | CELLULAR PROCESSES |
| Coenzyme A Biosynthesis cluster | 0.975933 | 0.0128 | small | METABOLISM |
| 2,3-diacetamido-2,3-dideoxy-d-mannuronic acid | 1 | 0 | small | METABOLISM |
| Diaminopimelate Synthesis | 1 | 0 | small | METABOLISM |
| Enoyl-[ACP] reductases disambiguation | 1 | 0 | small | METABOLISM |
| Fatty acid metabolism cluster | 1 | 0 | small | METABOLISM |
| Gentisate pathway | 1 | 0 | small | METABOLISM |
| Glycine cleavage system | 1 | 0 | small | METABOLISM |
| Heme and heme d1 biosynthesis from siroheme | 1 | 0 | small | METABOLISM |
| Hexose phosphate transport system | 1 | 0 | small | METABOLISM |
| Histidine Degradation | 1 | 0 | small | METABOLISM |
| L-serine dehydratase subunits | 1 | 0 | small | METABOLISM |
| Lysine fermentation | 1 | 0 | small | METABOLISM |
| Lysine fermentation to crotonoyl-CoA | 1 | 0 | small | METABOLISM |
| Lysine leader peptide | 1 | 0 | small | METABOLISM |
| Na-driven 2-hydroxyglutarate pathway | 1 | 0 | small | ENERGY |
| Nonmevalonate Branch of Isoprenoid Biosynthesis | 1 | 0 | small | METABOLISM |
| Ribosomal proteins, zinc requirement | 1 | 0 | small | PROTEIN PROCESSING |
| Succinate dehydrogenase and Fumarate reductase cpmlexes | 1 | 0 | small | ENERGY |
| Tetracycline resistance, all mechanisms | 1 | 0 | small | STRESS RESPONSE, DEFENSE, VIRULENCE |
| Undecaprenyl-diphosphatases | 1 | 0 | small | CELL ENVELOPE |
| tRNA aminoacylation, Glu | 1 | 0 | small | PROTEIN PROCESSING |
| tRNA aminoacylation, His | 1 | 0 | small | PROTEIN PROCESSING |
| tRNA aminoacylation, Met | 1 | 0 | small | PROTEIN PROCESSING |
| Biofilm Adhesin Biosynthesis | 1 | 0 | small | CELLULAR PROCESSES |
| Cluster containing Glutathione synthetase | 1 | 0 | small | STRESS RESPONSE, DEFENSE, VIRULENCE |
| Acetolactate synthase subunits | 1 | 0 | small | ENERGY |

**ST 9. Differential Abundance of the functional profiling of stool samples based on antibiotic treatment using the Wilcoxon Signed Rank test. (n=60 samples).**

| **Function** | **p-value** | **Effect Size** | **Effect Magnitude** | **SEED Category** |
| --- | --- | --- | --- | --- |
| Citrate lyase | 0.0002 | 0.1155 | small | ENERGY |
| Lipoic acid metabolism | 0.00179 | 0.3978 | moderate | METABOLISM |
| Biogenesis of c-type cytochromes | 0.00213 | 0.231 | small | ENERGY |
| Gallic acid utilization | 0.00653 | 0.2182 | small | METABOLISM |
| Coenzyme A gjo | 0.00653 | 0.1668 | small | METABOLISM |
| Polyamine Metabolism | 0.00756 | 0.0642 | small | METABOLISM |
| Purine catabolism in Bacillus subtilis | 0.01156 | 0.2952 | small | METABOLISM |
| Resistance to Triclosan | 0.01156 | 0.1412 | small | STRESS RESPONSE, DEFENSE, VIRULENCE |
| Glycogen metabolism | 0.01513 | 0.3208 | moderate | METABOLISM |
| Enterobacterial common antigen (LPS O-antigen) | 0.01513 | 0.1668 | small | CELL ENVELOPE |
| Ethanolamine utilization | 0.01724 | 0.0898 | small | METABOLISM |
| Colicin V and Bacteriocin Production Cluster | 0.0196 | 0.2952 | small | METABOLISM |
| Proline biosynthesis (for review) | 0.0196 | 0.0642 | small | METABOLISM |
| Choline uptake and conversion to betaine clusters | 0.02221 | 0.1027 | small | STRESS RESPONSE, DEFENSE, VIRULENCE |
| De Novo Pyrimidine Synthesis | 0.02512 | 0.1155 | small | METABOLISM |
| Histidine Biosynthesis | 0.03188 | 0.5005 | large | METABOLISM |
| Dihydroxyacetone kinases | 0.03188 | 0.308 | moderate | ENERGY |
| Siderophore Anthrachelin | 0.03188 | 0.2438 | small | METABOLISM |
| Biosynthesis of Arabinogalactan in Mycobacteria | 0.03578 | 0.2952 | small | CELL ENVELOPE |
| Urea carboxylase and Allophanate hydrolase cluster | 0.04008 | 0.2952 | small | METABOLISM |
| Salmochelin-mediated Iron Acquisition | 0.04008 | 0.231 | small | METABOLISM |
| Heme O and Heme A biosynthesis | 0.04008 | 0.2182 | small | ENERGY |
| Nonmevalonate Branch of Isoprenoid Biosynthesis | 0.04008 | 0 | small | METABOLISM |
| IMP and Xanthine conversion to XMP and GMP module | 0.04478 | 0.1412 | small | METABOLISM |
| Lipoylated proteins | 0.04992 | 0.2182 | small | METABOLISM |
| Cyanobacterial bypass in the TCA | 0.04992 | 0.1668 | small | ENERGY |
| D-threonate, L-threonate and D-erythronate utilization | 0.04992 | 0.1668 | small | METABOLISM |
| Protein chaperones | 0.04992 | 0.1668 | small | PROTEIN PROCESSING |
| Toxin-antitoxin replicon stabilization systems | 0.04992 | 0.1283 | small | CELLULAR PROCESSES |
| Urease subunits | 0.04992 | 0.1283 | small | METABOLISM |
| tRNA aminoacylation, His | 0.04992 | 0 | small | PROTEIN PROCESSING |
| Siderophore Aerobactin | 0.06164 | 0.1155 | small | METABOLISM |
| tRNA aminoacylation, Ile | 0.06164 | 0.0128 | small | PROTEIN PROCESSING |
| S-Adenosyl-L-homocysteine recycling | 0.06827 | 0.1155 | small | METABOLISM |
| Twin-arginine translocation system | 0.06827 | 0.1155 | small | PROTEIN PROCESSING |
| Lfucose utilization | 0.06827 | 0.0898 | small | METABOLISM |
| Pyruvate Alanine Serine Interconversions | 0.06827 | 0.0385 | small | ENERGY |
| D-Galacturonate and D-Glucuronate Utilization | 0.07546 | 0.462 | moderate | METABOLISM |
| Phospholipid and Fatty acid biosynthesis related cluster | 0.07546 | 0.2695 | small | METABOLISM |
| Encapsulation of DyP-type peroxidase or ferritin-like protein oligomers | 0.07546 | 0.077 | small | PROTEIN PROCESSING |
| Tryptophan synthesis | 0.07546 | 0.0642 | small | METABOLISM |
| Twinarginine translocation system | 0.07546 | 0.0513 | small | PROTEIN PROCESSING |
| Entericidin | 0.07546 | 0.0257 | small | CELLULAR PROCESSES |
| Peptidase clustering with DAP | 0.08323 | 0.4363 | moderate | METABOLISM |
| Proteasome bacterial | 0.08323 | 0.2567 | small | PROTEIN PROCESSING |
| Glyoxylate bypass | 0.08323 | 0.1668 | small | ENERGY |
| ZraPRS-based zinc resistance | 0.08323 | 0.1283 | small | STRESS RESPONSE, DEFENSE, VIRULENCE |
| Degradation of branched-chain amino acids and alpha-keto acids [Leu, Val] | 0.08323 | 0.0898 | small | METABOLISM |
| Lascorbate utilization (and related gene clusters) | 0.08323 | 0.0642 | small | METABOLISM |
| Inositol catabolism | 0.08323 | 0.0385 | small | METABOLISM |
| Nacetylneuraminate utilization | 0.08323 | 0.0385 | small | METABOLISM |
| Alpha-acetolactate operon | 0.09161 | 0.1925 | small | METABOLISM |
| MazEF toxin-antitoxing (programmed cell death) system | 0.09161 | 0.1925 | small | CELLULAR PROCESSES |
| YoeB-YefM toxin-antitoxin system | 0.10063 | 0.2952 | small | CELLULAR PROCESSES |
| Aminoglycoside modifying enzymes: O-nucleotidyltransferases | 0.10063 | 0.1412 | small | STRESS RESPONSE, DEFENSE, VIRULENCE |
| Ribosome activity modulation | 0.10063 | 0.0385 | small | PROTEIN PROCESSING |
| Glutamine synthetases | 0.11032 | 0.2952 | small | METABOLISM |
| Fe-S cluster assembly | 0.11032 | 0.2695 | small | METABOLISM |
| Pyruvate metabolism II: acetyl-CoA, acetogenesis from pyruvate | 0.11032 | 0.2182 | small | ENERGY |
| tRNA aminoacylation, Val | 0.11032 | 0.1668 | small | PROTEIN PROCESSING |
| Ribosomal hibernation related cluster | 0.11032 | 0.154 | small | CELLULAR PROCESSES |
| SecY2-SecA2 Specialized Transport System | 0.11032 | 0.1027 | small | PROTEIN PROCESSING |
| Biogenesis of ctype cytochromes | 0.11032 | 0.0385 | small | ENERGY |
| S-methylmethionine | 0.12071 | 0.0898 | small | METABOLISM |
| Glutathionylspermidine and Trypanothione | 0.13182 | 0.385 | moderate | STRESS RESPONSE, DEFENSE, VIRULENCE |
| Protection from Reactive Oxygen Species | 0.13182 | 0.2952 | small | STRESS RESPONSE, DEFENSE, VIRULENCE |
| FeS cluster assembly | 0.13182 | 0.1668 | small | METABOLISM |
| Ribosomal proteins, zinc requirement | 0.13182 | 0 | small | PROTEIN PROCESSING |
| Methylglyoxal Metabolism | 0.14368 | 0.1668 | small | ENERGY |
| Metal chelatases | 0.14368 | 0.0513 | small | METABOLISM |
| Dthreonate, Lthreonate and Derythronate utilization | 0.14368 | 0.0128 | small | METABOLISM |
| Carbon storage regulator | 0.15631 | 0.2952 | small | METABOLISM |
| Homogentisate pathway | 0.15631 | 0.2053 | small | METABOLISM |
| Anaerobic dimethyl sulfoxide reductase (EC 1.8.5.3) | 0.15631 | 0.1925 | small | ENERGY |
| Toxin-Antitoxin system HigAB | 0.15631 | 0.0642 | small | CELLULAR PROCESSES |
| TonB-ExbBD energy transducing system | 0.16974 | 0.3722 | moderate | CELL ENVELOPE |
| Macrolides, lincosamides, streptogramins, ketolides, oxazolidinones (MLSKO) resistance: ribosomal protection | 0.16974 | 0.3465 | moderate | STRESS RESPONSE, DEFENSE, VIRULENCE |
| Branched-Chain Amino Acid Biosynthesis | 0.16974 | 0.2182 | small | METABOLISM |
| Fatty acid synthesis | 0.16974 | 0.1925 | small | METABOLISM |
| tRNA aminoacylation, Gly | 0.16974 | 0.1668 | small | PROTEIN PROCESSING |
| L-fucose utilization | 0.16974 | 0.1283 | small | METABOLISM |
| Succinate dehydrogenase and Fumarate reductase cpmlexes | 0.16974 | 0 | small | ENERGY |
| Pyridoxin (Vitamin B6) Biosynthesis | 0.18398 | 0.0128 | small | METABOLISM |
| Polymyxin resistance | 0.19906 | 0.1412 | small | STRESS RESPONSE, DEFENSE, VIRULENCE |
| Outer membrane porins in Pseudomonas and Acinetobacter | 0.19906 | 0.0642 | small | CELL ENVELOPE |
| Respiratory Complex I | 0.19906 | 0.0385 | small | ENERGY |
| Nucleoside uptake and degradation cluster | 0.19906 | 0.0128 | small | METABOLISM |
| Dihydropyrimidine dehydrogenase | 0.21499 | 0.3208 | moderate | METABOLISM |
| L-fucose dissimilation cluster | 0.21499 | 0.2823 | small | METABOLISM |
| Proline, 4-hydroxyproline uptake and utilization | 0.21499 | 0.2567 | small | METABOLISM |
| TCA Cycle | 0.21499 | 0.2182 | small | ENERGY |
| Glycine betaine synthesis from choline | 0.21499 | 0.1412 | small | STRESS RESPONSE, DEFENSE, VIRULENCE |
| Selenocysteine metabolism | 0.21499 | 0.1412 | small | PROTEIN PROCESSING |
| Lgalactonate utilization | 0.21499 | 0.1027 | small | METABOLISM |
| Tricarballylate Utilization | 0.21499 | 0.1027 | small | METABOLISM |
| VType ATP synthase | 0.21499 | 0.0513 | small | ENERGY |
| Coenzyme A Biosynthesis cluster | 0.21499 | 0.0128 | small | METABOLISM |
| Glycine cleavage system | 0.21499 | 0 | small | METABOLISM |
| Proline Synthesis | 0.23179 | 0.5647 | large | METABOLISM |
| Hydroxyaromatic decarboxylase family | 0.23179 | 0.5005 | large | METABOLISM |
| Heat shock dnaK gene cluster extended | 0.23179 | 0.3722 | moderate | STRESS RESPONSE, DEFENSE, VIRULENCE |
| Polymyxin resistance, lipid A modifications with 4-amino-4-deoxy-L-arabinose | 0.23179 | 0.3593 | moderate | STRESS RESPONSE, DEFENSE, VIRULENCE |
| Darabinitol utilization | 0.23179 | 0.2053 | small | METABOLISM |
| Signal peptidase | 0.23179 | 0.154 | small | PROTEIN PROCESSING |
| Ubiquinone biosynthesis | 0.23179 | 0.1027 | small | METABOLISM |
| tRNA aminoacylation, Cys | 0.23179 | 0.1027 | small | PROTEIN PROCESSING |
| Chaperones GroEL GroES and Thermosome | 0.24947 | 0.308 | moderate | PROTEIN PROCESSING |
| Multiple Antibiotic Resistance MAR locus | 0.24947 | 0.1668 | small | STRESS RESPONSE, DEFENSE, VIRULENCE |
| Cysteine synthesis | 0.24947 | 0.1155 | small | METABOLISM |
| D-3-phosphoglycerate to phosphoserine module | 0.24947 | 0.1027 | small | METABOLISM |
| D-allose utilzation | 0.24947 | 0.0898 | small | METABOLISM |
| D-arabinitol utilization | 0.24947 | 0.077 | small | METABOLISM |
| Gram-negative cluster | 0.24947 | 0.077 | small | CELL ENVELOPE |
| Dallose utilzation | 0.24947 | 0.0128 | small | METABOLISM |
| Sporulation Cluster | 0.26804 | 0.3208 | moderate | CELLULAR PROCESSES |
| Lrhamnonate utilization cluster | 0.26804 | 0.0898 | small | METABOLISM |
| Biotin synthesis cluster | 0.26804 | 0.0642 | small | METABOLISM |
| Entner-Doudoroff Pathway | 0.26804 | 0.0257 | small | ENERGY |
| Cysteine dioxygenase containing cluster 1 | 0.2875 | 0.1925 | small | METABOLISM |
| Ribitol utilization | 0.2875 | 0.1283 | small | METABOLISM |
| Murein hydrolase regulation and cell death | 0.2875 | 0.0257 | small | CELLULAR PROCESSES |
| Pyruvate formate-lyase cluster with possible role in choline utilization | 0.2875 | 0.0128 | small | ENERGY |
| Gentisate pathway | 0.2875 | 0 | small | METABOLISM |
| Fatty Acid Biosynthesis FASII | 0.30787 | 0.3593 | moderate | METABOLISM |
| Xanthine dehydrogenase subunits | 0.30787 | 0.2695 | small | METABOLISM |
| Siderophore Enterobactin | 0.30787 | 0.1797 | small | METABOLISM |
| Molybdenum cofactor biosynthesis | 0.30787 | 0.1283 | small | METABOLISM |
| Anaerobic glycerol-3-phosphate dehydrogenase subunits | 0.30787 | 0.1027 | small | ENERGY |
| dTDP-rhamnose synthesis | 0.30787 | 0.1027 | small | CELL ENVELOPE |
| Copper homeostasis: copper tolerance | 0.30787 | 0.0513 | small | STRESS RESPONSE, DEFENSE, VIRULENCE |
| GDP-fucose to mannose-1P module | 0.30787 | 0.0385 | small | METABOLISM |
| Wall polysaccharide pyruvylation | 0.32914 | 0.231 | small | CELL ENVELOPE |
| Arginine succinyltransferase pathway | 0.32914 | 0.1283 | small | METABOLISM |
| Glutathione: Biosynthesis and gamma-glutamyl cycle | 0.32914 | 0.0513 | small | STRESS RESPONSE, DEFENSE, VIRULENCE |
| Rcn nickel and cobalt homeostasis system | 0.32914 | 0.0513 | small | STRESS RESPONSE, DEFENSE, VIRULENCE |
| Phosphonate (phosphite) dehydrogenase | 0.32914 | 0.0128 | small | METABOLISM |
| Resistance to Daptomycin | 0.3513 | 0.2823 | small | STRESS RESPONSE, DEFENSE, VIRULENCE |
| D-gluconate and ketogluconates metabolism | 0.3513 | 0.2695 | small | METABOLISM |
| tRNA aminoacylation, Phe | 0.3513 | 0.1797 | small | PROTEIN PROCESSING |
| Adhesins in Staphylococcus | 0.3513 | 0.1668 | small | STRESS RESPONSE, DEFENSE, VIRULENCE |
| Hydroxy-fatty acid production as stress response | 0.3513 | 0.154 | small | STRESS RESPONSE, DEFENSE, VIRULENCE |
| Barnasebarstar complex | 0.3513 | 0.154 | small | CELLULAR PROCESSES |
| Dalanylation of teichoic acid | 0.3513 | 0.1027 | small | CELL ENVELOPE |
| Glutathione: Redox cycle | 0.3513 | 0.1027 | small | STRESS RESPONSE, DEFENSE, VIRULENCE |
| Putative oxidase COG2907 | 0.3513 | 0.1027 | small | METABOLISM |
| Phd-Doc, YdcE-YdcD toxin-antitoxin (programmed cell death) systems | 0.3513 | 0.0642 | small | CELLULAR PROCESSES |
| Tetrathionate respiration | 0.3513 | 0.0385 | small | ENERGY |
| D-glucosaminate utilization | 0.3513 | 0.0128 | small | METABOLISM |
| Spore germinant receptors | 0.3513 | 0.0128 | small | CELLULAR PROCESSES |
| tRNA aminoacylation, Glu | 0.3513 | 0 | small | PROTEIN PROCESSING |
| Ribosome LSU, bacterial | 0.37436 | 0.2952 | small | PROTEIN PROCESSING |
| Beta-lactamases Ambler class A | 0.37436 | 0.2567 | small | STRESS RESPONSE, DEFENSE, VIRULENCE |
| Uridine to beta-Alanine and Thymine to 3-aminoisobutanoate module | 0.37436 | 0.1797 | small | METABOLISM |
| Pyruvate formate-lyase cluster | 0.37436 | 0.0642 | small | ENERGY |
| tRNA aminoacylation, Asn | 0.3983 | 0.2952 | small | PROTEIN PROCESSING |
| tRNA aminoacylation, Gln | 0.3983 | 0.2438 | small | PROTEIN PROCESSING |
| CoA disulfide thiol-disulfide redox system | 0.3983 | 0.2438 | small | STRESS RESPONSE, DEFENSE, VIRULENCE |
| Phenylacetyl-CoA catabolic pathway (core) | 0.3983 | 0.231 | small | METABOLISM |
| Cold shock proteins of CSP family | 0.3983 | 0.2053 | small | STRESS RESPONSE, DEFENSE, VIRULENCE |
| 2-aminophenol Metabolism | 0.3983 | 0.1668 | small | METABOLISM |
| CoA disulfide thioldisulfide redox system | 0.3983 | 0.1412 | small | STRESS RESPONSE, DEFENSE, VIRULENCE |
| Lfucose dissimilation cluster | 0.3983 | 0.1155 | small | METABOLISM |
| Sporulation-associated proteins with broader functions | 0.3983 | 0.1155 | small | CELLULAR PROCESSES |
| Hyaluronate utilization | 0.3983 | 0.1027 | small | METABOLISM |
| Cyanophycin Metabolism | 0.3983 | 0.0642 | small | METABOLISM |
| Macrolides, lincosamides, streptogramins, ketolides, oxazolidinones (MLSKO) resistance: rRNA methylases | 0.3983 | 0.0385 | small | STRESS RESPONSE, DEFENSE, VIRULENCE |
| Oligopeptide degradation cluster | 0.3983 | 0.0257 | small | PROTEIN PROCESSING |
| Glycolysis and Gluconeogenesis | 0.3983 | 0.0128 | small | ENERGY |
| Anaerobic module of TCA | 0.42311 | 0.6032 | large | ENERGY |
| Bicyclomycin resistance cluster | 0.42311 | 0.2053 | small | STRESS RESPONSE, DEFENSE, VIRULENCE |
| tRNA aminoacylation, Asp | 0.42311 | 0.1412 | small | PROTEIN PROCESSING |
| Autoinducer 2 (AI-2) transport and processing (lsrACDBFGE operon) | 0.42311 | 0.1283 | small | CELLULAR PROCESSES |
| Putative TldE-TldD proteolytic complex | 0.42311 | 0.1155 | small | PROTEIN PROCESSING |
| Colanic acid synthesis | 0.44877 | 0.2438 | small | CELL ENVELOPE |
| Listeria surface proteins: Internalin-like proteins | 0.44877 | 0.1925 | small | STRESS RESPONSE, DEFENSE, VIRULENCE |
| Phage shock protein operon | 0.44877 | 0.1925 | small | STRESS RESPONSE, DEFENSE, VIRULENCE |
| Periplasmic nitrate reductase EC 1.7.99.4 | 0.44877 | 0.1412 | small | ENERGY |
| Folate Biosynthesis | 0.44877 | 0.1283 | small | METABOLISM |
| Glutamate dehydrogenases | 0.44877 | 0.1027 | small | METABOLISM |
| Outer membrane proteases (Omptins) | 0.44877 | 0.077 | small | STRESS RESPONSE, DEFENSE, VIRULENCE |
| L-rhamnonate utilization cluster | 0.47526 | 0.5005 | large | METABOLISM |
| Selenoprotein O | 0.47526 | 0.231 | small | PROTEIN PROCESSING |
| Glycerol fermentation to 1,3-propanediol | 0.47526 | 0.2182 | small | ENERGY |
| Acyl-CoA thioesterase II | 0.47526 | 0.1925 | small | METABOLISM |
| Glycolysis and Gluconeogenesis, including Archaeal enzymes | 0.47526 | 0.1027 | small | ENERGY |
| Homoprotocatechuate degradative cluster | 0.47526 | 0.077 | small | METABOLISM |
| Pentose phosphate pathway | 0.47526 | 0.077 | small | ENERGY |
| Siderophore Yersiniabactin Biosynthesis | 0.47526 | 0.0385 | small | METABOLISM |
| Undecaprenyl-diphosphatases | 0.47526 | 0 | small | CELL ENVELOPE |
| Cluster containing Glutathione synthetase | 0.47526 | 0 | small | STRESS RESPONSE, DEFENSE, VIRULENCE |
| Fermentations: Mixed acid | 0.50256 | 0.385 | moderate | ENERGY |
| Resistance to Ethionamide and Isoniazid | 0.50256 | 0.3593 | moderate | STRESS RESPONSE, DEFENSE, VIRULENCE |
| EnvZ and OmpR regulon | 0.50256 | 0.2438 | small | STRESS RESPONSE, DEFENSE, VIRULENCE |
| CtsR and MscAB regulation of protein degradation | 0.50256 | 0.1925 | small | PROTEIN PROCESSING |
| Smethylmethionine | 0.50256 | 0.1155 | small | METABOLISM |
| Natranslocating NADHquinone oxidoreductase | 0.50256 | 0.0385 | small | ENERGY |
| Antibiotic targets in protein synthesis | 0.50256 | 0.0128 | small | STRESS RESPONSE, DEFENSE, VIRULENCE |
| Threonine synthase cluster | 0.50256 | 0.0128 | small | PROTEIN PROCESSING |
| Lysine fermentation | 0.50256 | 0 | small | METABOLISM |
| Glutamate fermentation | 0.53065 | 0.231 | small | METABOLISM |
| Lysine DAP biosynthetic pathway | 0.53065 | 0.1925 | small | METABOLISM |
| Glycerolipid and Glycerophospholipid Metabolism in Bacteria | 0.53065 | 0.1797 | small | METABOLISM |
| Fosfomycin resistance | 0.53065 | 0.154 | small | STRESS RESPONSE, DEFENSE, VIRULENCE |
| Antibiotic targets in transcription | 0.53065 | 0.1283 | small | STRESS RESPONSE, DEFENSE, VIRULENCE |
| A Hypothetical Protein Related to Proline Metabolism | 0.53065 | 0.1155 | small | METABOLISM |
| Macrolides, lincosamides, streptogramins, ketolides, oxazolidinones (MLSKO) resistance: enzymatic degradation | 0.53065 | 0.0642 | small | STRESS RESPONSE, DEFENSE, VIRULENCE |
| Oxaloacetate decarboxylase Na-pump | 0.53065 | 0.0385 | small | ENERGY |
| Na-translocating NADH-quinone oxidoreductase | 0.53065 | 0.0257 | small | ENERGY |
| Sporulation proteins SpoVA cluster | 0.53065 | 0.0128 | small | CELLULAR PROCESSES |
| Hexose phosphate transport system | 0.53065 | 0 | small | METABOLISM |
| tRNA aminoacylation, Pro | 0.55948 | 0.3208 | moderate | PROTEIN PROCESSING |
| Beta-lactamases Ambler class C | 0.55948 | 0.2438 | small | STRESS RESPONSE, DEFENSE, VIRULENCE |
| Tetracycline resistance, ribosomal protection type | 0.55948 | 0.231 | small | STRESS RESPONSE, DEFENSE, VIRULENCE |
| Bile hydrolysis | 0.55948 | 0.154 | small | STRESS RESPONSE, DEFENSE, VIRULENCE |
| DAP (1,3-diaminopropane) production | 0.55948 | 0.1027 | small | METABOLISM |
| Lipoylation-related cluster | 0.55948 | 0.0898 | small | METABOLISM |
| Glycine reductase, sarcosine reductase and betaine reductase | 0.55948 | 0.0128 | small | PROTEIN PROCESSING |
| Antibiotic targets in metabolic pathways | 0.58903 | 0.1925 | small | STRESS RESPONSE, DEFENSE, VIRULENCE |
| Biofilm formation in Staphylococcus | 0.58903 | 0.1797 | small | CELLULAR PROCESSES |
| BtuS-PduT gene cluster for metalloporphyrine salvage | 0.58903 | 0.1668 | small | METABOLISM |
| Quorum sensing in Yersinia | 0.58903 | 0.154 | small | CELLULAR PROCESSES |
| Bacteroides capsular polysaccharide transcription antitermination proteins | 0.58903 | 0.1027 | small | CELL ENVELOPE |
| Tagatose utilization | 0.58903 | 0.0898 | small | METABOLISM |
| VraTSR and LiaFSR three-component regulatory systems | 0.58903 | 0.0898 | small | STRESS RESPONSE, DEFENSE, VIRULENCE |
| Translation termination factors, bacterial | 0.58903 | 0.0513 | small | PROTEIN PROCESSING |
| Osmoregulation | 0.58903 | 0.0257 | small | STRESS RESPONSE, DEFENSE, VIRULENCE |
| Acetyl-CoA Pathway Wood-Ljungdahl | 0.58903 | 0.0128 | small | METABOLISM |
| Aminoglycoside modifying enzymes: N-acetyltransferases | 0.58903 | 0.0128 | small | STRESS RESPONSE, DEFENSE, VIRULENCE |
| Methylenetetrahydrofolate reductase | 0.58903 | 0.0128 | small | METABOLISM |
| Sporulation proteins SpoIIIAA-SpoIIIAH | 0.58903 | 0.0128 | small | CELLULAR PROCESSES |
| Carbon monoxide induced hydrogenase | 0.58903 | 0.0128 | small | ENERGY |
| Galactose utilization | 0.61926 | 0.3337 | moderate | METABOLISM |
| Dehydrogenase complexes | 0.61926 | 0.2053 | small | ENERGY |
| GMP synthase | 0.61926 | 0.2053 | small | METABOLISM |
| Tolerance to colicin E2 and CreBC signal transduction system | 0.61926 | 0.1925 | small | METABOLISM |
| Urea cycle | 0.61926 | 0.1797 | small | METABOLISM |
| Resistance to chromium compounds | 0.61926 | 0.1668 | small | STRESS RESPONSE, DEFENSE, VIRULENCE |
| Bacitracin resistance | 0.61926 | 0.1412 | small | STRESS RESPONSE, DEFENSE, VIRULENCE |
| Thiamin, thiazole, hydroxymethylpyrimidine salvage and uptake | 0.61926 | 0.1027 | small | METABOLISM |
| Acetoin, butanediol metabolism | 0.61926 | 0.1027 | small | ENERGY |
| Cell division related cluster | 0.61926 | 0.0898 | small | CELLULAR PROCESSES |
| Acyl carrier protein | 0.61926 | 0.0513 | small | METABOLISM |
| Histidine Degradation | 0.61926 | 0 | small | METABOLISM |
| Poly-gamma-glutamate biosynthesis | 0.65012 | 0.3593 | moderate | METABOLISM |
| tRNA aminoacylation, Leu | 0.65012 | 0.308 | moderate | PROTEIN PROCESSING |
| NADH ubiquinone oxidoreductase vs. multi-subunit cation antiporter | 0.65012 | 0.2823 | small | ENERGY |
| Purine nucleotide synthesis regulator | 0.65012 | 0.231 | small | METABOLISM |
| L-galactonate utilization | 0.65012 | 0.2182 | small | METABOLISM |
| Cobalamin synthesis, Precorin-2 to Cob(II)yrinate a,c diamide, aerobic | 0.65012 | 0.2182 | small | METABOLISM |
| Pyrroloquinoline Quinone biosynthesis | 0.65012 | 0.2053 | small | METABOLISM |
| V-Type ATP synthase | 0.65012 | 0.2053 | small | ENERGY |
| Actinobacterial signal transduction system MtrAB-LpqB | 0.65012 | 0.1797 | small | STRESS RESPONSE, DEFENSE, VIRULENCE |
| Extracellular matrix proteins (PEL) involved in glucose-rich biofilm formation in Pseudomonas | 0.65012 | 0.1668 | small | CELL ENVELOPE |
| Fatty acid catabolic operon fadN-fadA-fadE (yusJKL) | 0.65012 | 0.1668 | small | METABOLISM |
| Adenylylsulfate reductase | 0.65012 | 0.1668 | small | ENERGY |
| Galactosamine Substituent of Arabinogalactan in Mycobacteria | 0.65012 | 0.1668 | small | CELL ENVELOPE |
| Mycobacterial gene cluster associated with resistance against FAS-II antibiotics | 0.65012 | 0.1668 | small | METABOLISM |
| Bacillithiol synthesis | 0.65012 | 0.1668 | small | STRESS RESPONSE, DEFENSE, VIRULENCE |
| Cardiolipin biosynthesis | 0.65012 | 0.1668 | small | METABOLISM |
| Antibiotic targets in cell wall biosynthesis | 0.65012 | 0.1412 | small | STRESS RESPONSE, DEFENSE, VIRULENCE |
| Siderophore assembly kit | 0.65012 | 0.1412 | small | METABOLISM |
| Hydrolysis of sphingomyelin | 0.65012 | 0.1027 | small | STRESS RESPONSE, DEFENSE, VIRULENCE |
| Iron siderophore sensor & receptor system | 0.65012 | 0.1027 | small | METABOLISM |
| Quinolone (Fluoroquinolone) resistance via Pentapeptide repeat proteins | 0.65012 | 0.1027 | small | STRESS RESPONSE, DEFENSE, VIRULENCE |
| Siderophore Mycobactin | 0.65012 | 0.1027 | small | METABOLISM |
| dTDP-3-acetamido-3,6-dideoxy-alpha-D-galactose synthesis | 0.65012 | 0.1027 | small | CELL ENVELOPE |
| Phosphonate metabolism | 0.65012 | 0.0898 | small | METABOLISM |
| Biogenesis of cytochrome c oxidases | 0.65012 | 0.077 | small | ENERGY |
| Lserine dehydratase subunits | 0.65012 | 0.0642 | small | METABOLISM |
| Mycobacterial heme acquisition system | 0.65012 | 0.0642 | small | METABOLISM |
| Possible stress related actinobacterial cluster | 0.65012 | 0.0642 | small | STRESS RESPONSE, DEFENSE, VIRULENCE |
| Resistance to the fluoroquinolones norfloxacin and ciprofloxacin | 0.65012 | 0.0642 | small | STRESS RESPONSE, DEFENSE, VIRULENCE |
| Thiazole.oxazolemodified microcins | 0.65012 | 0.0642 | small | METABOLISM |
| Cell wall-associated cluster in Mycobacterium | 0.65012 | 0.0642 | small | CELL ENVELOPE |
| Chloramphenicol resistance | 0.65012 | 0.0385 | small | STRESS RESPONSE, DEFENSE, VIRULENCE |
| Protein-O-mannosyltransferase and 16S rRNA (cytidine(1402)-2'-O)-methyltransferase cluster | 0.65012 | 0.0257 | small | CELL ENVELOPE |
| Listeria surface proteins: Internalinlike proteins | 0.65012 | 0.0128 | small | STRESS RESPONSE, DEFENSE, VIRULENCE |
| Aspartate to Homoserine module | 0.65012 | 0.0128 | small | METABOLISM |
| Barnase-barstar complex | 0.65012 | 0.0128 | small | CELLULAR PROCESSES |
| Heme and heme d1 biosynthesis from siroheme | 0.65012 | 0 | small | METABOLISM |
| Lysine fermentation to crotonoyl-CoA | 0.65012 | 0 | small | METABOLISM |
| Na-driven 2-hydroxyglutarate pathway | 0.65012 | 0 | small | ENERGY |
| Chitobiose | 0.68157 | 0.4363 | moderate | METABOLISM |
| Mevalonate metabolic pathway | 0.68157 | 0.2952 | small | METABOLISM |
| Arginine biosynthesis | 0.68157 | 0.2952 | small | METABOLISM |
| Aspartate to Threonine Module | 0.68157 | 0.1925 | small | METABOLISM |
| Bacterial Sphingolipids | 0.68157 | 0.1925 | small | METABOLISM |
| tRNA aminoacylation, Ala | 0.68157 | 0.1412 | small | PROTEIN PROCESSING |
| Osmotic stress cluster | 0.68157 | 0.1283 | small | STRESS RESPONSE, DEFENSE, VIRULENCE |
| Propanediol utilization | 0.68157 | 0.077 | small | METABOLISM |
| dTDP-rhamnose synthesis -- gjo | 0.68157 | 0.0642 | small | CELL ENVELOPE |
| Programmed cell death toxin-antitoxin PezAT | 0.68157 | 0.0128 | small | CELLULAR PROCESSES |
| Sugar-phosphate stress regulation | 0.71357 | 0.4235 | moderate | STRESS RESPONSE, DEFENSE, VIRULENCE |
| Trimethylamine-N-oxide operon | 0.71357 | 0.3337 | moderate | ENERGY |
| Aerotolerance operon | 0.71357 | 0.1668 | small | STRESS RESPONSE, DEFENSE, VIRULENCE |
| Ribosomal protein S12p Asp methylthiotransferase | 0.71357 | 0.077 | small | PROTEIN PROCESSING |
| A new toxin antitoxin system | 0.71357 | 0.077 | small | CELLULAR PROCESSES |
| Fusaric acid resistance cluster | 0.71357 | 0.0385 | small | STRESS RESPONSE, DEFENSE, VIRULENCE |
| Fructoselysine and glucoselysine | 0.71357 | 0.0128 | small | METABOLISM |
| Photorespiration (oxidative C2 cycle) | 0.71357 | 0.0128 | small | METABOLISM |
| Heme Biosynthesis: protoporphyrin-, coproporphyrin- and siroheme-dependent pathways | 0.74607 | 0.4748 | moderate | METABOLISM |
| Putrescine utilization | 0.74607 | 0.3465 | moderate | METABOLISM |
| Outer membrane | 0.74607 | 0.2695 | small | CELL ENVELOPE |
| Cobalamin synthesis, Precorin-2 to Cob(II)yrinate a,c diamide, anaerobic | 0.74607 | 0.2182 | small | METABOLISM |
| Riboflavin, FMN and FAD metabolism with fusion events | 0.74607 | 0.1797 | small | METABOLISM |
| DeNovo Purine Biosynthesis | 0.74607 | 0.1412 | small | METABOLISM |
| Possible new toxin-antitoxin system including DivIC | 0.74607 | 0.0642 | small | CELLULAR PROCESSES |
| Tol-Pal Cell Envelope Complex | 0.74607 | 0.0385 | small | CELL ENVELOPE |
| Translation elongation factor G family | 0.74607 | 0.0128 | small | PROTEIN PROCESSING |
| 2,3-diacetamido-2,3-dideoxy-d-mannuronic acid | 0.74607 | 0 | small | METABOLISM |
| L-serine dehydratase subunits | 0.74607 | 0 | small | METABOLISM |
| Nucleoside triphosphate pyrophosphohydrolase MazG | 0.77901 | 0.2823 | small | CELLULAR PROCESSES |
| Glycolate, glyoxylate interconversions | 0.77901 | 0.2182 | small | ENERGY |
| Malonate decarboxylase | 0.77901 | 0.1155 | small | METABOLISM |
| tRNA aminoacylation, Trp | 0.77901 | 0.1155 | small | PROTEIN PROCESSING |
| Translation elongation factors, bacterial | 0.77901 | 0.1027 | small | PROTEIN PROCESSING |
| Arginine decarboxylase and Agmatinase cluster | 0.77901 | 0.0385 | small | METABOLISM |
| Delta(6)-desaturase containing cluster | 0.77901 | 0.0128 | small | METABOLISM |
| Energy-conserving hydrogenase (ferredoxin) | 0.77901 | 0.0128 | small | ENERGY |
| Methionine Salvage | 0.77901 | 0.0128 | small | METABOLISM |
| Ribosomal protein S5p acylation | 0.77901 | 0.0128 | small | PROTEIN PROCESSING |
| Aromatic amino acid interconversions with aryl acids | 0.77901 | 0.0128 | small | METABOLISM |
| D-alanylation of teichoic acid | 0.81234 | 0.385 | moderate | CELL ENVELOPE |
| Peptide methionine sulfoxide reductase | 0.81234 | 0.2567 | small | PROTEIN PROCESSING |
| One-carbon metabolism by tetrahydropterines | 0.81234 | 0.231 | small | METABOLISM |
| Peptidyl-prolyl cis-trans isomerase | 0.81234 | 0.231 | small | PROTEIN PROCESSING |
| Sucrose to levan conversions | 0.81234 | 0.2182 | small | METABOLISM |
| Lipid-linked oligosaccharide synthesis related cluster | 0.81234 | 0.1797 | small | CELL ENVELOPE |
| tRNA aminoacylation, Arg | 0.81234 | 0.1027 | small | PROTEIN PROCESSING |
| Ribosomal proteins, single-copy | 0.81234 | 0.0898 | small | PROTEIN PROCESSING |
| Pyruvate:ferredoxin oxidoreductase | 0.81234 | 0.077 | small | ENERGY |
| KDO2-Lipid A biosynthesis | 0.81234 | 0.0642 | small | CELL ENVELOPE |
| Cobalamin synthesis from Cob(II)yrinate a,c diamide | 0.81234 | 0.0513 | small | METABOLISM |
| Lysine leader peptide | 0.81234 | 0 | small | METABOLISM |
| Fatty acid degradation | 0.846 | 0.3208 | moderate | METABOLISM |
| Phenylalanine and Tyrosine synthesis 1 | 0.846 | 0.3208 | moderate | METABOLISM |
| Streptococcal Hyaluronic Acid Capsule | 0.846 | 0.3208 | moderate | CELL ENVELOPE |
| L-ascorbate utilization (and related gene clusters) | 0.846 | 0.1155 | small | METABOLISM |
| 2-ketoacid oxidoreductases disambiguation | 0.846 | 0.1027 | small | ENERGY |
| N-acetylneuraminate utilization | 0.846 | 0.0385 | small | METABOLISM |
| F0F1-type ATP synthase | 0.846 | 0.0257 | small | ENERGY |
| Coat proteins CotJABC | 0.846 | 0.0128 | small | CELLULAR PROCESSES |
| Acetolactate synthase subunits | 0.846 | 0 | small | ENERGY |
| Inner membrane proteins of MarC family, not involved in antibiotic resistance | 0.87993 | 0.308 | moderate | STRESS RESPONSE, DEFENSE, VIRULENCE |
| Repair of Iron Centers | 0.87993 | 0.2182 | small | STRESS RESPONSE, DEFENSE, VIRULENCE |
| Branched-chain amino acids and alpha-keto acids utilization as energy sources | 0.87993 | 0.1027 | small | ENERGY |
| Lactose utilization | 0.87993 | 0.077 | small | METABOLISM |
| Translation initiation factors, bacterial | 0.87993 | 0.0385 | small | PROTEIN PROCESSING |
| 2-O-alpha-mannosyl-D-glycerate utilization | 0.87993 | 0.0128 | small | METABOLISM |
| tRNA aminoacylation, Lys | 0.87993 | 0.0128 | small | PROTEIN PROCESSING |
| Enoyl-[ACP] reductases disambiguation | 0.87993 | 0 | small | METABOLISM |
| tRNA aminoacylation, Ser | 0.91408 | 0.4492 | moderate | PROTEIN PROCESSING |
| Sulfur transfer pathway CsdAEL | 0.91408 | 0.2695 | small | METABOLISM |
| Cadmium resistance | 0.91408 | 0.2695 | small | STRESS RESPONSE, DEFENSE, VIRULENCE |
| Chorismate Synthesis | 0.91408 | 0.1925 | small | METABOLISM |
| Cobalamin synthesis | 0.91408 | 0.1797 | small | METABOLISM |
| Cytochrome d ubiquinol oxidase operon | 0.91408 | 0.1668 | small | ENERGY |
| Aminoglycoside modifying enzymes: O-phosphotransferases | 0.91408 | 0.1668 | small | STRESS RESPONSE, DEFENSE, VIRULENCE |
| Resistance to Capreomycin and Viomycin | 0.91408 | 0.1668 | small | STRESS RESPONSE, DEFENSE, VIRULENCE |
| p-Aminobenzoyl-Glutamate Utilization | 0.91408 | 0.1668 | small | METABOLISM |
| Rcs two-component regulator of capsule synthesis | 0.91408 | 0.1155 | small | CELL ENVELOPE |
| Biotin synthesis & utilization | 0.91408 | 0.1155 | small | METABOLISM |
| Nicotinic acid utilization | 0.91408 | 0.1027 | small | METABOLISM |
| Periplasmic disulfide interchange | 0.91408 | 0.0898 | small | PROTEIN PROCESSING |
| Fatty Acid Biosynthesis cluster | 0.91408 | 0.0642 | small | METABOLISM |
| Glutathione analogs: mycothiol | 0.91408 | 0.0642 | small | STRESS RESPONSE, DEFENSE, VIRULENCE |
| Anaerobic Oxidative Degradation of L-Ornithine | 0.91408 | 0.0642 | small | METABOLISM |
| Arsenic resistance | 0.91408 | 0.0642 | small | STRESS RESPONSE, DEFENSE, VIRULENCE |
| NAD and NADP cofactor biosynthesis global | 0.91408 | 0.0128 | small | METABOLISM |
| Diaminopimelate Synthesis | 0.91408 | 0 | small | METABOLISM |
| Tetracycline resistance, all mechanisms | 0.91408 | 0 | small | STRESS RESPONSE, DEFENSE, VIRULENCE |
| Arginine biosynthesis via N-acetyl-L-citrulline | 0.94839 | 0.2823 | small | METABOLISM |
| HipAB system implicated in growth arrest, persistence and drug tolerance | 0.94839 | 0.1412 | small | CELLULAR PROCESSES |
| Formate dehydrogenase | 0.94839 | 0.1283 | small | ENERGY |
| Phosphatidylinositol mannosides biosynthesis related cluster | 0.94839 | 0.1155 | small | CELL ENVELOPE |
| Cob(I)alamin adenosyltransferase | 0.94839 | 0.1027 | small | METABOLISM |
| Glutaconate CoA-transferase or 3-oxoadipate CoA-transferase Subunits | 0.94839 | 0.0898 | small | METABOLISM |
| Isoprenoid Biosynthesis: Interconversions | 0.94839 | 0.0898 | small | METABOLISM |
| Pyruvate metabolism I: anaplerotic reactions, PEP | 0.94839 | 0.0898 | small | ENERGY |
| tRNA aminoacylation, Thr | 0.94839 | 0.077 | small | PROTEIN PROCESSING |
| Fermentations: Lactate | 0.94839 | 0.0642 | small | ENERGY |
| Teicoplanin resistance in Staphylococci | 0.94839 | 0.0513 | small | STRESS RESPONSE, DEFENSE, VIRULENCE |
| Spore germination | 0.94839 | 0.0128 | small | CELLULAR PROCESSES |
| Sporulation gene orphans | 0.94839 | 0.0128 | small | CELLULAR PROCESSES |
| Sporulation proteins SigEG cluster | 0.94839 | 0.0128 | small | CELLULAR PROCESSES |
| Fatty acid metabolism cluster | 0.94839 | 0 | small | METABOLISM |
| tRNA aminoacylation, Met | 0.94839 | 0 | small | PROTEIN PROCESSING |
| Calvin-Benson cycle | 0.98279 | 0.385 | moderate | METABOLISM |
| Menaquinone biosynthesis from chorismate via 1,4-dihydroxy-2-naphthoate | 0.98279 | 0.2823 | small | METABOLISM |
| Coenzyme PQQ synthesis | 0.98279 | 0.2567 | small | METABOLISM |
| Formaldehyde assimilation: Ribulose monophosphate pathway | 0.98279 | 0.2182 | small | METABOLISM |
| Universal stress protein family | 0.98279 | 0.2053 | small | STRESS RESPONSE, DEFENSE, VIRULENCE |
| Biotin biosynthesis | 0.98279 | 0.1027 | small | METABOLISM |
| tRNA aminoacylation, Tyr | 0.98279 | 0.077 | small | PROTEIN PROCESSING |
| Lipopolysaccharide in K12 | 0.98279 | 0.0513 | small | CELL ENVELOPE |
| Resistance to Vancomycin and Teicoplanin | 0.98279 | 0.0128 | small | STRESS RESPONSE, DEFENSE, VIRULENCE |
| Biofilm Adhesin Biosynthesis | 0.98279 | 0 | small | CELLULAR PROCESSES |
| Programmed frameshift | 1 | 0.3208 | moderate | PROTEIN PROCESSING |
| NiFe hydrogenase maturation | 1 | 0.077 | small | ENERGY |
| Glutathione: Non-redox reactions | 1 | 0.0513 | small | STRESS RESPONSE, DEFENSE, VIRULENCE |
| Stress proteins YciF, YciE | 1 | 0.0513 | small | STRESS RESPONSE, DEFENSE, VIRULENCE |
